# Dynamic Combinatorial Chemistry Unveils Nsp10 Inhibitors with Antiviral Potential Against SARS‐CoV‐2

**DOI:** 10.1002/chem.202403390

**Published:** 2024-12-23

**Authors:** Ravindra P. Jumde, Gwenaëlle Jézéquel, Margarida Saramago, Nicolas Frank, Sebastian Adam, Marta V. Cunha, Chantal D. Bader, Antonia P. Gunesch, Natalie M. Köhler, Sandra Johannsen, Spyridon Bousis, Thomas Pietschmann, Rute G. Matos, Rolf Müller, Cecília M. Arraiano, Anna K. H. Hirsch

**Affiliations:** ^1^ Helmholtz Institute for Pharmaceutical Research Saarland (HIPS) – Helmholtz Centre for Infection Research (HZI) Campus E 8.1 66123 Saarbrücken Germany; ^2^ Current address Global Antibiotic Research & Development Partnership (GARDP) Chemin Camille-Vidart 15 1202 Geneva Switzerland; ^3^ Instituto de Tecnologia Química e Biológica António Xavier Universidade Nova de Lisboa Avenida da República 2780-157 Oeiras Portugal; ^4^ Saarland University Department of Pharmacy Campus E 8.1 66123 Saarbrücken Germany; ^5^ Institute for Experimental Virology Twincore – Centre for Experimental and Clinical Infection Research Feodor-Lynen-Str. 7 30625 Hannover Germany; ^6^ Cluster of Excellence RESIST (EXC 2155) Hannover Medical School 30625 Hannover Germany; ^7^ Helmholtz International Lab for Anti-infectives Campus E 8.1 66123 Saarbrücken Germany

**Keywords:** target-directed dynamic combinatorial chemistry, SARS-CoV-2, Nsp10, Nsp14, Nsp16

## Abstract

The development of antiviral drugs against the Severe Acute Respiratory Syndrome coronavirus 2 (SARS‐CoV‐2) responsible for the recent Covid‐19 pandemic is crucial, as treatment options remain limited and vaccination does not prevent (re)infection. Two relatively underexplored targets of this virus are the 3′‐5′ exoribonuclease (ExoN) and the 2’‐*O*‐methyltransferase (2′‐*O*‐MTase), both essential for viral viability. The non‐structural proteins Nsp14 and Nsp16 exhibit enzymatic activities for ExoN and 2′‐*O*‐MTase, respectively, especially when in complex with their co‐factor protein Nsp10. The study focuses on the use of target‐directed dynamic combinatorial chemistry (tdDCC) to identify binders of Nsp10, aiming to disturb the protein‐protein interactions (PPI) involving Nsp10‐Nsp14, as well as Nsp10‐Nsp16. We synthesised the hits and evaluated them to assess Nsp10 affinity, ExoN and 2′‐*O*‐MTase activities inhibition, and antiviral activity in hCoV‐229E and SARS‐CoV‐2‐infected whole‐cell settings. This study reports a novel class of ExoN and/or 2′‐*O*‐MTase inhibitors exhibiting antiviral activity against coronaviruses.

## Graphical abstract

This study investigates antiviral strategies targeting SARS‐CoV‐2 by disrupting the protein‐protein interactions of Nsp10 with Nsp14 and Nsp16, which are essential for viral survival. It employs target‐directed dynamic combinatorial chemistry for the identification and synthesis of inhibitors. Evaluation of these inhibitors for their binding affinity, enzymatic‐activity inhibition, and antiviral effects, offers novel insights for developing therapeutics against coronaviruses.

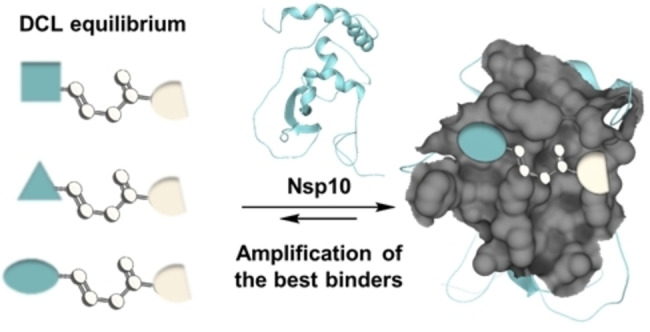

## Introduction

The end of 2019 saw the emergence of the Severe Acute Respiratory Syndrome coronavirus 2 (SARS‐CoV‐2), a new coronavirus that led to the Covid‐19 pandemic.[^1^, ^2^] The fast development of vaccines lowered the impact of the outbreak, especially on overwhelmed healthcare facilities.^[3]^ However, there is still a need for SARS‐CoV‐2 therapeutics due to the persistent number of breakthrough infections caused by the emergence of virus variants, incomplete vaccination, and the difficulty in developing specific/potent drugs, including those derived from drug repurposing.^[4]^ Some approved drugs have been repurposed for the treatment of Covid‐19, with limited success, except for the combination of nirmatrelvir/ritonavir (Paxlovid) for which the FDA granted an emergency use authorisation.[^5^, ^6^] Remdesivir remains the only fully FDA‐approved treatment option for COVID‐19 so far.

SARS‐CoV‐2 is a positive‐sense single‐stranded RNA virus, and codes for four structural proteins, and two polyproteins.^[7]^ After cleavage by the main protease (M^pro^), those polyproteins yield 16 nonstructural proteins (Nsp) having RNA‐processing and RNA‐modifying functions essential for viral replication.^[8]^ Among these Nsps, Nsp14 is a bifunctional non‐structural protein displaying two essential activities: an N‐terminal 3′‐5′ exoribonuclease activity (ExoN) and a C‐terminal N7‐methyltransferase activity (N7‐MTase), located on two distinct domains.^[9]^ Interestingly, the ExoN activity is specific and crucial to coronaviruses, as it ensures reliability of the replication process.^[10]^ SARS‐CoV‐2 ExoN knockout mutants are not viable, contrary to the SARS‐CoV‐1 and Mouse Hepatitis Virus (MHV) knockout mutants that show a high mutation frequency, but no lethality.[^11^, ^12^, ^13^] Inhibiting this enzymatic activity therefore appears to be a relevant strategy to develop new specific antivirals against SARS‐CoV‐2. Strikingly, the ExoN activity of Nsp14 is strongly stimulated in a dose‐dependent manner by the formation of a stable complex with Nsp10, which acts as a cofactor.^[14]^ While Nsp14 alone still displays an exoribonuclease activity, it is orders of magnitude lower than when in complex with Nsp10.^[15]^

It is interesting to note that Nsp10 acts as a cofactor for Nsp16 as well, to display a 2’‐*O*‐methyltransferase (2’‐*O*‐MTase) activity.^[16]^ The 2′‐*O*‐MTase forms part of the replication‐transcription complex, and its binding partner, Nsp10, regulates its activity. It plays an essential role in host immune evasion by mimicking its human homolog, Cap‐specific mRNA(nucleoside‐2′‐*O*‐)‐methyltransferase (CMTr1), to perform a crucial step in capping transcribed mRNA.[^16^, ^17^] The 2′‐*O*‐MTase knockout mutants show attenuation of viral replication and infectivity of coronaviruses in the host.[^18^, ^19^]

Nsp10 is therefore an essential protein for viral replication, though it does not have an activity *per se*, but is a crucial activation factor for both Nsp14 and Nsp16.^[20]^ Finding disturbers of the Nsp10‐Nsp14 and/or Nsp10‐Nsp16 protein‐protein interactions (PPI), targeting both the ExoN and MTase activities, represents a promising strategy to develop new antivirals against SARS‐CoV‐2. Moreover, availability of crystal structures of Nsp10, either alone or in complex with Nsp14 or Nsp16 provided a good starting point for target‐directed drug development.[^21^, ^22^, ^23^]

Nsp14 is known to reduce the inhibitory effect of drugs that function through premature termination of viral genome replication.[^10^, ^13^, ^24^, ^25^, ^26^] The only fully approved FDA drug Remdesivir is an antiviral that belongs to the type of nucleoside analogue inhibitors (NAs) targeting the RNA‐dependent RNA‐polymerase (Nsp12 in CoVs), and its use for coronavirus is challenging due to the presence of the Nsp14 ExoN proofreading activity.^[27]^ Taking this into account, Nsp14 is particularly important as a drug target.^[4]^ Some efforts to find inhibitors of ExoN activity in SARS‐CoV‐2, either by *in silico* screening, high‐throughput biochemical screening or fragment approaches led to the discovery of interesting compounds.[^28^, ^29^, ^30^, ^31^] However, the mode of action of some of the identified hits is not clear, and all will need further optimisation in terms of activity and selectivity. Similarly, for the 2′‐*O*‐MTase target, most of the reported inhibitors are adenosyl analogues, which suffer from selectivity issues for viral over human MTases causing (cyto)toxic effects. Despite recent medicinal‐chemistry efforts, only very few non‐adenosyl inhibitors are reported with questionable activity, often based on virtual docking scores, or showing poor selectivities.[^32^, ^33^, ^34^]

To target the ExoN and MTase activities, we set out to find new hits that would inhibit the PPIs of these proteins with Nsp10. Due to the lack of potent inhibitors of these enzymes of SARS‐CoV‐2, alternative hit‐identification strategies that go beyond traditional medicinal‐chemistry approaches are required. One of the hit‐identification strategies that does not require the knowledge of inhibitors is target‐directed dynamic combinatorial chemistry (tdDCC), which has emerged as an efficient hit‐identification strategy over the past two decades.[^35^, ^36^, ^37^] In protein‐templated DCC in particular, a dynamic combinatorial library (DCL) is incubated with the target protein. All the products pre‐form in this DCL, in different proportions. The protein modifies the DCL equilibrium by selecting and amplifying its binders within the library. Final analysis is made by integration of the relative peak area of UV absorption for each product, and the calculation of amplification in presence of the target. This powerful tool already allowed the fast discovery and optimisation of new inhibitors for various targets, including PPI targets.[^38^, ^39^, ^40^, ^41^]

## Results and Discussion

Nsp10 emerged as a suitable target protein for our tdDCC approaches for several reasons. It is stable and soluble at room temperature, as confirmed by thermal‐shift experiments (for details see SI). Moreover, Nsp10 is small (140 amino acids) with well‐defined PPI domains that interact with Nsp14 and Nsp16.^[14]^ Given that Nsp10 serves as a cofactor for both proteins, targeting it through tdDCC may lead to the discovery of inhibitors for either Nsp14 or Nsp16, or even dual inhibitors that target both proteins simultaneously.

### Set‐Up of the tdDCC

**Figure 1:**
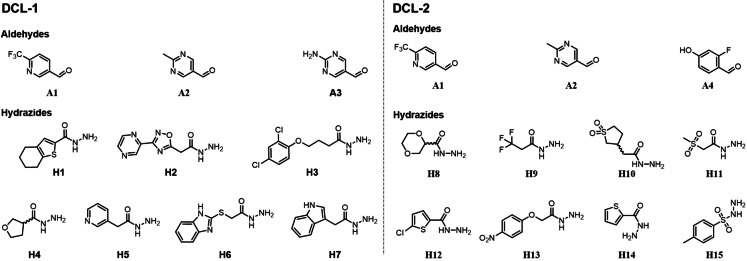
Dynamic combinatorial libraries (DCLs) used for tdDCC‐1 and tdDCC‐2. For both tdDCCs, the experiment was run in phosphate buffer (pH 7.04) and 5 % DMSO with the aldehydes (100 μm each, in DMSO), hydrazides (300 μm each, in DMSO), aniline (10 mm in DMSO) and the Nsp10 protein (50 μm in phosphate buffer).

To ensure a broad range of structural diversity, we performed two rounds of tdDCC experiments using different DCLs. Both DCL‐1 and DCL‐2 consisted of three aldehydes and seven (**H1**–**H7**) or eight (**H8**–**H15**) hydrazides, respectively (Figure 1). We carefully selected a set of aromatic aldehydes and a wide range of hydrazide building blocks, including various aromatic and heterocyclic rings, each bearing different functional groups and linkers. We included aromatic rings with electron‐withdrawing (nitro, dichloro, fluoro and trifluoromethyl) and ‐donating (hydroxyl, amine, and methyl), as well as polar (sulfonyls, dioxane, nitro, and hydroxyl) groups to study their impact on the biological activity of the potential hits under physiological conditions. To explore the pocket size, we employed flexible linkers of different lengths (zero to four carbons between the hydrazide and adjacent moieties).

In both tdDCC experiments, we followed the same procedure. Reaction of the building blocks in phosphate buffer with an excess of aniline and 5 % DMSO led to the DCLs, with aniline accelerating the slow acylhydrazone formation at neutral pH. For each tdDCC, we prepared three sets of reaction mixtures. One without the Nsp10 protein (blank) and two duplicates in presence of Nsp10 protein (namely, “protein‐templated I” and “protein‐templated II”). To study the equilibrium of acylhydrazone formation, we analysed the composition of blank DCL using UPLC‐MS. The periodic samples from blank and protein‐templated DCLs were treated with NaOH to freeze the acylhydrazone formation and with acetonitrile to denature the protein. After reaching the equilibrium, comparison of an adaptive DCC experiment in presence of Nsp10 protein with the corresponding blank DCL enabled identification of the amplified acylhydrazones.

### tdDCC‐1

**Figure 2:**
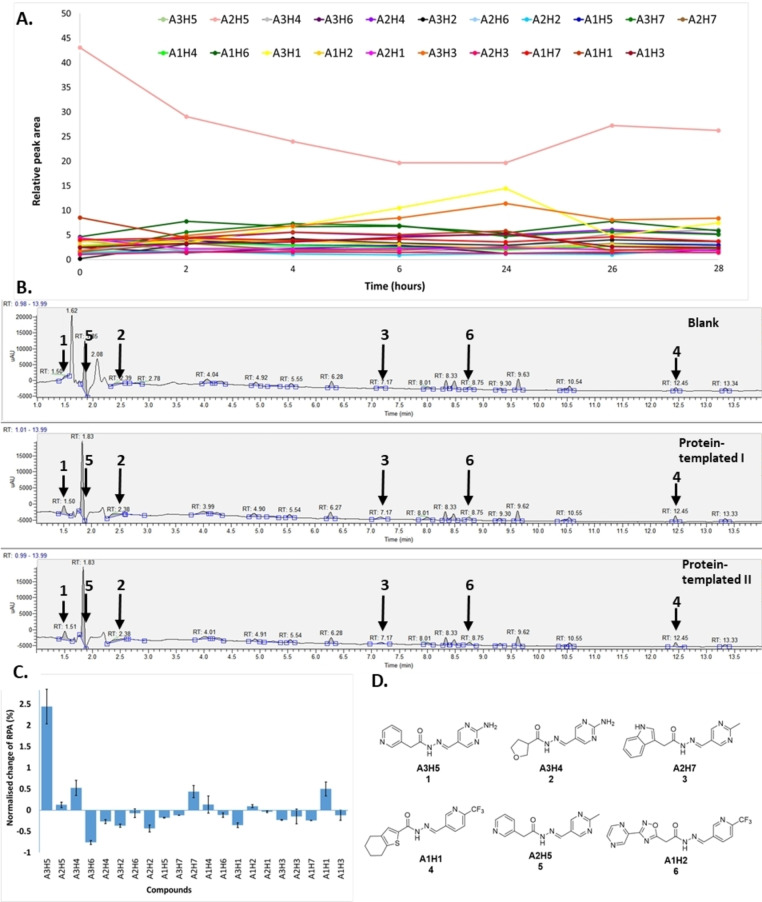
tdDCC‐1. A – Evaluation of the relative peak areas of different acylhydrazone concentrations over time and determination of the equilibrium. B – Comparison of relative peak areas between the blank and the protein‐templated experiments. C – Normalised relative amplification of the products in the protein‐templated DCC experiments compared to the blank. D – Structure of the significantly and slightly amplified hits.

In a tdDCC experiment, determining the time required to reach equilibrium is a crucial step. For this purpose, we identified all the products in the mixture (21 acylhydrazones) by their respective mass from the UPLC‐MS runs, and the evolution of their relative peak area (RPA) was plotted as a function of time. The system is considered to have reached a state of equilibrium when there are no significant changes in the relative peak areas of the various acylhydrazones.^[39]^ For tdDCC‐1, this equilibrium was reached after 26 hours (Figure 2a). We then compared the RPAs of the two protein‐templated duplicates with the blank to assess the amplification of the respective compounds (Figure 2b and 2c). We identified four significantly amplified hits (**1**–**4**) with an RPA change over 0.5 %, two slightly amplified with an RPA change between 0 and 0.5 % (**5** and **6**) (Figure 2d) and decided to include two random depleted hits (**7**, **8**) as a control (Figure 4a). Analysing the structures of amplified hits, we observed the presence of trifluoromethylpyridine (**A1**), methyl‐ (**A2**) and amino‐pyrimidines (**A3**) moieties in the aldehyde part among the hits. Out of them, the 2‐aminopyrimidine moiety appeared in the top two amplified hits (**1** and **2**), followed by 2‐methylpyrimidine (**3** and **5**) and 2‐trifluoromethylpyridine (**4** and **6**). In terms of the hydrazide part, the pyridine‐containing hydrazide **H5** seems to be more favoured as it appeared twice in hits **1** and **5**. Hydrazides **H1**, **H2**, **H4**, and **H7** were present in hits **4**, **6**, **2**, and **3**, respectively. The two hydrazides **H3** and **H6** did not appear in any of the amplified hits. Furthermore, both electron‐rich (**H4**) and electron‐poor (**H5**) hydrazides are featured in the hits **2** and **1** and **5**, respectively.

### tdDCC‐2

Similarly to tdDCC‐1, the equilibrium for tdDCC‐2 was determined after the identification of the products in the mixture. However, in the analysis, we excluded the compound **A1H9** from consideration because, although it could be detected by MS, its formation was most likely very low and undetectable by UV absorption. Compounds **A1H15** and **A2H15** were not detected by LCMS, probably due to the decomposition during the basic treatment and/or during the ionization. In contrast to the acylhydrazones, the proton adjacent to the nitrogen atom in the *p*‐toluenesulfonylhydrazones is susceptible to the base attack, which can lead to the formation of an unstable diazo compound.^[42]^ However, we could detect a trace amount of compound **A4H15** by MS, which also contains a *p*‐toluenesulfonyl hydrazine moiety, although it was co‐eluted with compound **A1H14**. This situation occurred three times in the analysis. To address this issue, in the equilibrium and amplification analysis, we treated the co‐eluded compounds as one product (**A4H15**+**A1H14**, **A4H8**+**A2H14** and **A4H14**+**A4H9**) and considered any positively amplified compounds as potential hits. This highlights a challenge associated with large‐scale tdDCC, where different products may elute at the same retention time.

**Figure 3:**
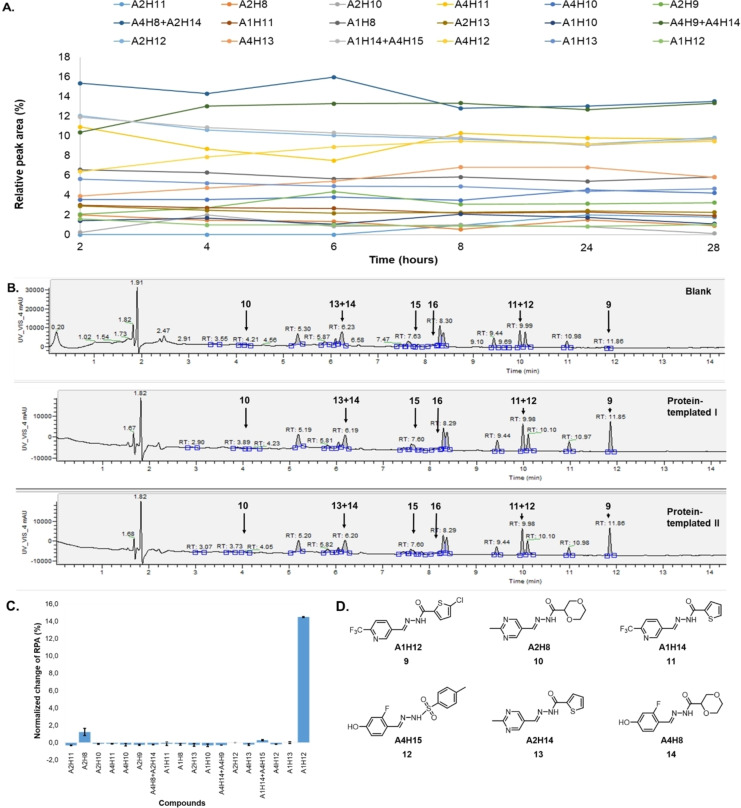
tdDCC‐2. A – Evaluation of the relative peak areas of different acylhydrazone concentrations over time and determination of the equilibrium. B – Comparison of relative peak areas between the blank and the protein‐templated experiments.. C – Normalised relative amplification of the products in the protein‐templated DCC experiments compared to the blank. D – Structure of the significantly and slightly amplified hits.

**Figure 4:**
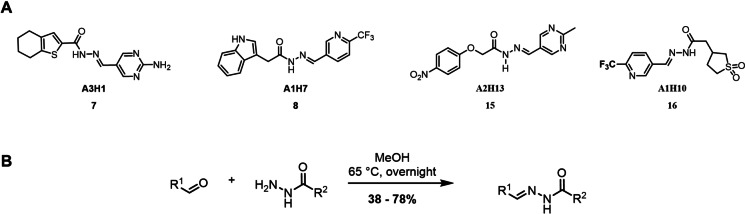
A – Selected controls from depleted compounds for tdDCC‐1 (**7** and **8**) and tdDCC‐2 (**15** and **16**). B – General scheme for the synthesis of acylhydrazones. Reaction conditions: the hydrazide (1.0 equiv.) and the aldehyde (1.0 equiv.) are suspended in MeOH and stirred overnight at 65 °C.

For tdDCC‐2, the equilibrium was reached after eight hours (Figure 3a), enabling the comparison of the RPAs between the blank and protein‐templated experiments (Figure 3b) and the calculation of the amplification (Figure 3c). This analysis revealed that compound **9** was significantly amplified, and compound **10** was slightly amplified in the tdDCC‐2 experiment (Figure 3d). The peak corresponding to the two compounds **11** and **12** was also slightly amplified, hence, we consider both as potential hits. Finally, compounds **13** and **14** are slightly depleted, and no definitive conclusion could be drawn from the amplification analysis. We chose two compounds, **15** and **16**, among the depleted amplifications, as a negative control (Figure 4a). Additionally, we also chose compounds **13** and **14** for synthesis, which were co‐eluted and are slightly depleted. Similar to tdDCC‐1, the hit compounds in tdDCC‐2 exhibit a pattern where each of the three different aldehydes (**A1**, **A2**, **A4**) is represented twice among the hit compounds. Regarding the hydrazide part, the thiophene or chlorothiophene moieties are present in three out of the six hits (**9**, **11**, **13**), suggesting their significant role in the amplification process. Hydrazides containing polar group (**H8)** in compound **8**, **10** and apolar groups (**H12** and **H14**) in compounds **14, 11** and **13**, respectively, were amplified. Notably, both electron‐rich (**H12**, **H14**) and electron‐poor (**H15**) hydrazides contributed to the amplification of compounds **9**, **11**, **13** and **12**, respectively.

To validate the activity of the identified hits, we selected the significantly and slightly amplified hits (**1**–**6** and **9**–**12**, Figures 2d and 3d), and included two random controls among the depleted compounds (**7**, **8**, **15**, and **16**, Figure 4a) for each DCL. We also synthesised compounds **13**–**14** (Figure 3d) to assess their actual activity. We accessed all the selected acylhydrazone hits and the control compounds in one step, by reacting aldehyde and corresponding hydrazide in methanol at 65 °C, in moderate to excellent yields (Figure 4b).^[38]^

### Binding Affinity of tdDCC‐1 & ‐2 Hits

**Figure 5:**
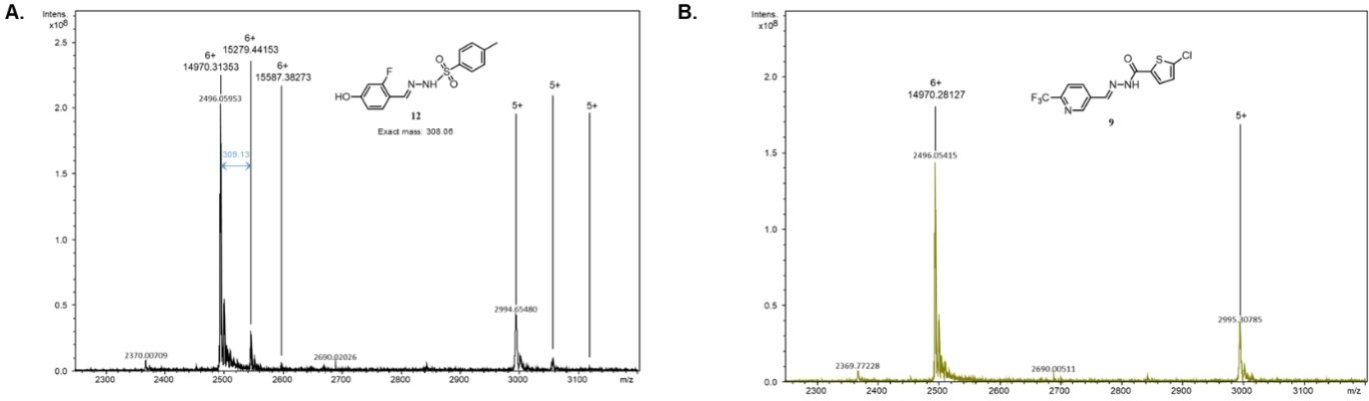
Representative native MS spectra for some tdDCC hits. A – Analysis of compound **12** with low binding affinity displays a complex mass peak with a low intensity compared to the protein mass peak. B – Analysis of non‐binding compound **9** does not show a mass peak corresponding to the complex mass.

We evaluated all the synthesised compounds (**1**–**16**) for their binding affinity to Nsp10 by a native mass spectrometry (MS) analysis. Native MS is a versatile method that enables the analysis of proteins and their non‐covalently driven assemblies in their native or near‐native state by spraying them from non‐denaturing solvents.^[43]^ Thus, if the ligand binds to the protein, the mass of the complex is detected with an intensity proportional to the binding affinity. It is an easy, efficient, and quick method that can be used to do high‐throughput screening of potential inhibitors. Most of the synthesised compounds bind Nsp10, as evidenced by the detection of the corresponding complex mass in native MS. However, compounds **2**, **7** and **9** did not exhibit binding and compound **16** showed non‐specific binding (Table 1). Despite the binding we observed for the compounds, the intensity of the protein−ligand complex peak was low compared to the intensity of the protein peak alone, indicating a weak binding affinity (Figure 5).

**Table 1:** Binding affinity of the tdDCC hits with Nsp10 determined by native MS and SPR analysis.^[a]^

Compound	Native MS	*K*_D_ on Nsp10 (μm)	Compound	Native MS	*K*_D_ on Nsp10 (μm)
1	weak intensity	15±3	9	complex not detected	n.b.
2	complex not detected	160±40	10	weak intensity	n.b.
3	weak intensity	100±10	11	weak intensity	n.b.
4	weak intensity	90±10	12	weak intensity	200±60
5	weak intensity	80±10	13	weak intensity	n.b.
6	weak intensity	120±10	14	weak intensity	n.b.
7	complex not detected	170±20	15	weak intensity	n.b.
8	weak intensity	130±10	16	unspecific binding	n.b.

*[a] The affinity of the synthesised compounds to Nsp10 was measured by Surface Plasmon Resonance with a 24 μm concentration of Nsp10 in sodium acetate buffer. n.b.: no binding*

To further characterise the binding affinity of the compounds, we conducted an orthogonal analysis using Surface Plasmon Resonance (SPR). Compounds **1**–**8** showed a double‐digit micromolar affinity (*K*_D_: 15–170 μm), while compound **12** had a *K*_D_ of 200 μm. The remaining compounds did not demonstrate significant binding to Nsp10 by SPR (Table 1). The presence of the 2‐aminopyrimidine motif appeared to enhance the affinity towards Nsp10, as compounds **1** and **7**, which contain this motif, are the two best binders. Moreover, compounds **1** and **5**, which possess a pyridine moiety, exhibited good *K*_D_ values. However, the comparatively smaller hits from tdDCC‐2 (**9**–**11**, **13**, **14**) did not show affinity towards Nsp10, except for slightly heavier compound **12**. These findings suggests that the hydrazide part should have at least one aromatic ring with a spacer to enable binding to Nsp10 (excluding compound **2**, which displayed a good affinity despite its smaller size). We observed that when the *K*_D_ values ranged from 15–100 μm, a weak intensity of the near‐native MS signal was detected. However, above 100 μM, the correlation does not exist anymore, and we can witness false positives and negatives. While near‐native MS can serve as an initial screening method for the binding affinity, SPR is complementary and essential to accurately assess the actual affinity.

### Inhibitory Effect of tdDCC Hits on ExoN Activity of SARS‐CoV‐2

**Figure 6:**
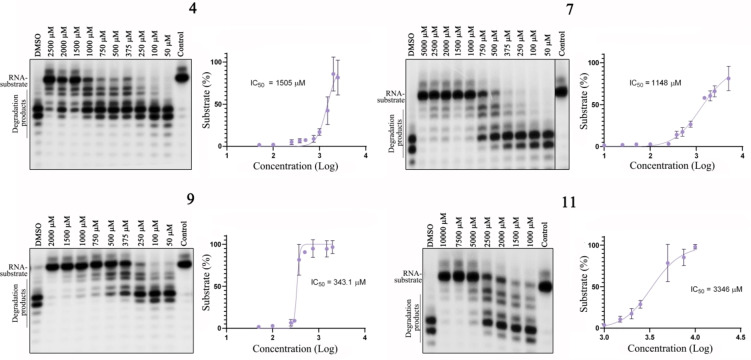
Inhibition of the 3‘‐5‘ exoribonuclease activity of SARS‐CoV‐2 Nsp14 by compounds **4, 7, 9** and **11**. 500 nm of Nsp14 and 2000 nm of Nsp10 were incubated with 50 nm of RNA substrate in the presence of 50 to 10,000 μm of each compound. Reactions were analysed on 7 m urea/20 % polyacrylamide gels. C, control reactions with no enzymes; DMSO, reactions performed in the absence of any compound but with DMSO. All the experiments were performed at least in triplicate. The amount of RNA substrate at the end of the reaction was quantified in the presence of different concentrations of each compound, and the IC_50_ was determined for each compound.

We evaluated the inhibitory effects of our designed compounds on the ExoN activity of Nsp14 *in vitro*, specifically targeting the PPI site of the Nsp10/Nsp14 complex. For that, all the compounds were previously incubated with Nsp10 for a few minutes and then we allowed the reaction to proceed. The results have shown that compound **9** significantly inhibits the ExoN activity of Nsp14 (Figure 6). Compounds **4**, **7** and **11** also showed a decrease in Nsp14’s ability to degrade the RNA substrate. Each compound was titrated to determine the respective inhibitory concentration (IC) *in vitro* (Figure 6). Compound **9** exhibited the lowest IC_50_ value among the others, indicating its potential to inhibit Nsp14 ExoN activity. Additionally, we conducted experiments where compound **9** was incubated with a pre‐formed Nsp14/Nsp10 complex. Interestingly, this compound was able to disrupt the ExoN activity even in these conditions (Supplementary Figure 1). Compounds **4**, **9** and **11**, which share the 2‐(trifluoromethyl)pyridine motif, appears to be advantageous for inhibiting the ExoN activity. These compounds also feature a thiophene moiety, including simple thiophene (**11**), a chlorothiophene (**9**) or a tetrahydrobenzothiophene (**4** and **7**). The presence of an electron‐rich apolar motif on this side of the molecule appears highly beneficial for exerting inhibitory activity.

### Inhibitory Effect of tdDCC Hits on the MTase Activity of SARS‐CoV‐2

**Figure 7:**
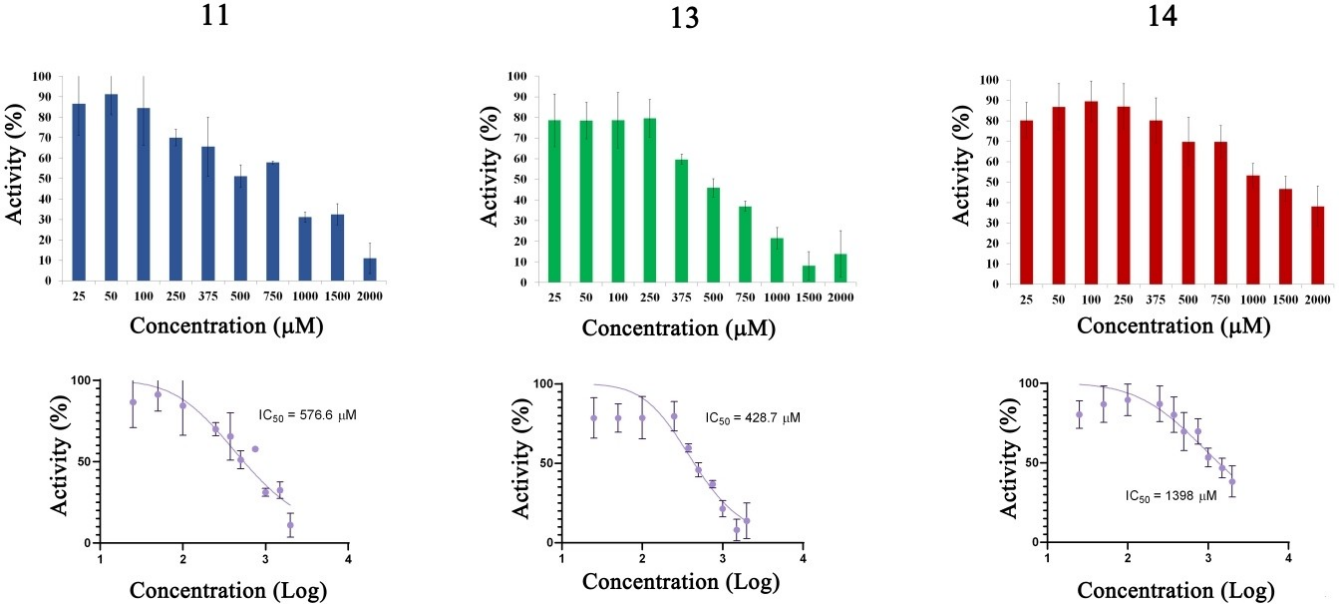
Inhibition of the 2’‐*O*‐methyltransferase activity of SARS‐CoV‐2 Nsp16 by compounds **11**, **13** and **14**. 500 nm of Nsp16 and 2000 nm of Nsp10 were incubated with 50 nm of capped RNA substrate in the presence of 25 to 2000 μM of each compound. The formation of the reaction product SAH was determined by luminescence. Control reactions with no enzymes and with the enzymes in the presence of DMSO were performed to determine 0 and 100 % of activity. All the experiments were performed at least in triplicate. The IC_50_ was determined for each compound.

Considering that we designed the tdDCC to find binders of Nsp10, which is also a co‐factor for Nsp16, our compounds could potentially also interfere with the Nsp10/Nsp16 PPI site. Moreover, as some residues involved in the Nsp10/Nsp14 and Nsp10/Nsp16 interactions (Gly69, Ser72, Tyr96) are the same, certain compounds may exhibit dual inhibition.^[44]^ Considering this, we tested all 16 hit compounds to assess their inhibitory potential on the MTase activity of Nsp16/Nsp10. Among them, compounds **11**, **13** and **14** demonstrated dose‐dependent inhibition of the MTase activity (Figure 7). Notably, compound **13** exhibited the lowest IC_50_ value (429.7 μm). Interestingly, compound **11** displayed inhibitory effect on both the ExoN and MTase activities of Nsp14/Nsp10 and Nsp16/Nsp10 complexes, with greater potency against the Nsp16 MTase (Figures 6 and 7). These results highlight that compound **11** has a dual role and therefore it is a good candidate for further development as a dual inhibitor.

### Whole‐Cell Antiviral Activity Against hCoV‐229E

**Figure 8:**
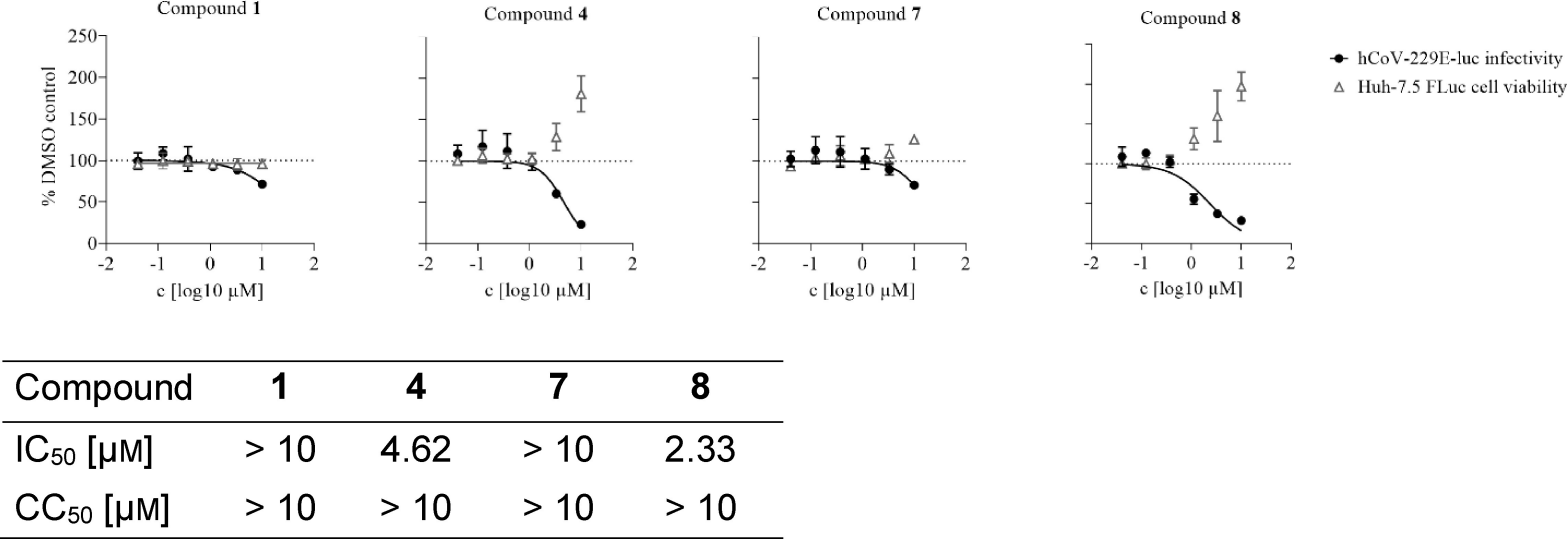
Activity of compounds **1**, **4**, **7** and **8** against hCoV‐229E‐luc infectivity and cell viability. Huh‐7.5 FLuc cells were infected with hCoV‐229E‐luc for 48 h in the presence of indicated compound concentrations. Renilla luciferase activity was determined as measure for residual infectivity (black circles) whereas firefly luciferase activity of the same cells represents cell viability (grey triangles). The mean and standard deviation of three biological replicates are depicted. IC_50_ and CC_50_ values were interpolated from regression curves.

Finally, we assessed the potential antiviral activity of tdDCC hits agents against coronaviruses. We tested them against human coronavirus 229E (hCoV‐229E), a member of the alphacoronavirus family responsible for causing the common cold.^[45]^ SARS‐CoV‐2 (from a betacoronavirus family) and hCoV‐229E share 67 % of sequence similarity and 70 % for Nsp14 specifically.^[46]^ Interestingly, hCoV‐229E Nsp14 knockout mutants are also not infectious.^[47]^ SARS‐CoV is closer in structural similarity to SARS‐CoV‐2 (80 % of sequence similarity and 95 % for Nsp14) but its Nsp14 mutants are still viable.^[13]^ This makes hCoV‐229E a good and easy‐to‐handle model for a first phenotypic evaluation, whereas SARS‐CoV‐2 assays require specific infrastructure and equipment. We performed a dual luciferase assay in a whole‐cell setting with hepatocarcinoma cell lines (Huh‐7.5 FLuc) infected with hCoV‐229E‐luc *renilla* luciferase reporter virus and measured residual infectivity and cell viability after incubation with the compounds.^[48]^ Compounds **4** and **8** showed dose‐dependent antiviral activity with low micromolar half‐maximal inhibitory concentrations (IC_50_=4.6 and 2.3 μm, respectively), without showing any cytotoxicity (Figure 8). None of the other tested compounds showed any significant antiviral activity on hCoV‐229E. Once again, we observed the presence of 2‐(trifluoromethyl)pyridine and the 2‐aminopyrimidine moieties in the active compounds, reaffirming their positive effect within this class, compared to the two other aldehydes. Additionally, compound **4** contained a tetrahydrobenzothiophene, while compound **8** featured an indole moiety. Compound **1** being the least active, suggesting that electron‐rich aromatic rings are more beneficial than electron‐poor ones.

Eight of the synthesised compounds showed interesting although different profiles in terms of binding with Nsp10 and/or inhibition of enzymatic activities and were therefore tested for their antiviral activity against SARS‐CoV‐2 full‐length virus in Calu‐3 cells (Table 2). Compounds **4**, **7**, **8** and **14** led to a moderate decrease in the infectivity of the virus (67–88 % compared to the control at 10 μM). Compounds **4** and **7** exhibited promising activities across multiple assays, displaying favorable binding affinity, inhibiting ExoN activity, and reducing infectivity of SARS‐CoV‐2 in Calu‐3 cells, without showing any cytotoxicity in the whole‐cell setting. Compound **4** also displays a micromolar antiviral activity against hCoV‐229E. Compounds **1** and **8** bind to Nsp10 but failed to inhibit any of the two tested enzymes. However, compound **8** showed the best antiviral activity in both hCoV‐229E and SARS‐CoV‐2 infectivity assays. It may therefore have another mode of action, which warrants further investigation. Compounds **9**, **13** and **14** show an interesting profile, as they did not bind to Nsp10 and did not exhibit any antiviral effect against hCoV‐229E. However, they effectively inhibited either the ExoN or MTase activity. Compound **14** demonstrated antiviral activity against SARS‐CoV‐2. Notably, compound **9** showed the highest efficiency in inhibiting the ExoN activity, and was the only compoundable to disrupt the pre‐formed complex. Compound **11**, despite not binding to Nsp10 and lacking antiviral effect against the tested coronaviruses, efficiently inhibited both EXoN and MTase activities. These results suggest that four compounds might act as PPI inhibitors targeting regions of the protein that are not conserved between SARS‐CoV‐2 and hCoV‐229E. The lack of activity in the whole‐cell assay may be influenced by factors such as permeability issues, although other mechanisms should not be ruled out and require further investigation.

**Table 2:** Binding affinity for Nsp10, antiviral activities (hCoV‐229E and SARS‐CoV‐2), cytotoxicity and inhibition of the ExoN and MTase activity of the most active hits obtained from tdDCC experiments 1 and 2.

	Compound	*K*_D_ on Nsp10 (μm)	IC_50_ against the ExoN activity (μm)	IC_50_ against the MTase activity (μm)	IC_50_ against hCoV‐229E (μm)	CC_50_ (μm)	Single dose 10 μm mean infectivity on SARS‐CoV‐2 [% control]
	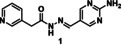	15±3	no activity	no activity	>10	>10	100
	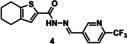	90±10	1505	no activity	4.6	>10	83
	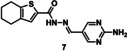	170±20	1148	no activity	>10	>10	81
	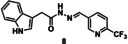	130±10	no activity	no activity	2.3	>10	67
	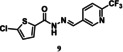	no binding	343	no activity	>10	>10	119
	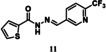	no binding	3346	577	>10	>10	120
	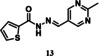	no binding	no activity	429	>10	>10	88
	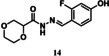	no binding	no activity	1398	>10	>10	70

In general, the beneficial structural motifs are on the aldehyde side the 2‐trifluoromethylpyridine and the 2‐aminopyrimidine moieties, and on the hydrazide side thiophene, chlorothiophene, and tetrahydrobenzothiophene. These motifs are therefore promising starting scaffolds that opens the path for further optimisation of the compounds, especially towards a better phenotypic activity.

## Conclusions

In this work, we performed two tdDCC experiments with the objective of discovering potential antiviral compounds targeting Nsp10 of SARS‐CoV‐2. Nsp10, despite lacking any enzymatic function has a pleiotropic effect, since it is a co‐factor of both Nsp14 and Nsp16, and targeting it can inhibit the ExoN and the MTase activity associated with these enzymes, respectively. Sixteen of the identified hits and controls were synthesised and evaluated for their affinity towards Nsp10 enzymatic activity against ExoN and MTase, and antiviral activity against hCoV‐229E and SARS‐CoV‐2. The tdDCC approach effectively enabled the rapid identification of binders of Nsp10, making them potential PPI inhibitors. In total, eight of the amplified hits displayed interesting activities, with compounds **4** and **7** being active in most assays, and compound **11** inhibiting both the ExoN and MTase activities of Nsp14 and Nsp16, respectively. These compounds exhibited good to moderate binding affinity for Nsp10 (*K*_D_=15–200 μm), disturbing the PPI between Nsp10/Nsp14 or Nsp10/Nsp16, resulting in moderate inhibition of the ExoN and MTase activity (IC_50_=343–3346 μm). Furthermore, these compounds displayed significant antiviral activity against hCoV‐229E (IC_50_=2.3–4.6 μm) and/or against SARS‐CoV‐2 (67–88 % infectivity compared to the control at 10 μm). Additionally, it is worth noting that two of the control unamplified compounds exhibit moderate binding affinity for Nsp10 (compound **8**) and enzymatic activity (compound **11**). These findings highlight the importance of including control compounds in the screening process and suggest the potential for alternative mechanisms of action.

Although the hits obtained in this study showed good to moderate binding affinity, moderate enzymatic, and *in vivo* activity, they represent the first example of a new class of inhibitors of ExoN and MTase activity, with potential antiviral properties. This study represents the first example of tdDCC to identify inhibitors of SARS‐CoV‐2. Further optimisation of these hits is necessary to improve their activity and ADMET properties, and the mode of action should be validated. This work lays a foundation for a medicinal‐chemistry program aimed at developing these hits into effective antiviral agents for SARS‐CoV‐2 and potential future coronaviruses.

## Experimental Section

**General information**. All reactions using oxygen‐ and/or moisture‐sensitive materials were carried out in dry solvents (vide infra) under a nitrogen atmosphere using oven‐dried glassware. Reactions were monitored by a Liquid chromatography‐mass spectrometry (LC–MS) system equipped with Dionex UltiMate 3000 pump, autosampler, column compartment, detector, and ESI quadrupole MS (MSQ Plus or ISQ EC) from Thermo Fisher Scientific, Dreieich, Germany. Purification of the final products, when necessary, was performed using preparative HPLC (Dionex UltiMate 3000 UHPLC+ focused, Thermo Scientific) on a reversed‐phase column (C18 column, 5 μM, Macherey‐Nagel, Germany). The solvents used for the chromatography were water (0.1 % formic acid) and MeCN (0.1 % formic acid). High‐resolution mass (HRMS) of final products was determined by LCMS/MS using a Thermo Scientific Q Exactive Focus Orbitrap LC–MS/MS system. NMR data were collected on a Bruker Avance Neo 500 MHz (1H at 500.0 MHz; ^13^C at 126.0 MHz; ^19^F NMR at 470 MHz), equipped with a Prodigy Cryo‐probe. Chemical shifts are reported in parts per million (ppm) relative to residual solvent peak (DMSO‐d_6_, ^1^H: 2.54 ppm; ^13^C: 39.9 ppm). Coupling constants are reported in Hertz (Hz). Multiplicity is reported with the usual abbreviations (s: singlet, br s: broad singlet, d: doublet, dd: doublet of doublets, ddd: doublet of doublet of doublets, t: triplet, dt: doublet of triplets, q: quartet, p: pentet, dp: doublet of pentets, m: multiplet).

The periodic progress and analysis of DCC were monitored by UPLC‐MS (ThermoScientific Dionex Ultimate 3000 UHPLC System coupled to a ThermoScientific Q Exactive Focus with an electrospray ion source) using an Acquity Waters Column (BEH, C8 1.7 μm, 2.1×150 mm, Waters, Germany) at a flow rate of 0.250 mL/min with detection set at 210, 254, 290, and 310 nm, and the mass spectrum recorded in a positive mode in the range of 100–700 m/z. The solvent system was 0.1 % formic acid in H_2_O (Solvent‐A) and 0.1 % formic acid in MeCN (Solvent‐B). The gradient program began with 5 % of Solvent‐B for 1 min and was then increased to 95 % of Solvent‐B over 17 min and held for 2 min, followed by a decrease of Solvent‐B to 5 % over 0.1 min, where it was held for 2 min.

**Chemicals**. Unless indicated otherwise, reagents and substrates were purchased from commercial sources and used as received. Solvents not required to be dry were purchased as technical‐grade and used as received. Dry solvents were purchased from commercial sources in Sure/SealTM bottles and used as received and stored under a dry inert gas (N_2_ or Ar). Inert atmosphere experiments were performed with standard Schlenk techniques with dried (P2O5) nitrogen gas. Unless indicated otherwise, the acylhydrazone products were purified by precipitation of the product at a lower temperature followed by filtration and successive washing with cold solvents to remove soluble impurities (for details, see purification method‐1). All reported compounds were characterised by ^1^H and ^13^C NMR and compared with literature data. All new compounds were fully characterised by ^1^H and ^13^C NMR and HRMS techniques. All compounds have been determined to be >95 % pure by HPLC analysis. Acylhydrazones exist as a mixture of conformers, and the purity assessment takes into consideration all conformers present in equilibrium, as previously described.^[49]^

**DCL preparation (GP‐1)**. DCL without protein is prepared according to the published method.^[38]^

To a 1.5 mL Eppendorf Tube® containing phosphate buffer (pH 7.04) were added hydrazides (300 μM each, in DMSO), aldehydes (100 μM each, in DMSO), aniline (10 mM, in DMSO), and an additional amount of DMSO to reach a final concentration of 5 % in the DCL with 1 mL of end‐volume. The DCL was allowed to gently mix on a rotating wheel (7 rpm) at room temperature and was frequently monitored via UPLC‐MS. For analysis, 20 μL of the corresponding library was mixed with 80 μL acetonitrile and 4 μL of NaOH (1 M), the mixture was centrifuged, and the supernatant was used for the analysis.

**Protein‐templated DCL preparation (GP‐2)**. DCL with protein is prepared according to the published method.^[38]^ To a 1.5 mL Eppendorf Tube® containing phosphate buffer (pH 7.04) were added hydrazides (300 μM each, in DMSO), aldehydes (100 μM each, in DMSO), aniline (10 mM, in DMSO), the protein Nsp10 (50 μM in phosphate buffer at pH 7.04), and an additional amount of DMSO to reach a final concentration of 5 % in the DCL with 1 mL of end‐volume. The DCL with the protein was allowed to gently mix on a rotating wheel (7 rpm) at room temperature and was frequently monitored via UPLC‐MS and the traces were compared with the blank composition. For analysis, 20 μL of the corresponding library was mixed with 80 μL acetonitrile and 4 μL of NaOH (1 M), the mixture was centrifuged and the supernatant was used for the analysis. Note: The protein‐templated DCLs were run as duplicates.

**DCL‐1**: This experiment library consists of three aldehydes (**A1**–**A3**) and seven hydrazides (**H1**–**H7**). The DCC‐experiment was carried out according to the GP‐1 (blank) and GP‐2 (protein‐templated) in phosphate buffer at pH 7.04 and 5 % DMSO. The DCL composition is detailed in the SI (Supplementary Table 1).

**DCL‐2**: This experiment library consists of three aldehydes **A1**, **A2** and **A4**) and eight hydrazides (**H8**–**H15**).. The DCC‐experiment was carried out according to the GP‐1 (blank) and GP‐2 (protein‐templated) in phosphate buffer at pH 7.04 and 5 % DMSO. The DCL composition is detailed in the SI (Supplementary Table 2).

**General procedure for acylhydrazone formation (GP‐3)**: All acylhydrazones were synthesised and purified according to the procedure described before.^[38]^

**Synthesis and characterisation of acylhydrazones**: (*E*)‐*N*′‐((2‐Aminopyrimidin‐5‐yl)methylene)‐2‐(pyridin‐3‐yl)acetohydrazide (**1**): Compound **1** was synthesised according to GP‐3, starting with aldehyde **A3** (61 mg, 0.496 mmol) and hydrazide **H5** (75 mg, 0.496 mmol) in MeOH (2 mL). The crude was purified by using purification method‐1 (precipitation)^[1]^ to afford **1** as a mixture of *cis* and *trans* conformers of amide (*cis*:*trans*=62 : 38) as a white solid (60.9 mg, 0.237 mmol, 47.9 %).

^1^H NMR (500 MHz, DMSO‐*d*_6_) δ 11.54 (s, 1H, *trans*), 11.35 (s, 1H, *cis*), 8.56 (s, 2H, *cis*), 8.52 (s, 2H, *trans*), 8.51–8.49 (m, 2H, 1 *trans* and 1 *cis*), 8.46 (dd, *J*=4.8, 1.7 Hz, 1H, *trans*), 8.43 (dd, *J*=4.9, 1.7 Hz, 1H, *cis*), 8.05 (s, 1H, *trans*), 7.84 (s, 1H, *cis*), 7.77–7.64 (m, 2H, 1 *trans* and 1 *cis*), 7.42–7.29 (m, 2H, 1 *trans* and 1 *cis*), 7.13 (d, *J*=14.5 Hz, 4H, 2 *trans* and 2 *cis*), 4.00 (s, 2H, *cis*), 3.57 (s, 2H, *trans*). ^13^C NMR (126 MHz, DMSO‐*d*_6_) δ 172.0, 166.2, 164.3, 164.1, 157.5, 157.3, 151.0, 150.5, 148.3, 148.0, 143.7, 139.9, 137.5, 137.1, 132.1, 131.9, 123.9, 123.8, 117.5, 117.4, 38.6, 36.6. HRMS (ESI^+^): m/z calcd. for C_12_H_13_N_6_O^+^ ([M+H]^+^) 257.1145, measured 257.1139.

(*E*)‐*N*′‐((2‐Aminopyrimidin‐5‐yl)methylene)tetrahydrofuran‐3‐carbohydrazide (**2**): Compound **2** was synthesised according to GP‐3, starting with aldehyde **A3** (10.3 mg, 0.084 mmol) and hydrazide **H4** (10.9 mg, 0.084 mmol) in MeOH (1 mL). The crude was purified by using purification method‐1 (precipitation)^[1]^ to afford **2** as a mixture of *cis* and *trans* conformers of amide (*cis*:*trans*=56 : 44) as a white solid (6.6 mg, 0.028 mmol, 33.4 %). ^1^H NMR (500 MHz, DMSO‐*d*_6_) δ 11.35 (s, 1H, trans), 11.25 (s, 1H, cis), 8.51 (s, 4H, 2 *trans* and 2 *cis*), 8.02 (s, 1H, *trans*), 7.82 (s, 1H, *cis*), 7.13 (s, 2H, *trans*), 7.09 (s, 2H, *cis*), 3.98 (t, J=8.2 Hz, 1H, *cis*), 3.90 (t, J=8.2 Hz, 1H, *trans*), 3.84–3.62 (m, 7H, 2 *trans* and 2 *cis*; 1 *trans* and 1 *cis*; 1 *cis*), 2.98 (q, J=7.7 Hz, 1H, 1 *trans*), 2.10–2.00 (m, 4H, 2 *trans* and 2 *cis*). ^13^C NMR (126 MHz, DMSO‐*d*_6_) δ 174.7, 169.4, 164.2, 164.1, 158.7, 157.4, 157.2, 143.3, 139.7, 117.6, 117.5, 70.6, 70.2, 68.3, 68.2, 43.3, 41.1, 30.4, 29.2. HRMS (ESI^+^): m/z calcd. for C_10_H_14_N_5_O_2_^+^ ([M+H]^+^) 236.1142, measured 236.1137.

(*E*)‐2‐(1*H*‐Indol‐3‐yl)‐*N*′‐((2‐methylpyrimidin‐5‐yl)methylene)acetohydrazide (**3**): Compound **3** was synthesised according to GP‐1, starting with aldehyde **A2** (64.6 mg, 0.53 mmol) and hydrazide **H7** (100 mg, 0.53 mmol) in MeOH (2 mL). The crude was purified by using purification method‐1 (precipitation)^[1]^ to afford **3** as a mixture of *cis* and *trans* conformers of amide (*cis*:*trans*=58 : 42) as a white solid (111 mg, 0.38 mmol, 71.4 %). ^1^H NMR (500 MHz, DMSO‐*d*_6_) δ 11.76 (s, 1H, *trans*), 11.52 (s, 1H, *cis*), 10.94 (s, 1H, *trans*), 10.87 (s, 1H, *cis*), 9.00 (s, 2H, *cis*), 8.93 (s, 2H, *trans*), 8.26 (s, 1H, *trans*), 7.98 (s, 1H, *cis*), 7.65–7.55 (m, 2H, 1 *cis* and 1 *trans*), 7.35 (dd, *J*=10.9, 8.1 Hz, 2H, 1 *cis* and 1 *trans*), 7.25 (dd, *J*=10.0, 2.2 Hz, 2H, 1 *cis* and 1 *trans*), 7.13–6.92 (m, 4H, 2 *cis* and 2 *trans*), 4.08 (s, 2H, *cis*), 3.67 (s, 2H, *trans*), 2.66 (s, 3H, *cis*), 2.65 (s, 3H, *trans*). ^13^C NMR (126 MHz, DMSO‐*d*_6_) δ 173.4, 168.3, 168.0, 167.9, 155.5, 155.3, 141.5, 137.7, 136.6, 136.5, 127.8, 127.6, 125.9, 125.9, 124.5, 124.5, 121.5, 121.4, 119.2, 119.1, 118.9, 118.8, 111.9, 111.8, 108.4, 108.4, 32.1, 29.7, 26.1, 26.1. HRMS (ESI^+^): m/z calcd. for C_16_H_16_N_5_O^+^ ([M+H]^+^) 294.1349, measured 294.1340.

(*E*)‐*N*′‐((6‐(Trifluoromethyl)pyridin‐3‐yl)methylene)‐4,5,6,7‐tetrahydrobenzo[*b*]thiophene‐2‐carbohydrazide (**4**): Compound **4** was previously synthesised and characterised.^[38]^

(*E*)‐*N*′‐((2‐Methylpyrimidin‐5‐yl)methylene)‐2‐(pyridin‐3‐yl)acetohydrazide (**5**): Compound **5** was synthesised according to GP‐1, starting with aldehyde **A2** (60.6 mg, 0.496 mmol) and hydrazide **H5** (75 mg, 0.496 mmol) in MeOH (2 mL). The crude was purified by using purification method‐1 (precipitation)^[1]^ to afford **5** as a mixture of *cis* and *trans* conformers of amide (*cis*:*trans*=33 : 67) as a white solid (14.6 mg, 0.057 mmol, 11.5 %).

^1^H NMR (500 MHz, DMSO‐*d*_6_) δ 11.74 (bs, 2H, 1 *cis* and 1 *trans*), 9.01 (s, 2H, *cis*), 8.94 (s, 2H, *trans*), 8.58–8.50 (m, 2H, 1 *cis* and 1 *trans*), 8.47 (dd, *J*=4.8, 1.5 Hz, 1H, *trans*), 8.44 (dd, *J*=4.8, 1.6 Hz, 1H, *cis*), 8.25 (s, 1H, *trans*), 8.01 (s, 1H, *cis*), 7.81–7.63 (m, 2H, 1 *cis* and 1 *trans*), 7.36 (ddd, *J*=12.7, 7.8, 4.8 Hz, 2H, 1 *cis* and 1 *trans*), 4.07 (s, 2H, *cis*), 3.63 (s, 2H, *trans*), 2.66 (s, 3H, *cis*), 2.66 (s, 3H, *trans*).

^13^C NMR (126 MHz, DMSO‐*d*_6_) δ 172.6, 168.4, 168.2, 166.8, 155.6, 155.5, 151.0, 150.6, 148.4, 148.1, 142.2, 138.4, 137.6, 137.2, 131.8, 131.7, 125.8, 123.9, 123.8, 38.5, 36.6, 26.11, 26.08.

HRMS (ESI^+^): m/z calcd. for C_13_H_14_N_5_O^+^ ([M+H]^+^) 256.1193, measured 256.1185.

(*E*)‐2‐(3‐(Pyrazin‐2‐yl)‐1,2,4‐oxadiazol‐5‐yl)‐*N*′‐((6‐(trifluoromethyl)pyridin‐3‐yl)methylene) acetohydrazide (**6**): Compound **6** was synthesised according to GP‐1, starting with aldehyde **A1** (31.8 mg, 0.182 mmol) and hydrazide **H2** (40 mg, 0.182 mmol) in MeOH (1.5 mL). The crude was purified by using purification method‐1 (precipitation)^[1]^ to afford **6** as a mixture of *cis* and *trans* conformers of amide (*cis*:*trans*=70 : 30) as a white solid (26.9 mg, 0.071 mmol, 39.2 %). ^1^H NMR (500 MHz, DMSO‐*d*_6_) δ 12.23 (s, 2H, 1 *cis* and 1 *trans*), 9.28 (d, *J*=1.3 Hz, 1H, *trans*), 9.27 (d, *J*=1.4 Hz, 1H, *cis*), 9.05 (s, 1H, *trans*), 9.03 (s, 1H, *cis*), 8.93–8.85 (m, 4H, 2 *cis* and 2 *trans*), 8.40 (dd, *J*=8.2, 1.6 Hz, 1H, *trans*), 8.38 (s, 1H, *trans*), 8.36 (dd, *J*=8.2, 1.6 Hz, 1H, *cis*), 8.15 (s, 1H, *cis*), 7.99 (d, *J*=8.2 Hz, 1H, *trans*), 7.95 (d, *J*=8.2 Hz, 1H, *cis*), 4.70 (s, 2H, *cis*), 4.32 (s, 2H, *trans*). ^13^C NMR (126 MHz, DMSO‐*d*_6_) δ 176.6, 176.0, 168.1, 166.75, 166.72, 162.1, 149.5, 149.2, 147.7, 147.66, 147.0 (q, *J=*34.1 Hz), 145.78, 145.76, 144.3, 144.2, 144.1, 141.9, 140.5, 136.3, 136.1, 133.7, 133.6, 122.00 (q, *J*=273.9 Hz), 121.5 (q, *J*=2.7 Hz), 121.3 (q, *J*=2.7 Hz), 33.76, 32.82. ^19^F NMR (470 MHz, DMSO‐*d*_6_) δ −66.44, −66.46. HRMS (ESI^+^): m/z calcd. for C_15_H_11_F_3_N_7_O_2_^+^ ([M+H]^+^) 378.0921, measured 378.0911.

(*E*)‐*N*′‐((2‐aminopyrimidin‐5‐yl)methylene)‐4,5,6,7‐tetrahydrobenzo[*b*]thiophene‐2‐carbohydrazide (**7**): Compound **7** was previously synthesised and characterised.^[38]^

(*E*)‐2‐(1*H*‐Indol‐3‐yl)‐N′‐((2‐(trifluoromethyl)pyrimidin‐5‐yl)methylene)acetohydrazide (**8**): Compound **8** was previously synthesised and characterised.^[38]^

(*E*)‐5‐chloro‐*N*′‐((6‐(trifluoromethyl)pyridin‐3‐yl)methylene)thiophene‐2‐carbohydrazide (**9**): Compound **9** was synthesised according to GP‐1, starting with aldehyde **A1** (40.0 mg, 0.228 mmol) and hydrazide **H12** (40.3 mg, 0.228 mmol) in MeOH (1.5 mL). The crude was purified by using purification method‐1 (precipitation)^[1]^ to afford **9** as a mixture of *cis* and *trans* conformers of amide (*cis*:*trans*=75 : 25) as a white solid (45.0 mg, 0.135 mmol, 59.0 %). ^1^H NMR (500 MHz, DMSO‐*d*_6_) δ 12.34 (br s, 2H, 1 *cis* and 1 *trans*), 9.12 (br s, 1H, *cis*), 9.03–9.08 (m, 1H, *trans*), 8.54 (br s, 1H, *trans*), 8.43 (br d, *J=*7.6 Hz, 2H, 1 *cis* and 1 *trans*), 8.24 (br s, 1H, *cis*), 8.07 (br d, *J=*7.9 Hz, 1H, *cis*), 8.01 (br s, 1H, *trans*), 7.88–7.95 (m, 1H, *cis*), 7.84 (br s, 1H, *trans*), 7.30 (d, *J=*4.1 Hz, 2H, 1 *cis* and 1 *trans*). ^13^C NMR (126 MHz, DMSO‐*d*_6_) δ 160.4, 148.9, 146.7, 140.6, 137.6, 136.1, 135.7, 134.9, 133.1, 130.1, 128.4, 128.4, 126.7, 122.6, 121.2, 120.5. ^19^F NMR (470 MHz, DMSO‐*d*_6_) δ −66.47. HRMS (ESI^+^): m/z calcd. for C_12_H_8_ClF_3_N_3_OS^+^ ([M+H]^+^) 334.0023, measured 334.0013.

(*E*)‐*N*′‐((2‐methylpyrimidin‐5‐yl)methylene)‐1,4‐dioxane‐2‐carbohydrazide (**10**): Compound **10** was synthesised according to GP‐1, starting with aldehyde **A2** (40.0 mg, 0.328 mmol) and hydrazide **H8** (47.9 mg, 0.328 mmol) in MeOH (1.5 mL). The crude was purified by using purification method‐1 (precipitation)^[1]^ to afford **10** as a mixture of *cis* and *trans* conformers of amide (*cis*:*trans*=80 : 20) as a yellow solid (32.0 mg, 0.127 mmol, 39.0 %). ^1^H NMR (500 MHz, DMSO‐*d*_6_) δ 11.62 (br s, 2H, 1 *cis* and 1 *trans*), 8.94 (s, 2H, *trans*), 8.92 (s, 2H, *cis*), 8.42 (s, 1H, *cis*), 7.98 (s, 1H, *trans*), 4.93 (dd, *J=*8.7, 2.9 Hz, 1H, *trans*), 4.23 (dd, *J=*9.2, 3.1 Hz, 1H, *cis*), 3.83–3.95 (m, 4H, 2 *cis* and 2 *trans*), 3.66–3.73 (m, 4H, 2 *cis* and 2 *trans*), 3.50–3.59 (m, 4H, 2 *cis* and 2 *trans*), 2.64–2.67 (m, 6H, 3 *cis* and 3 *trans*). ^13^C NMR (126 MHz, DMSO‐*d*_6_) δ 168.1, 167.8, 164.8, 155.2, 155.0, 143.4, 138.7, 125.3, 125.2, 74.2, 72.1, 67.4, 67.0, 65.9, 65.7, 65.6, 65.2, 25.7, 25.6. HRMS (ESI^+^): m/z calcd. for C_11_H_15_N_4_O_3_ ([M+H]^+^) 251.1139, measured 251.1130.

(*E*)‐*N′*‐((6‐(trifluoromethyl)pyridin‐3‐yl)methylene)thiophene‐2‐carbohydrazide (**11**): Compound **11** was synthesised according to GP‐1, starting with aldehyde **A1** (40.0 mg, 0.228 mmol) and hydrazide **H14** (32.5 mg, 0.228 mmol) in MeOH (1.5 mL). The crude was purified by using purification method‐1 (precipitation)^[1]^ to afford **11** as a mixture of *cis* and *trans* conformers of amide (*cis*:*trans*=55 : 45) as a white solid (34.2 mg, 0.114 mmol, 50.0 %). ^1^H NMR (500 MHz, DMSO‐*d*_6_) δ 12.22 (br s, 2 H, 1 *cis* and 1 *trans*), 9.14 (br s, 1 H, *cis*), 9.06 (br s, 1 H, *trans*), 8.56 (br s, 1 H *trans*), 8.43 (br s, 2 H, 1 *cis* and 1 *trans*), 8.23 (br s, 1 H, *cis*), 8.08 (br s, 1 H, *cis*), 7.89–8.04 (m, 3 H, 1 *cis* and 2 *trans*), 7.25 (t, *J=*4.3 Hz, 2 H, 1 *cis* and 1 *trans*). ^13^C NMR (126 MHz, DMSO‐*d*_6_) δ 148.8, 143.2, 143.1, 139.8, 139.8, 135.9, 135.6, 135.2, 135.2, 133.5, 132.5, 129.5, 128.3, 126.9, 121.1, 121.6. ^19^F NMR (470 MHz, DMSO‐*d*_6_) δ −66.43. HRMS (ESI^+^): m/z calcd. for C_12_H_9_F_3_N_3_OS^+^ ([M+H]^+^) 300.0413, measured 300.0405.

(*E*)‐*N*′‐(2‐fluoro‐4‐hydroxybenzylidene)‐4‐methylbenzenesulfonohydrazide (**12**): Compound **12** was synthesised according to GP‐1, starting with aldehyde **A4** (40.0 mg, 0.285 mmol) and hydrazide **H15** (53.2 mg, 0.285 mmol) in MeOH (1.5 mL). The crude was purified by using purification method‐1 (precipitation)^[1]^ to afford **12** as the pure *cis* conformer of amide as a pale yellow solid (31.5 mg, 0.102 mmol, 35.8 %). ^1^H NMR (500 MHz, DMSO‐*d*_6_) δ 11.28 (br s, 1 H), 10.43 (br s, 1 H), 7.96 (s, 1 H), 7.73 (m, *J=*8.2 Hz, 2 H), 7.51 (t, *J=*8.7 Hz, 1 H), 7.40 (m, *J=*8.2 Hz, 2 H), 6.64 (dd, *J=*8.6, 2.2 Hz, 1 H), 6.56 (dd, *J=*12.7, 2.3 Hz, 1 H), 2.36 (s, 3 H). ^13^C NMR (126 MHz, DMSO‐*d*_6_) δ 162.5, 161.0, 160.9, 160.5, 143.5, 140.4, 140.4, 136.1, 129.7, 127.2, 127.2, 127.1, 112.8, 112.8, 112.2, 112.1, 102.6, 102.4, 21.0. ^19^F NMR (470 MHz, DMSO‐*d*_6_) δ −119.56. HRMS (ESI^+^): m/z calcd. for C_14_H_14_FN_2_O_3_S^+^ ([M+H]^+^) 309.0704, measured 309.0695.

(*E*)‐*N*′‐((2‐methylpyrimidin‐5‐yl)methylene)thiophene‐2‐carbohydrazide (**13**): Compound **13** was synthesised according to GP‐1, starting with aldehyde **A2** (40.0 mg, 0.328 mmol) and hydrazide **H14** (46.6 mg, 0.328 mmol) in MeOH (1.5 mL). The crude was purified by using purification method‐1 (precipitation)^[1]^ to afford **13** as a mixture of *cis* and *trans* conformers of amide (*cis*:*trans*=60 : 40) as a yellow solid (34.7 mg, 0.141 mmol, 43.0 %). ^1^H NMR (500 MHz, DMSO‐*d*_6_) δ 12.10 (s, 2 H, 1 *cis* and 1 *trans*), 9.06 (br s, 2 H, *cis*), 8.99 (br s, 2 H, *trans*), 8.45 (br s, 1 H, *trans*), 8.11 (br s, 1 H, *cis*), 8.06 (br s, 1 H, *cis*), 7.89–8.01 (m, 3 H, 1 *cis* and 2 *trans*), 7.23 (br t, *J=*4.2 Hz, 2 H, 1 *cis* and 1 *trans*), 2.67 (s, 6 H, 3 *cis* and 3 *trans*). ^13^C NMR (126 MHz, DMSO‐*d*_6_) δ 155.2, 142.4, 138.7, 135.2, 135.1, 132.3, 129.3, 129.3, 128.2, 126.8, 125.4, 25.7. HRMS (ESI^+^): m/z calcd. for C_11_H_11_N_4_OS^+^ ([M+H]^+^) 247.0648, measured 247.0640.

(*E*)‐*N*′‐(2‐fluoro‐4‐hydroxybenzylidene)‐1,4‐dioxane‐2‐carbohydrazide (**14**): Compound **14** was synthesised according to GP‐1, starting with aldehyde **A4** (40.0 mg, 0.285 mmol) and hydrazide **H8** (41.7 mg, 0.285 mmol) in MeOH (1.5 mL). The crude was purified by using purification method‐1 (precipitation)^[1]^ to afford **14** as a mixture of *cis* and *trans* conformers of amide (*cis*:*trans*=80 : 20) as a white solid (60.0 mg, 0.224 mmol, 78.4 %). ^1^H NMR (500 MHz, DMSO‐*d*_6_) δ 11.36 (s, 1 H, *trans*), 11.28 (s, 1 H, *cis*), 10.43 (br s, 2 H, 1 *cis* and 1 *trans*), 8.53 (s, 1 H, *cis*), 8.07 (s, 1 H, *trans*), 7.69 (t, *J=*8.7 Hz, 2 H, 1 *cis* and 1 *trans*), 6.65–6.73 (m, 2 H, 1 *cis* and 1 *trans*), 6.60 (dd, *J=*12.6, 1.8 Hz, 2 H, 1 *cis* and 1 *trans*), 4.86 (dd, *J=*8.9, 2.4 Hz, 1 H, *trans*), 4.18 (dd, *J=*9.3, 2.9 Hz, 1 H, *cis*), 3.81–3.95 (m, 4 H, 2 *cis* and 2 *trans*), 3.63–3.72 (m, 4 H, 2 *cis* and 2 *trans*), 3.47–3.59 (m, 4 H, 2 *cis* and 2 *trans*). ^13^C NMR (126 MHz, DMSO‐*d*_6_) δ 169.0, 164.3, 162.9, 161.1, 161.0, 160.9, 141.8, 141.7, 137.4, 137.4, 127.4, 127.3, 127.3, 127.2, 112.8, 112.8, 112.6, 112.6, 102.7, 102.6, 102.5, 102.4, 74.3, 72.4, 67.5, 66.9, 65.8, 65.7, 65.6, 65.3. ^19^F NMR (470 MHz, DMSO‐*d*_6_) δ −119.33 (br dd, *J*=13.0, 9.5 Hz, 1F), −119.44 (br dd, *J*=13.9, 8.7 Hz, 1F). HRMS (ESI^+^): m/z calcd. for C_12_H_14_FN_2_O_4_^+^ ([M+H]^+^) 269.0932, measured 269.0924.

(*E*)‐*N*′‐((2‐methylpyrimidin‐5‐yl)methylene)‐2‐(4‐nitrophenoxy)acetohydrazide (**15**): Compound **15** was synthesised according to GP‐1, starting with aldehyde **A2** (40.0 mg, 0.328 mmol) and hydrazide **H13** (69.2 mg, 0.328 mmol) in MeOH (1.5 mL). The crude was purified by using purification method‐1 (precipitation)^[1]^ to afford **15** as a mixture of *cis* and *trans* conformers of amide (*cis*:*trans*=75 : 25) as a pale yellow solid (62.5 mg, 0.198 mmol, 60.5 %). ^1^H NMR (500 MHz, DMSO‐*d*_6_) δ 11.92 (br s, 2 H, 1 *cis* and 1 *trans*), 9.03 (s, 2 H, *cis*), 8.96 (s, 1 H, *trans*), 8.33 (s, 1 H, *trans*), 8.24 (br d, *J=*9.6 Hz, 2 H, *trans*), 8.20 (d, *J=*9.2 Hz, 2 H, *cis*), 8.02 (s, 1 H, *cis*), 7.20 (br d, *J=*9.5 Hz,, 2 H, *trans*), 7.17 (d, *J=*9.3 Hz, 2 H, *cis*), 5.39 (s, 2 H, *cis*), 4.89 (s, 2 H, *trans*), 2.65 (s, 6 H, 3 *cis* and 3 *trans*). ^13^C NMR (126 MHz, DMSO‐*d*_6_) δ 168.5, 168.1, 167.9, 163.7, 163.6, 163.0, 155.2, 143.0, 141.3, 141.0, 138.8, 125.9, 125.7, 125.2, 125.1, 115.3, 115.2, 66.6, 65.5, 25.7, 25.6. HRMS (ESI^+^): m/z calcd. for C_14_H_13_N_5_O_4_^+^ ([M+H]^+^) 316.1040, measured 316.1031.

(*E*)‐2‐(1,1‐dioxidotetrahydrothiophen‐3‐yl)‐*N*′‐((6‐(trifluoromethyl)pyridin‐3‐yl)methylene)acetohydrazide (**16**): Compound **16** was synthesised according to GP‐1, starting with aldehyde **A1** (40.0 mg, 0.228 mmol) and hydrazide **H10** (43.9 mg, 0.228 mmol) in MeOH (1.5 mL). The crude was purified by using purification method‐1 (precipitation)^[1]^ to afford **16** as a mixture of *cis* and *trans* conformers of amide (*cis*:*trans*=65 : 35) as a pale yellow solid (54.7 mg, 0.157 mmol, 68.5 %). ^1^H NMR (500 MHz, DMSO‐*d*_6_) δ 11.75 (br s, 2 H, 1 *cis* and 1 *trans*), 9.04 (s, 1 H, *cis*), 9.01 (s, 1 H, *trans*), 8.35 (dd, *J=*8.2, 1.4 Hz, 2 H, 1 *cis* and 1 *trans*), 8.29 (s, 1 H, *trans*), 8.09 (s, 1 H, *cis*), 7.93–7.98 (m, 2 H, 1 *cis* and 1 *trans*), 3.17–3.31 (m, 6 H, 3 *cis* and 3 *trans*), 3.03–3.12 (m, 2 H, 1 *cis* and 1 *trans*), 2.83–2.98 (m, 2 H, 1 *cis* and 1 *trans*), 2.82–2.89 (m, 2 H, 1 *cis* and 1 *trans*), 2.72–2.81 (m, 2 H, 1 *cis* and 1 *trans*), 2.26–2.37 (m, 2 H, 1 *cis* and 1 *trans*), 1.78–1.91 (m, 2 H, 1 *cis* and 1 *trans*). ^13^C NMR (126 MHz, DMSO‐*d*_6_) δ 172.9, 167.1, 148.8, 148.6, 142.0, 138.7, 135.6, 135.4, 133.5, 121.0, 120.9, 120.9, 55.9, 55.6, 51.4, 51.2, 38.1, 36.1, 32.6, 32.2, 28.2, 28.1. ^19^F NMR (470 MHz, DMSO‐*d*_6_) δ −66.40. HRMS (ESI^+^): m/z calcd. for C_13_H_15_F_3_N_3_O_3_S^+^ ([M+H]^+^) 350.0781, measured 350.0771.

**Nsp10 expression and purification**: Synthesised Nsp10 sequence: SENLYFQGAGNATEVPANSTVLSFCAFAVDAAKAYKDYLASGGQPITNCVKMLCTHTGTGQAITVTPEANMDQESFGGASCCLYCRCHIDHPNPKGFCDLKGKYVQIPTTCANDPVGFTLKNTVCTVCGMWKGYGCSCDQLREPMLQ. After TEV cleavage (purified sequence):

GAGNATEVPANSTVLSFCAFAVDAAKAYKDYLASGGQPITNCVKMLCTHTGTGQAITVTPEANMDQESFGGASCCLYCRCHIDHPNPKGFCDLKGKYVQIPTTCANDPVGFTLKNTVCTVCGMWKGYGCSCDQLREPMLQ. The gene construct was synthesised commercially, cloned into MCS1 of a modified version of pRSF‐Duet‐1 carrying an ampicillin resistance gene (called pRSF‐Duet‐1‐Amp) and transformed into Lemo21(DE3) for overexpression. The transformed cells were grown in a selective LB medium at 37 °C supplemented with Carbenicillin (100 mg/mL) and chloramphenicol (34 mg/mL) up to an OD600 of 0.9, then induced with 0.1 mm isopropyl‐l‐d‐thiogalactoside (IPTG) and were subsequently grown at 18 °C for 16 h. The harvested cells were then disrupted in a microfluidizer after resuspension in a washing buffer consisting of 20 mm Tris pH 8.0, 500 mm NaCl, 20 mm Imidazole pH 8.0 (100 mL buffer per 25 g of wet cell pellet). After centrifugation, the supernatant was loaded onto a 5 mL HisTrap HP column equilibrated in washing buffer. After an extensive washing step with 30 column volumes of washing buffer, the bound fraction was then eluted with an elution buffer consisting of 20 mm Tris pH 8.0, 500 mm NaCl, 250 mm Imidazole pH 8.0. Eluted protein was passed over a Hiprep 26/10 Desalting column equilibrated in washing buffer to remove excess imidazole, after which TEV digestion was carried out by adding TEV protease in a 1 : 10 protein: TEV ratio and incubating the solution at 4 °C for 16 h. The *N*‐terminal 6x His‐Tag was removed by passing the protein over a 5 mL HisTrap HP column and collection of the flow‐through. The protein was concentrated using a centrifuge filter with a 10 kDa cutoff. Purification of Nsp10 was finalised by size exclusion chromatography using a HiLoad 16/600 Superdex 200 pg column equilibrated in 20 mm HEPES pH 7.0. Sodium dodecyl sulfate‐polyacrylamide gel electrophoresis (SDS‐PAGE) and protein mass spectrometry were used to examine the purity of protein purifications.

**Nsp10 stability analysis in the DCC conditions via thermal shift assay**: Prior to DCC experiments, the stability of Nsp10 in the DCC buffer conditions was monitored for 2 days by measuring its melting temperature (T_m_) using thermal shift assay (TSA). Furthermore, the stability of Nsp10 was tested in phosphate buffer at pH 7.04 in the presence and absence of DMSO (5 %). Nsp10 was incubated for 48 h at a final concentration of 1 mg/mL in phosphate buffer at pH 7.04 with/without 5 % DMSO. Samples were collected at t=0, 24 & 48 hours. The final concentration of the Nsp10 and the SYPRO orange 5000x dye (Sigma‐Aldrich) in the TSA were 0.1 mg/mL and 50x, respectively. The experiments were performed in a 96‐well PCR plate (Thermoscientific). The final volume per well was 25 μL, consisting of 20 μL of phosphate buffer 100 mM, 2.5 μL of 1 mg/mLNsp10 in phosphate buffer 100 mM, pH: 7.04, with/without 5 % DMSO and 2.5 μL of 500 x SYPRO orange dye. The plate was centrifuged for 1 min at room temperature at 1200 rpm.The melting temperature of the protein was measured using a Real‐time PCR machine (Step one plus, Applied Biosystem). The conditions of the experiment were adjusted using Step One 2.3 software. The starting temperature, the ending temperature and the heating rate were set as 21 °C, 95 °C and 0.5 °C/min, respectively. The melting curves were analysed using Protein Thermal Shift 1.3 software. T_m_ of the Nsp10 under these conditions was found 49.9 °C. The results of the TSA are available in the SI (Supplementary Table 3).

**Native Mass Spectrometry**: Purified Nsp10 was buffer‐exchanged into a volatile 10 mm ammonium acetate solution (pH 7.0) by repeated ultracentrifugation using Amicon ultra 10 K centrifugal filters (Merck KGaA, Darmstadt, Germany) in a Hitachi table centrifuge (Hitachi Koki Co., Tokyo, Japan) with a 40° fixed angle rotor at 14,000 x g and 4 °C for 10 min per run. The protein was diluted to 2.5 μM to obtain the highest sensitivity in the mass spectrometer. For compound screening, 1 μL of each compound at 1 mM in DMSO was added to 100 μL of the protein solution resulting in a final compound concentration of 10 μm and a compound/protein ratio of 4 : 1. Native MS analysis was conducted after incubation for 1 h at room temperature. Experiments were performed on a Bruker SolariX XR 7T Fourier‐transform ion cyclotron resonance (FT‐ICR) mass spectrometer equipped with a high‐resolution electrospray ionization (HRESI) source (Bruker Daltonics, Billerica, MA, USA) by direct infusion of the mixture at a flow rate of 2 μL/min. Source parameters were set to 500 V end‐plate offset, 4000 V capillary voltage, 3 bar nebulizer gas pressure, 5 L/min dry gas and 200 °C dry gas temperature. The instrument was calibrated using an Agilent ESI−L low concentration tuning mix (G1969‐85000). Profile spectra were recorded in positive ion mode with a mass range from 150 to 5000 *m/z*. Each spectrum was a sum of 128 transients composed of 512k data points. The pulse sequence control and data acquisition were controlled by ftmscontrol software in a Windows operating system. When a noncovalent complex was detected, the molecular weight of the binding compound was confirmed by calculating its mass using the following equation: MW_compound_=Δ*m/z* × *z*.

**Binding affinity (KD) determinations by surface plasmon resonance (SPR)**: The SPR binding studies were performed using a Reichert SR7500DC surface plasmon resonance spectrometer (Reichert Technologies, Depew, NY, USA), and medium density carboxymethyl dextran hydrogel CMD500 M sensor chips (XanTec Bioanalytics, Düsseldorf, Germany). Milli‐Q water was used as the running buffer for immobilization. Nsp10 was immobilised in one of the two flow cells according to reported amine‐coupling protocols.^[50]^ The other flow cell was left blank to serve as a reference. The system was initially primed with borate buffer 100 mM (pH 9.0), then the carboxymethyldextran matrix was activated by a 1 : 1 mixture of *N*‐ethyl‐*N*′‐(3‐dimethylaminopropyl)carbodiimide hydrochloride (EDC) 100 mM and *N*‐hydroxysuccinimide (NHS) 100 mM at a flow rate of 10 μL/min for 7 min. The Nsp10 was diluted to a final concentration of 24 μM in 10 mM sodium acetate buffer (pH 4.5) and was injected at a flow rate of 5 μL/min for 10 min. The non‐reacted surface was quenched by 1 M ethanolamine hydrochloride (pH 8.5) at a flow rate of 25 μL/min for 3 min. A series of 10 buffer injections was run initially on both reference and active surfaces to equilibrate the system resulting in a stable immobilisation level of approximately 5000 μ refractive index unit (μRIU). Phosphate buffered saline (PBS) buffer (10 mM Na_2_HPO_4_, 1.8 mM KH_2_PO_4_, 137 mM NaCl, 2.7 mM KCl, 0.05 % v/v Tween 20, pH 7.4) containing 5 % v/v DMSO was used as the running buffer. All running buffers were filtered and degassed prior to use. Binding experiments were performed at 20 °C. Compounds dissolved in DMSO were diluted with the running buffer (final DMSO concentration of 5 % v/v) and were injected at a flow rate of 30 μL/min. Single‐cycle kinetics were applied for *K*_D_ determination. The association time was set to 60 s, and the dissociation phase was recorded for 120 sec. Ethylene glycol 80 % in the running buffer was used for the regeneration of the surface. Differences in the bulk refractive index due to DMSO were corrected by a calibration curve (nine concentrations: 3–7 % v/v DMSO in the running buffer). Data processing and analysis were performed by Scrubber software (Version 2.0c, 2008, BioLogic Software). Sensorgrams were calculated by sequential subtractions of the corresponding curves obtained from the reference flow cell and the running buffer (blank). SPR responses are expressed in the resonance unit (RU). The *K*_D_ values were calculated by the fitting of the steady‐state binding responses to a 1 : 1 Langmuir interaction model.

**Plasmid construction**: Nsp10 gene was amplified from pET15b Nsp10^[14]^ and cloned into the *Nde*I‐*Nco*I cloning sites of a pET28b commercial expression vector to generate pET28b Nsp10, which expresses a C‐terminal His‐tagged version of Nsp10. Plasmid expressing an *N*‐terminal His‐tagged version of Nsp14 was already described.^[14]^ The full‐length Nsp16 gene from SARS‐CoV‐2 (Uniprot ID P0DTD1) was optimised for *E. coli* expression and synthesised by GenScript (USA). The synthesised gene was subsequently cloned into the *Nde*I–*Bam*HI sites of commercial pET15b to generate pET15b Nsp16, which expresses an *N*‐terminal His‐tagged version of Nsp16.

**Protein expression and purification for enzymatic assays**: Plasmid expressing Nsp10 was transformed into BL21(DE3) cells, while plasmids expressing Nsp14 and Nsp16 were transformed into Rosetta cells for the expression of the recombinant proteins. Cells were grown in LB medium supplemented with 150 μg/mL ampicillin (Nsp10 variants) and 50 μg/mL chloramphenicol (Nsp16) or in TB medium supplemented with 150 μg/mL ampicillin and 50 μg/mL chloramphenicol (Nsp14) at 30 °C to an OD_600_ of 0.5. Protein expression was induced by the addition of 0.5 mm IPTG and further incubation for 16 h at 16 °C (Nsp10 and Nsp14) or 4 h at 30 °C (Nsp16). The cells were pelleted by centrifugation and stored at −80 °C. The thawed cell pellets were resuspended in buffer A (40 mm Tris‐HCl pH 8.0, 150 mm NaCl, 10 mM Imidazole) supplemented with 1 mm of Phenylmethylsulfonyl fluoride (PMSF). Cell suspensions were lysed using the FastPrep‐24 (MP Biomedical, USA) for 60 seconds at 6.5 m/s. The raw extract was treated with benzonase (Sigma) and clarified by a 30 min centrifugation at 10,000 x g. Protein purification proceeded as described in Saramago *et al*, 2021.^[14]^ Briefly, the cleared lysates were subjected to histidine‐affinity chromatography using a HisTrap HP column (Cytiva, USA), followed by a size‐exclusion chromatography using a Superdex 200 Increase 10/300GL column (Cytiva, USA). All proteins were purified at least twice to ensure the reproducibility of the results. Proteins were quantified using the Bradford Method, and 50 % (v/v) glycerol was added to the final fractions prior to storage at −20 °C.

**RNase Activity Assays**: To test the ExoN activity of SARS‐CoV‐2 Nsp10/Nsp14 complex, a synthetic 22‐mer oligoribonucleotide (H4 5’‐UGACGGCCCGGAAAACCGGGCC‐3’) (StabVida, Portugal) was used as a substrate. The RNA was labelled at its 5′ ends with [^32^P]‐γ‐ATP and T4 Polynucleotide Kinase (Thermo Scientific) in a standard reaction, as described in Saramago *et al* 2021.^[14]^ The synthetic RNA was resuspended in 10 mm of Tris‐HCl pH 8.0 and incubated for 10 min at 80 °C followed by 45 min at 37 °C.

To perform the assays, different concentrations of the compounds (as indicated in the respective figures), solubilised in DMSO, were first incubated with 2,000 nm of Nsp10 protein (7 minutes at RT). The assays were performed in a final volume of 12 μl containing the activity buffer (50 mm HEPES pH 7.4, 1 mm DTT, 5 mM MgCl_2_), 50 nm of P^32^‐synthetic RNA substrate, and the compound‐Nsp10 mixture. The reaction was started through the addition of 500 nm of Nsp14, and incubated at 37 °C for 30 min. The reactions were stopped by adding a gel‐loading buffer containing 80 % (v/v) formamide and 10 mm EDTA. To ensure the accuracy of the results, the amount of DMSO in each assay was kept constant. A reaction control was performed in the absence of any of the enzymes, using the same conditions. Reaction products were resolved in a denaturant 7 M urea/20 % polyacrylamide gel. Signals were visualised by PhosphorImaging (TLA‐5100 Series, Fuji). All the experiments were performed at least in triplicate using proteins purified on two distinct occasions. Inhibition profile curves were calculated by quantification of full‐length RNA substrate using ImageQuant software. Data were plotted against log10 of [compound] and dose‐response curves were generated using non‐linear regression (GraphPad Prism 8 software).

**Preparation of the Capped RNA Substrate**: A 30‐mer RNA substrate (CCCGACACCAACCACUAAAAAAAAAAAAAA) was transcribed *in vitro* using a synthetic DNA template (5’‐TTTTTTTTTTTTTTAGTGGTTGGTGTCGGGCTATAGTGAGTCGTATTA‐3’) and a T7 promoter oligonucleotide (5’‐TAATACGACTCACTATAG‐3’) obtained from StabVida, Portugal, using the method described by Milligan *et al*.^[51]^ Briefly, the synthetic DNA template (0.5 μm) and the T7 promoter oligonucleotide (0.6 μM) were annealed in 10 mM of Tris‐HCl pH 8.0 by heating for 5 min at 70 °C, followed by incubation at 37 °C for 30 min. *In vitro* transcription was carried out using the NZY T7 High Yield RNA Synthesis kit (NZYTech, Portugal) following the manufacturer's instructions. To remove the DNA template, 1 U of DNase (Invitrogen) was added to the reaction and incubated at 37 °C for 15 min. To insert the cap structure in the 5’‐end of the 30‐mer RNA substrate and create the cap0 RNA (m7Gppp‐RNA) used as a substrate for the MTase activity of Nsp16, we used the vaccinia virus capping enzyme following the manufacturer's protocol (New England Biolabs Inc., USA). The capping reaction was performed in the presence of 0.1 mm of SAM, which is the methyl donor, and 1 mm of GTP. The m7Gppp‐RNA substrate was stored at −20 °C.

**MTase Activity Assays**: To test the MTase activity of SARS‐CoV‐2 Nsp10/Nsp16 complex, a reaction mix containing 400 nm of Nsp10, 5 μM of SAM, 300 nm of the cap0 (m7Gppp)‐RNA substrate was prepared in the reaction buffer (20 mm Tris‐HCl pH 8.0, 1 mm DTT and 20 mm MnCl_2_). The compounds, solubilised in DMSO and at different concentrations (as indicated in the respective figure), were previously incubated with nsp10 for 7 min at RT, before the reactions were started through the addition of 100 nm of Nsp16, and incubated for 1 h at 37 °C. A reaction control was performed in the absence of Nsp10 and Nsp16. The DMSO concentration was kept constant in all the reactions performed to ensure the precision of the results. The MTase activity was measured using the MTase‐Glo Methyltransferase bioluminescence assay kit (Promega, USA) according to the manufacturer's protocol. Luminescence was measured using a FLUOstar OPTIMA microplate reader (BMG Labtech, Germany) in arbitrary units and normalised assigning 100 % to the activity in the absence of any chemical compound. The data from at least 3 independent experiments using proteins purified on two distinct occasions were plotted against log10 of [compound], and dose‐response curves were generated using non‐linear regression (GraphPad Prism v8.3.0 software).

**hCoV‐229E‐luc antiviral assay**: Hepatoma cells expressing firefly luciferase (Huh‐7.5 FLuc) were seeded in 96‐well plates at 2 x 10^4^ cells/well in Dulbecco's modified Eagle medium (DMEM) containing 10 % fetal calf serum (FCS), 2 mM L‐glutamine, 100 μg/mL streptomycin, 100 U/mL penicillin and 1 % non‐essential amino acids. The next day, the cells were inoculated in duplicates with the *renilla* luciferase expressing HCoV‐229E‐luc virus in presence of the compounds in indicated concentrations or DMSO and incubated at 33 °C (5 % CO_2_). After the incubation period of 48 h cells were lysed with 50 μL 0.5 % Triton X‐100 in PBS and frozen at −20 °C. Fluc and RLuc activity were measured in 20 μL of cell lysate each, representing residual cell viability and 229E‐infectivity, respectively (Berthold Centro plate luminometer version 2.02). Mean values were calculated from three biological replicates and normalised to solvent control. Non‐linear regression curves were performed with GraphPad Prism 9 which allowed for the calculation of half‐maximal inhibitory (IC_50_) and cytotoxic concentration (CC_50_).

**SARS‐CoV‐2 antiviral assay**: Calu‐3 cells (a human lung cancer cell line) were seeded in 96‐well plates at 5 x 10^4^ cells/well in Dulbecco's modified Eagle medium (DMEM) containing 10 % fetal calf serum (FCS), 2 mm l‐glutamine, 100 μg/mL streptomycin, 100 U/mL penicillin and 1 % non‐essential amino acids. After 48 h the cells were inoculated in duplicates with the SARS‐CoV‐2 virus in presence of the compounds in indicated concentrations or DMSO and incubated at 37 °C (5 % CO_2_). After 48 h post‐infection the supernatants were lysed and heat‐inactivated at 70 °C for 15 min. The RNA extraction of the supernatants was performed with the Maxwell® 16 Viral Total Nucleic Acid Purification Kit (Promega). The qRT‐PCR (LightCycler® 480; Roche) was performed with the LightMix® Modular SARS‐CoV‐2 (COVID19) RdRP Kit (Roche), which detects a conserved region of the RdRP gene. Mean values were calculated from two biological replicates and normalized to solvent virus control.

## Associated Content

**Supporting Information**. The following files are available free of charge.

Supplementary tables (tdDCC composition and TSA results), ^1^H and ^13^C NMR spectra, HRMS spectra and representative HPLC traces of compounds **1**–**16**, native MS spectra and SPR sensograms (PDF).

Molecular formula string (CSV).

## Abbrevitations Used

TdDCC (target‐directed Dynamic Combinatorial Chemistry), DCL (Dynamic Combinatorial Library), Nsp (Non‐structural protein), ExoN (Exoribonuclease), MTase (Methyltransferase), PPI (protein‐protein interaction).

## Author Contributions

R.P.J. and G.J. contributed equally to this work, they designed and performed the tdDCC experiments and synthesised the hits. S.A., M.V.C. and R.G.M. expressed and purified the Nsp10, Nsp14 and Nsp16 enzymes. M.S. and M.V.C. performed the enzyme activity assays. N.F. and C.D.B performed the near‐native MS assays. R.P.J. and G.J. performed the SPR assays. S. J. and S.B. performed the protein stability assays by TSA. A.P.G. and N.M.K performed the antiviral assays. T.P. coordinated the tests for antiviral activity and carried out the evaluation. R.M coordinated the near‐native MS assays. C.M.A. coordinated the enzymatic assays. A.K.H.H., R.P.J. and G.J. conceived the study and A. K. H. H. supervised the research. All authors contributed to the writing and editing of the manuscript

## Conflict of Interests

There are no conflicts to declare.

1

## Supporting information

As a service to our authors and readers, this journal provides supporting information supplied by the authors. Such materials are peer reviewed and may be re‐organized for online delivery, but are not copy‐edited or typeset. Technical support issues arising from supporting information (other than missing files) should be addressed to the authors.

Supporting Information

## Data Availability

The data that support the findings of this study are available in the supplementary material of this article.
