# Supplementary material for: Dynamic Combinatorial Chemistry Unveils Nsp10 Inhibitors with Antiviral Potential Against SARS‐CoV‐2
Source: Chemistry. 2024 Dec 23;31(4):e202403390. doi: 10.1002/chem.202403390 (PMC11739841; doi:10.1002/chem.202403390)
Supplement: Supplementary file 1 — Supporting Information [file CHEM-31-e202403390-s001.pdf]

Chemistry–A European Journal

Supporting Information

Dynamic Combinatorial Chemistry Unveils Nsp10 Inhibitors with Antiviral Potential Against SARS-CoV-2

Ravindra P. Jumde, Gwenaëlle Jézéquel, Margarida Saramago, Nicolas Frank, Sebastian Adam, Marta V. Cunha, Chantal D. Bader, Antonia P. Gunesch, Natalie M. Köhler, Sandra Johannsen, Spyridon Bousis, Thomas Pietschmann, Rute G. Matos, Rolf Müller, Cecília M. Arraiano, and Anna K. H. Hirsch*

Dynamic Combinatorial Chemistry Unveils Nsp10 Inhibitors with Antiviral Potential Against SARS-CoV-2

Ravindra P. Jumde [1,2#], Gwenaëlle Jézéquel [1#], Margarida Saramago [3], Nicolas Frank [1,4], Sebastian Adam [1], Marta V. Cunha [3], Chantal D. Bader [1], Antonia P. Gunesch [5], Natalie M. Köhler [5], Sandra Johannsen [1,4], Spyridon Bousis[1,4], Thomas Pietschmann [5,6,7], Rute G. Matos [3], Rolf Müller [1,4,6,7], Cecília M. Arraiano [3], Anna K. H. Hirsch*[1,4,6,7]

[1] Helmholtz Institute for Pharmaceutical Research Saarland (HIPS) – Helmholtz Centre for Infection Research (HZI), Campus E 8.1, 66123 Saarbrücken, Germany

[2] Current address: Global Antibiotic Research & Development Partnership (GARDP), Chemin Camille-Vidart 15, 1202 Geneva, Switzerland

[3] Instituto de Tecnologia Química e Biológica António Xavier, Universidade Nova de Lisboa, Avenida da República, 2780-157 Oeiras, Portugal

[4] Saarland University, Department of Pharmacy, Campus E 8.1, 66123 Saarbrücken, Germany

[5] Institute for Experimental Virology, Twincore - Centre for Experimental and Clinical Infection Research; Feodor-Lynen-Str. 7, 30625 Hannover, Germany.

[6] Cluster of Excellence RESIST (EXC 2155), Hannover Medical School; 30625 Hannover, Germany

[7] Helmholtz International Lab for Anti-infectives, Campus E 8.1, 66123 Saarbrücken, Germany

[#] These authors contributed equally to this work.

*Corresponding author: A.K.H. Hirsch anna.hirsch@helmholtz-hips.de

Table of Contents

Supplementary tables 3
Supplementary figures 4
RNase activity with pre-incubation 4
NMR and HRMS spectra 5
Compound 1 5
Compound 2 7
Compound 3 9
Compound 5 11
Compound 6 13
Compound 9 16
Compound 10 19
Compound 11 22
Compound 12 25
Compound 13 28
Compound 14 31
Compound 15 33
Compound 16 36
Native MS spectra 39
SPR sensorgrams 47

Supplementary tables

Supplementary Table 1: Composition of DCC-1 experiment

Entry	Blank		Protein-templated (I)		Protein-templated (II)	
	amount	*Final conc. in DCL*	*amount*	*Final conc. in DCL*	*amount*	*Final conc. in DCL*
Phosphate buffer	950	-	906.5 µL	-	906.5 µL	-
Hydrazide (100 mM)	7 × 3 µL (21 µL)	(7 × 300 µM)	7 × 3 µL (21 µL)	(7 × 300 µM)	7 × 3 µL (21 µL)	(7 × 300 µM)
Aldehyde (100 mM)	3 × 1 µL (3 µL)	(3 × 100 µM)	3 × 1 µL (3 µL)	(3 × 100 µM)	3 × 1 µL (3 µL)	(3 × 100 µM)
Aniline (1 M)	10 µL	10 mM	10 µL	10 mM	10 µL	10 mM
DMSO	16 µL	-	16 µL	-	16 µL	-
Nsp10 (1.13 mM)	0	-	43.5 µL	50 µM	43.5 µL	50 µM

Supplementary Table 2: Composition of DCC-2 experiment

Entry	Blank		Protein-templated (I)		Protein-templated (II)	
	amount	*Final conc. in DCL*	*amount*	*Final conc. in DCL*	*amount*	*Final conc. in DCL*
Phosphate buffer	947	-	933.3 µL	-	933.3 µL	-
Hydrazide (100 mM)	8 × 3 µL (24 µL)	(8 × 300 µM)	8 × 3 µL (24 µL)	(8 × 300 µM)	8 × 3 µL (24 µL)	(8 × 300 µM)
Aldehyde (100 mM)	3 × 1 µL (3 µL)	(3 × 100 µM)	3 × 1 µL (3 µL)	(3 × 100 µM)	3 × 1 µL (3 µL)	(3 × 100 µM)
Aniline (1 M)	10 µL	10 mM	10 µL	10 mM	10 µL	10 mM
DMSO	16 µL	-	16 µL	-	16 µL	-
Nsp10 (3.66 mM)	0	-	13.7 µL	50 µM	13.7 µL	50 µM

Supplementary Table 3: Determination of T_h of Nsp10 using Thermal shift assay (TSA)[a]

**Nsp10**	**T_h °C**		
**DCC conditions**	**t = 0h**	**t = 24h**	**t = 48h**
Phosphate buffer, pH = 7.04, rt	49.9 ± 0.0	49.7 ± 0.2	50 ± 0.5
Phosphate buffer, pH = 7.04, rt, DMSO (5%)	49.4 ± 0.5	49.9 ± 0.0	50 ± 0.

[a]*The experiment was performed in technical duplicates.*

Supplementary figures

RNase activity with pre-incubation

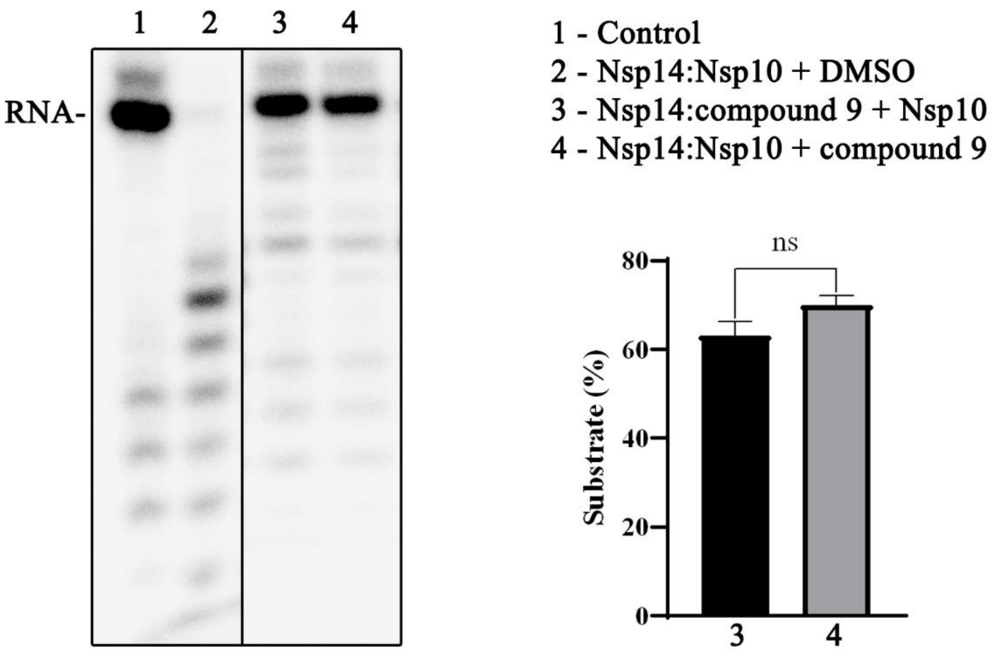

Supplementary Figure 1 - Effect of compound **9** in the 3‘-5‘ exoribonuclease activity of SARS-CoV-2 Nsp14. (1) Control reaction with no enzymes (2) Nsp14 and Nsp10 were incubated with the RNA substrate in the presence of DMSO (3) Nsp10 and compound **9** were pre-incubated prior to the addition of Nsp14 and the RNA substrate (4) Nsp14 and Nsp10 were pre-incubated to form the complex before the addition of compound **9** and the RNA substrate. The concentrations used in the assays were: 500 nM of Nsp14, 2000 nM of Nsp10, 50 nM of RNA substrate and 2000 µM of compound **9**. On the left, a representative activity assay, where the reactions were analysed on a 7 M urea / 20 % polyacrylamide gel; on the right, the quantification of the amount of substrate present at the end of the reactions 3 and 4 was determined.

NMR and HRMS spectra

Compound 1

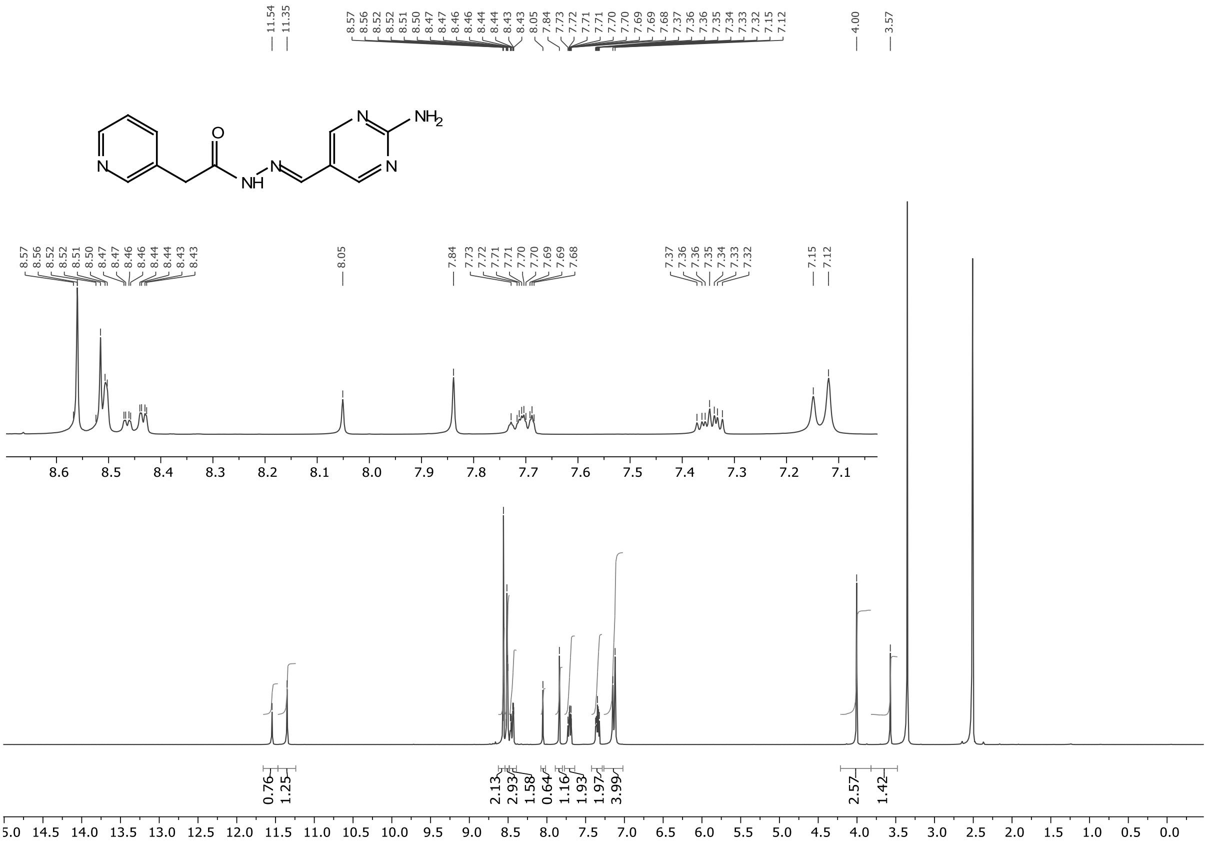

Supplementary Figure 2 ^{1}H spectrum of **1**

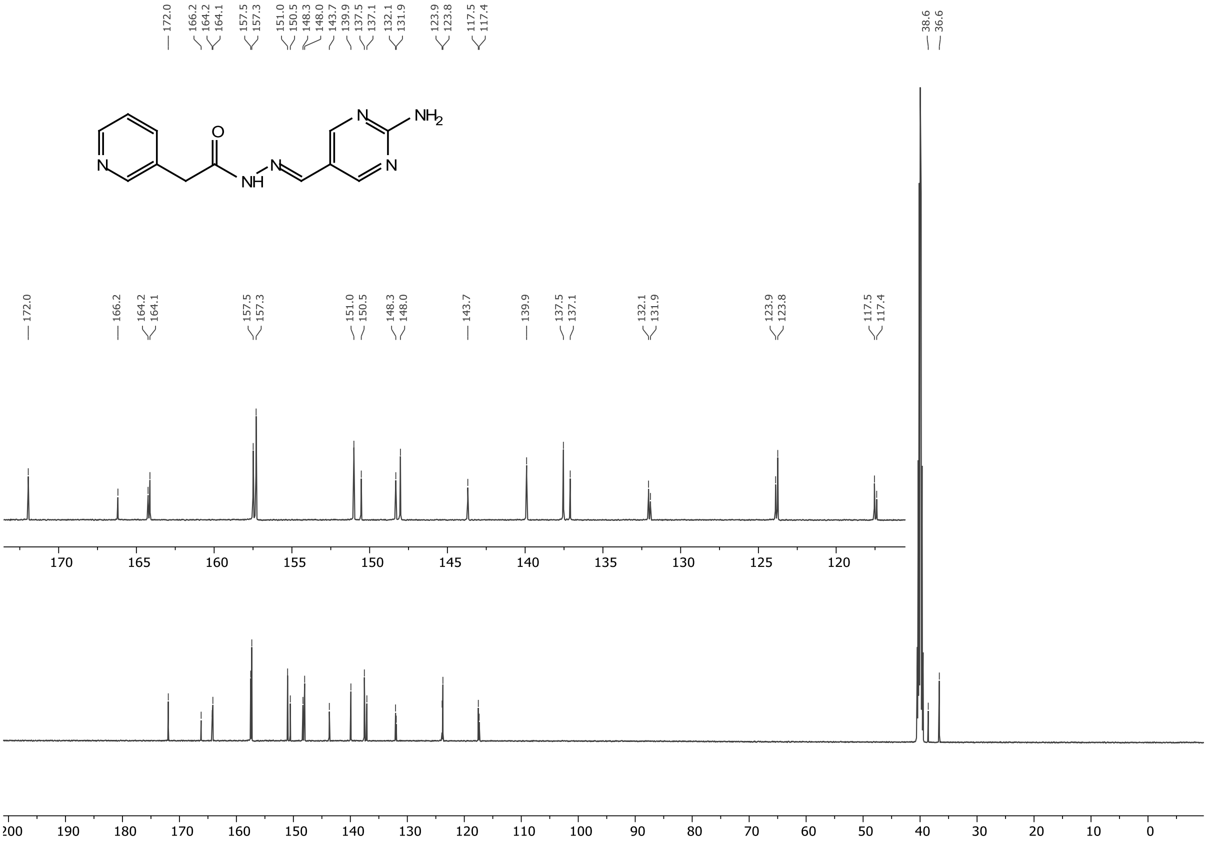

Supplementary Figure 3 ^{13}C spectrum of **1**

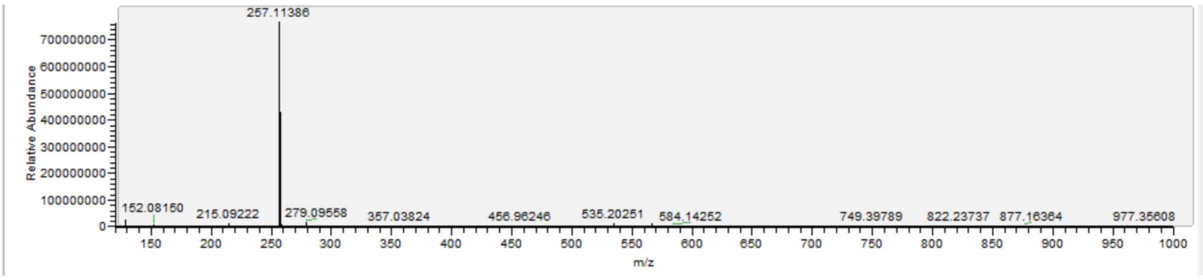

Supplementary Figure 4 HRMS of **1**

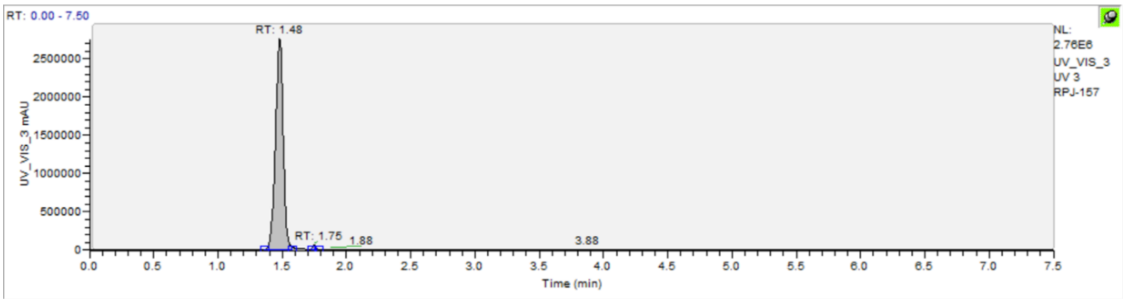

No	Ret. Time min	Rel. Area %
1	1.48	98.79
2	1.75	1.21

Supplementary Figure 5 LCMS purity of **1**

Compound 2

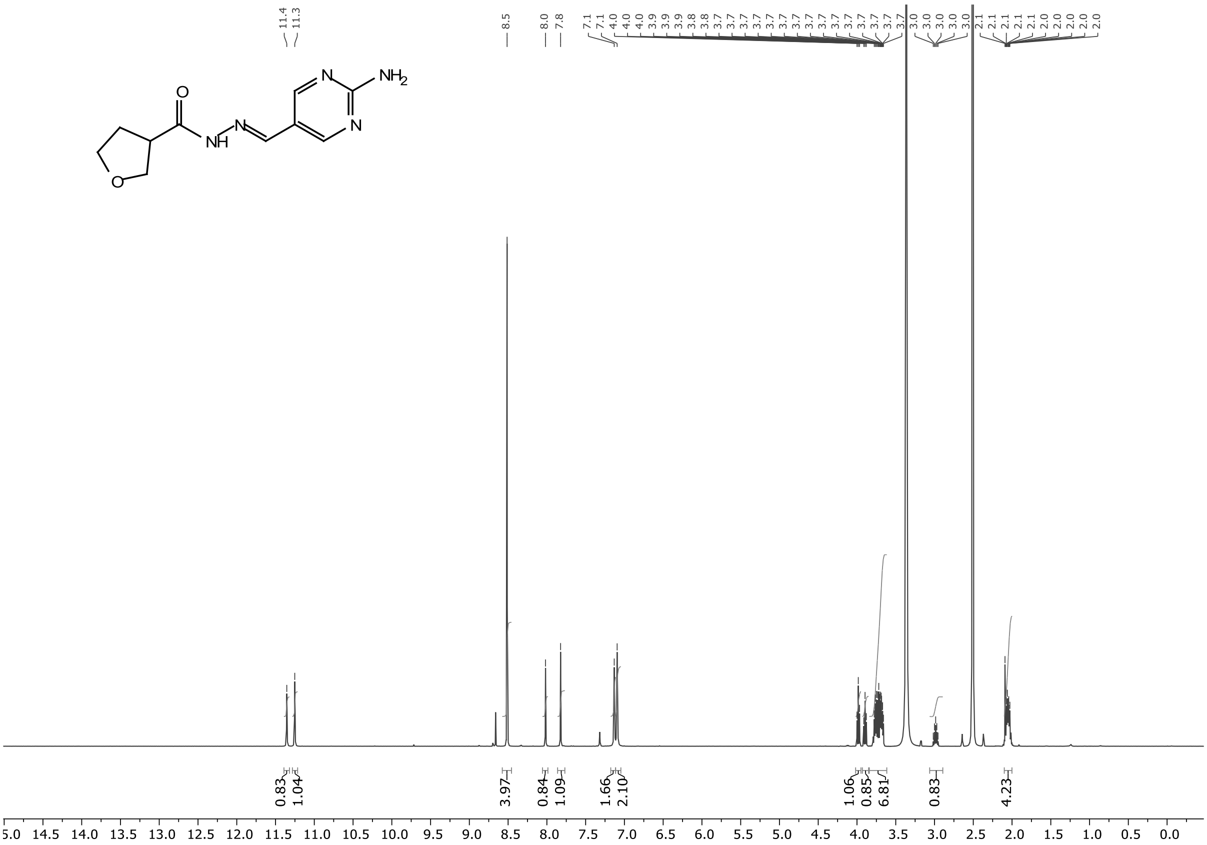

Supplementary Figure 6 ^{1}H spectrum of **2**

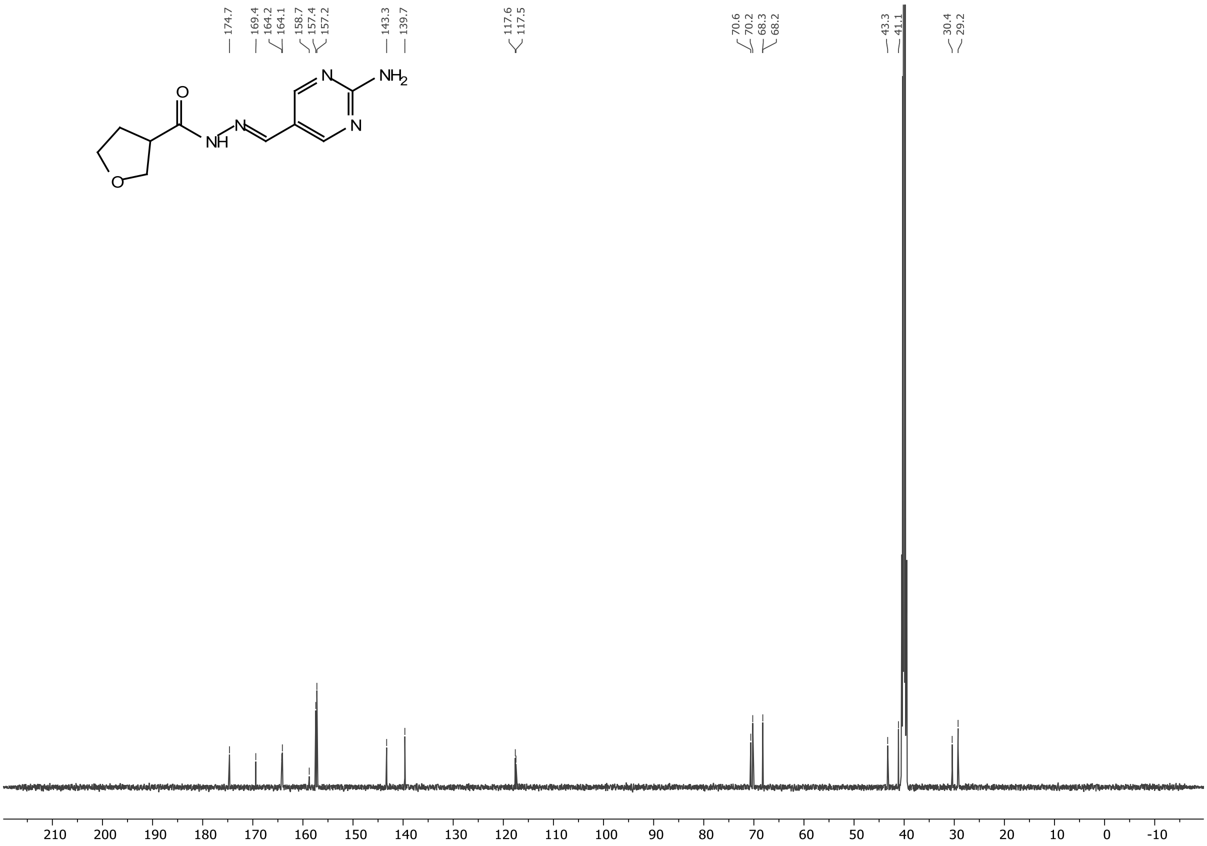

Supplementary Figure 7 ^{13}C spectrum of **2**

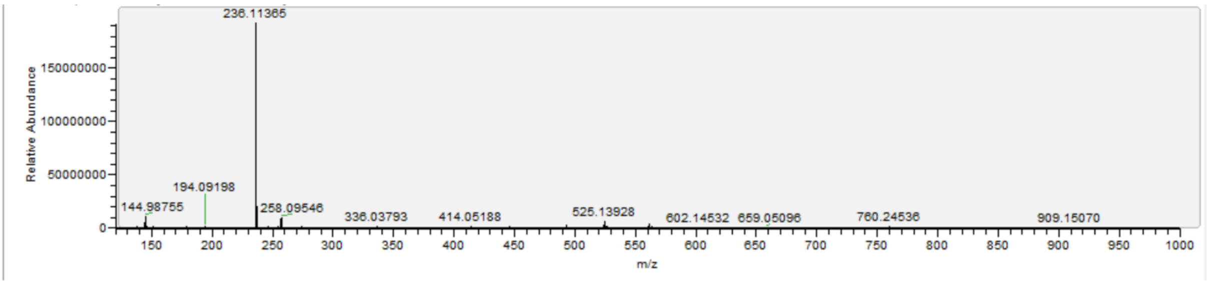

Supplementary Figure 8 HRMS of **2**

Compound 3

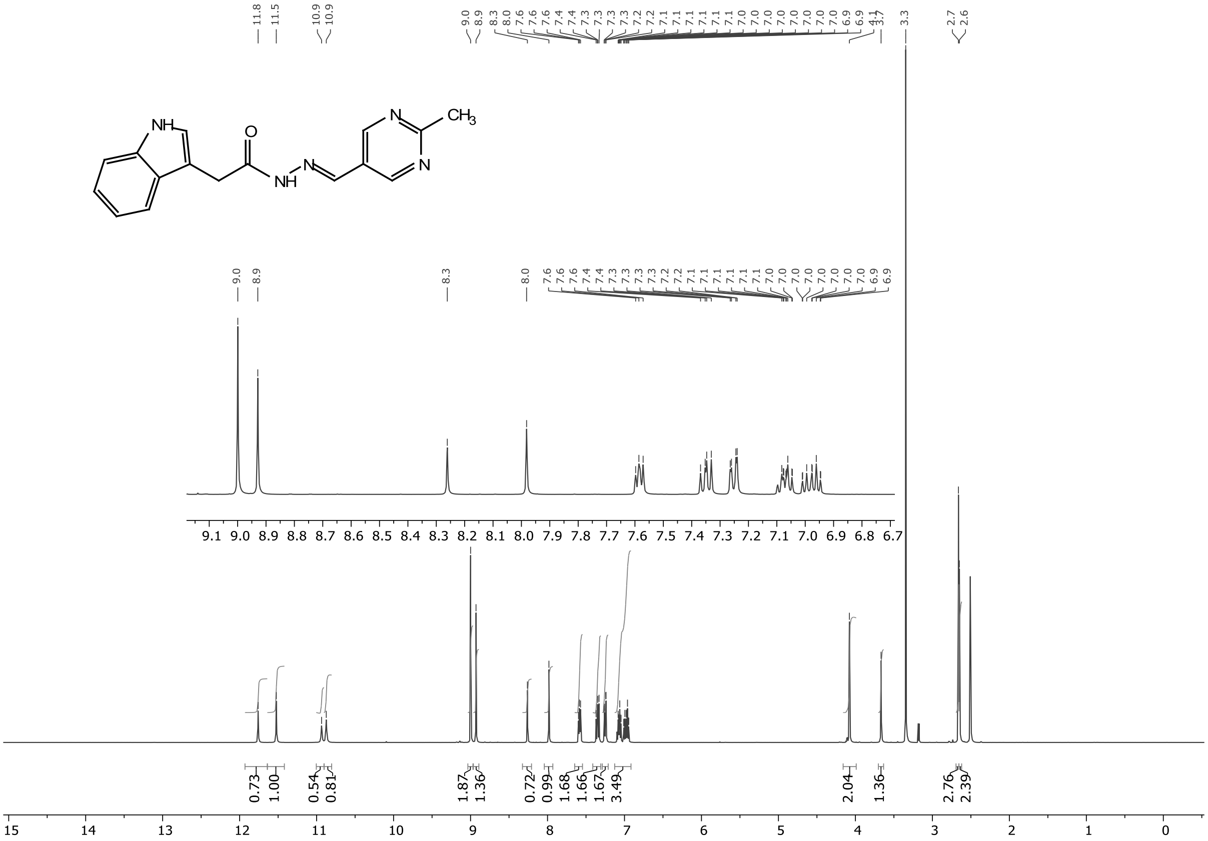

Supplementary Figure 9 ^{1}H spectrum of **3**

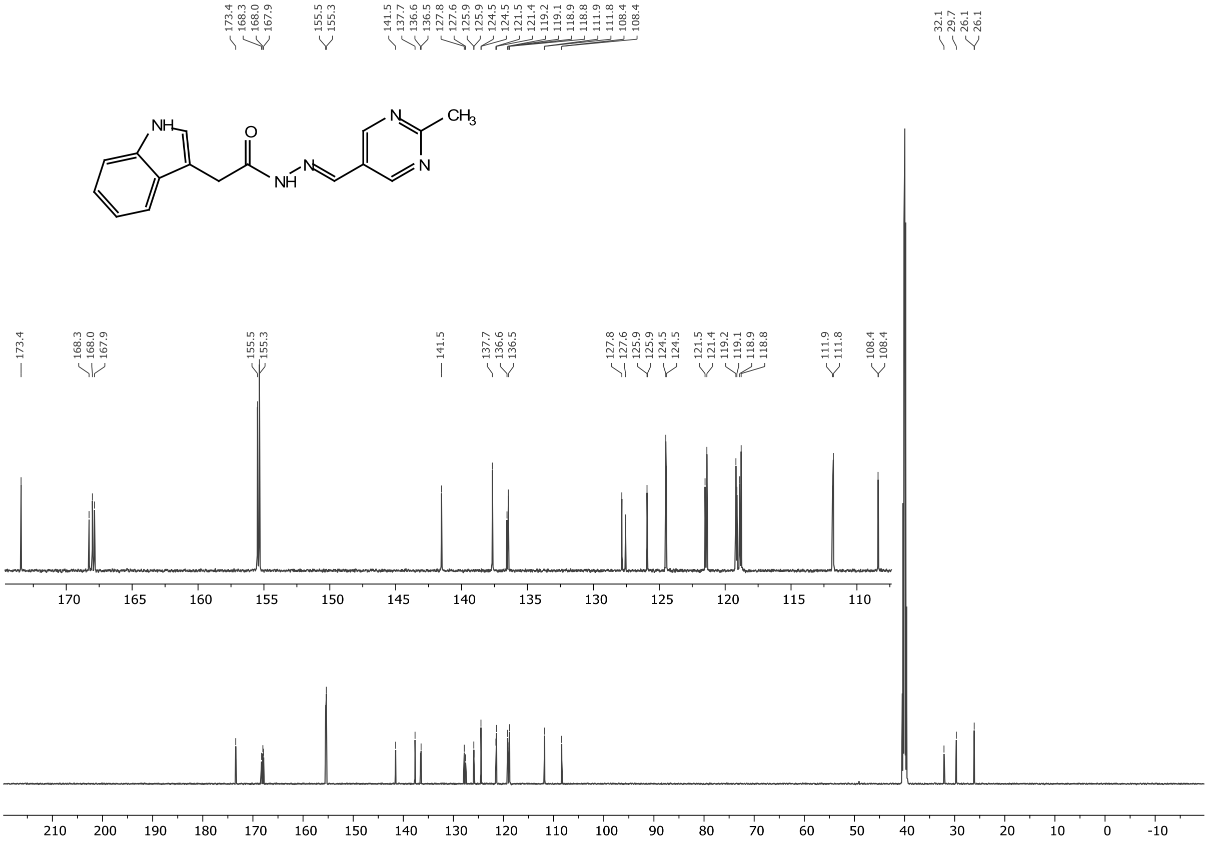

Supplementary Figure 10 ^{13}C spectrum of **3**

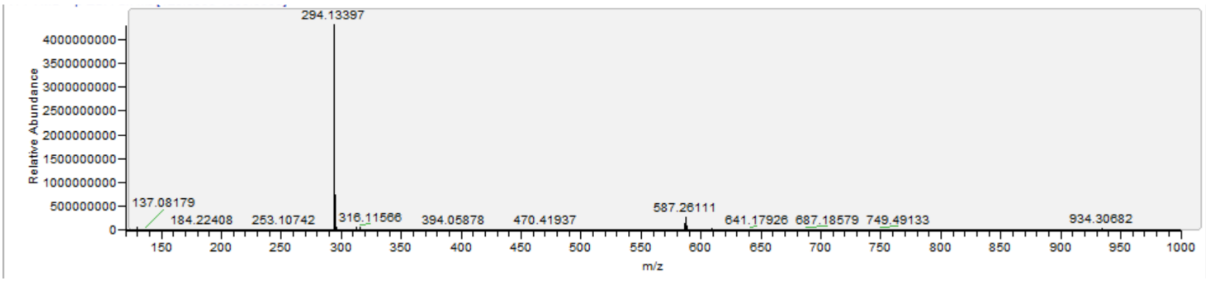

Supplementary Figure 11 HRMS of **3**

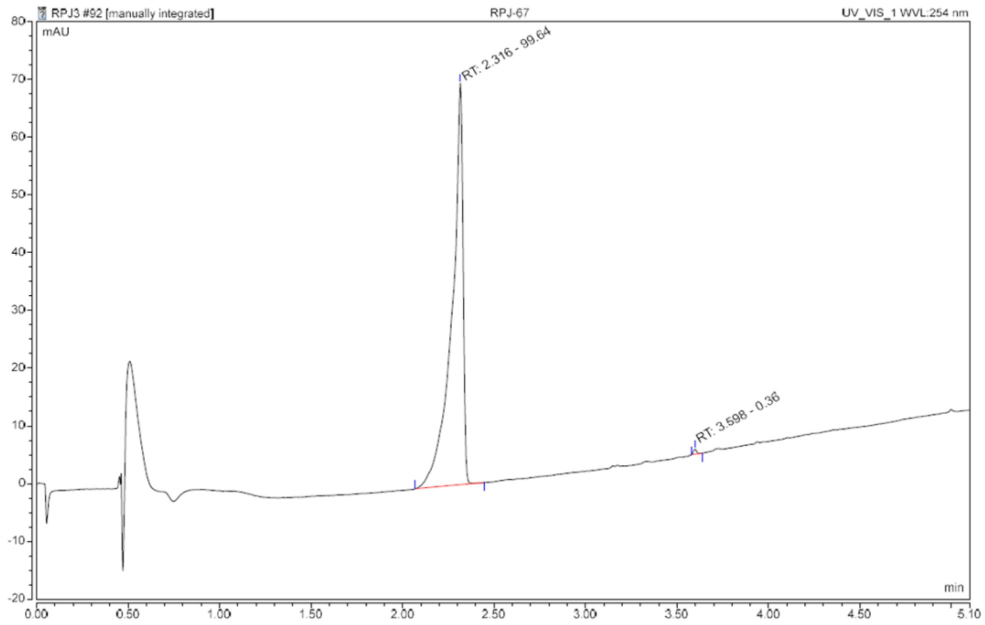

No.	Ret.Time min	Rel.Area %
1	2.316	99.64
2	3.598	0.36

Supplementary Figure 12 LCMS of **3**

Compound 5

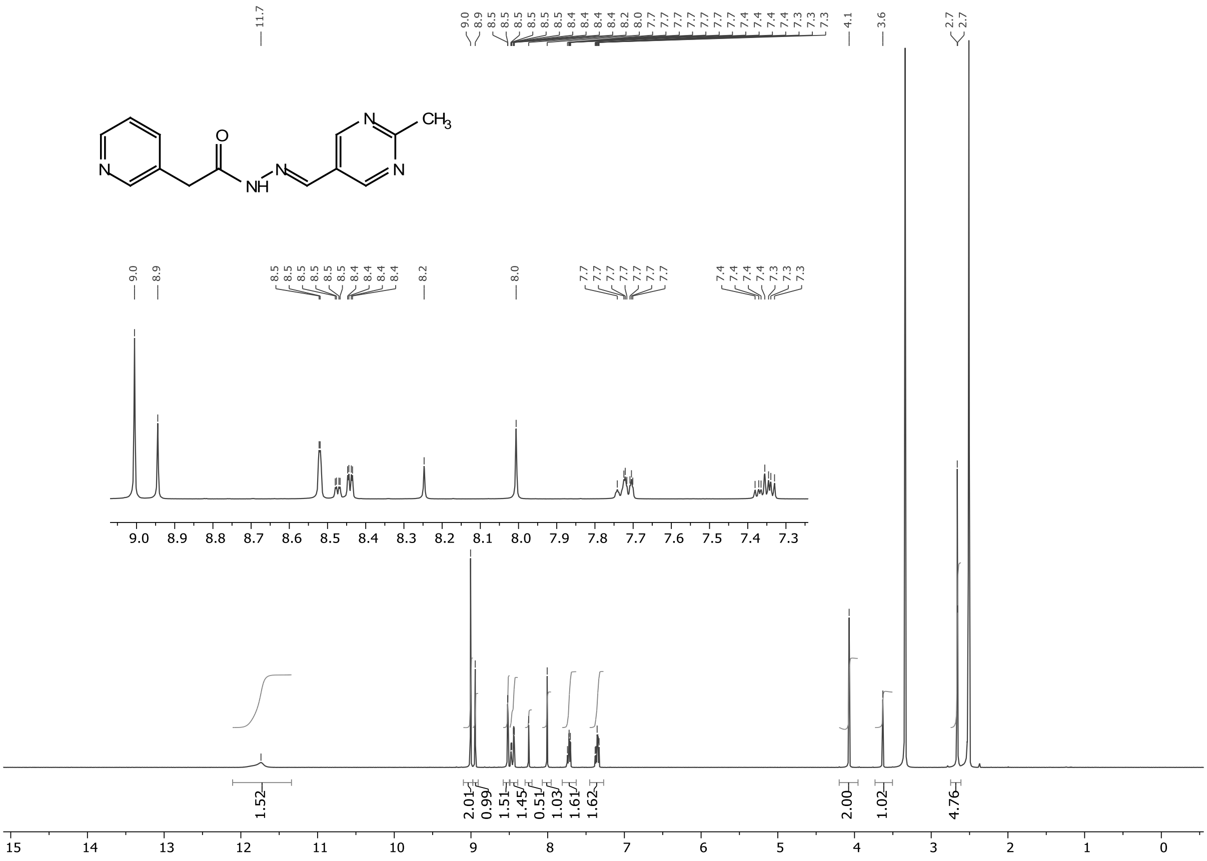

Supplementary Figure 13 ^{1}H spectrum of **5**

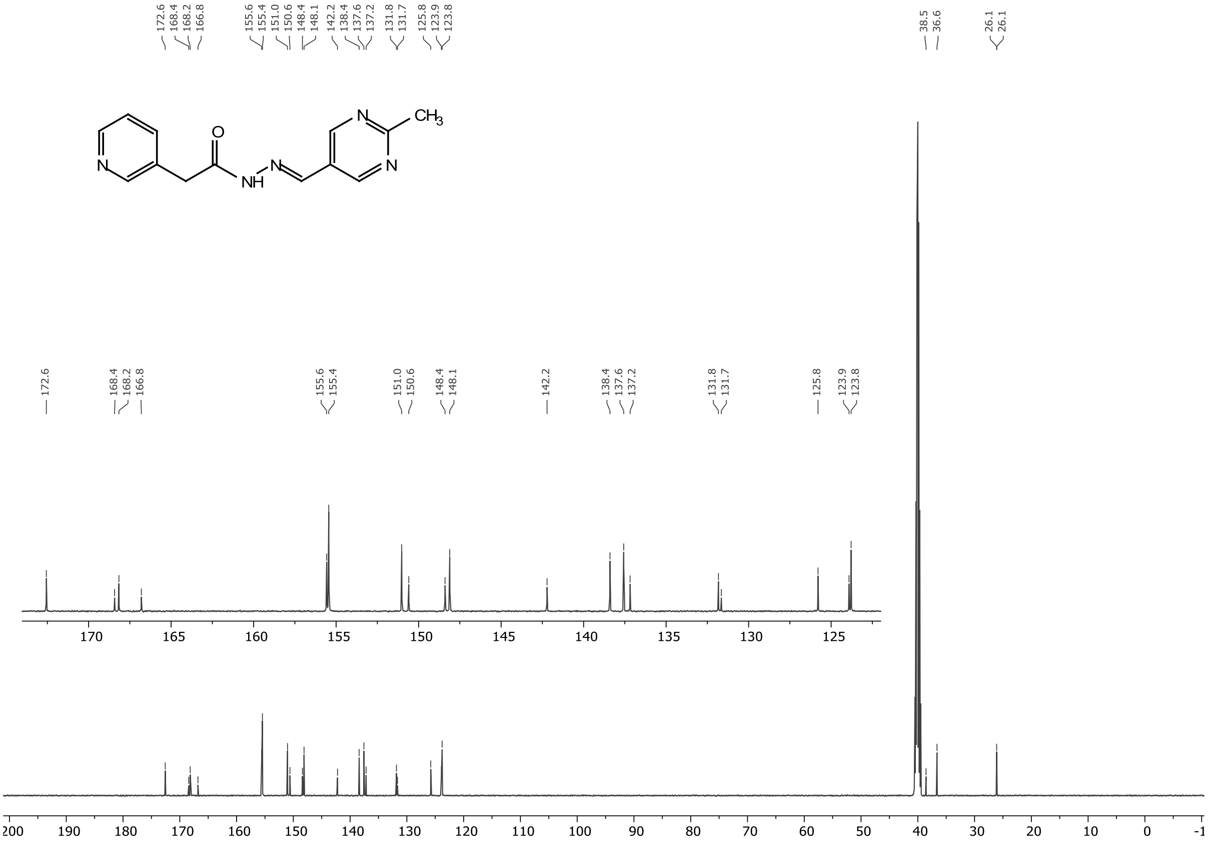

Supplementary Figure 14 ^{13}C spectrum of **5**

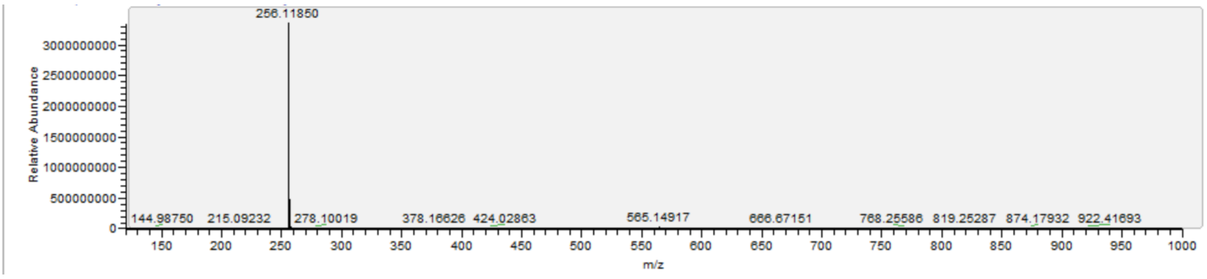

Supplementary Figure 15 HRMS of **5**

Compound 6

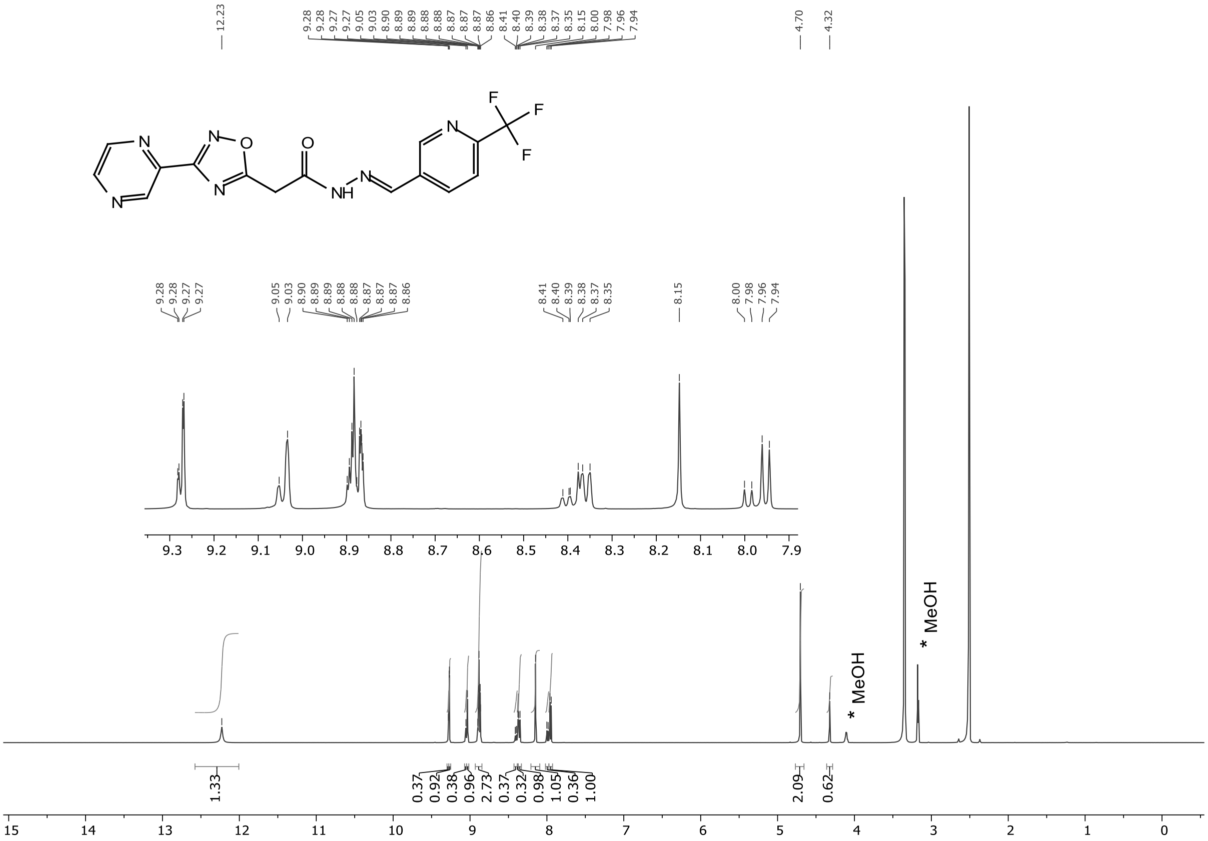

Supplementary Figure 16 ^{1}H spectrum of **6**

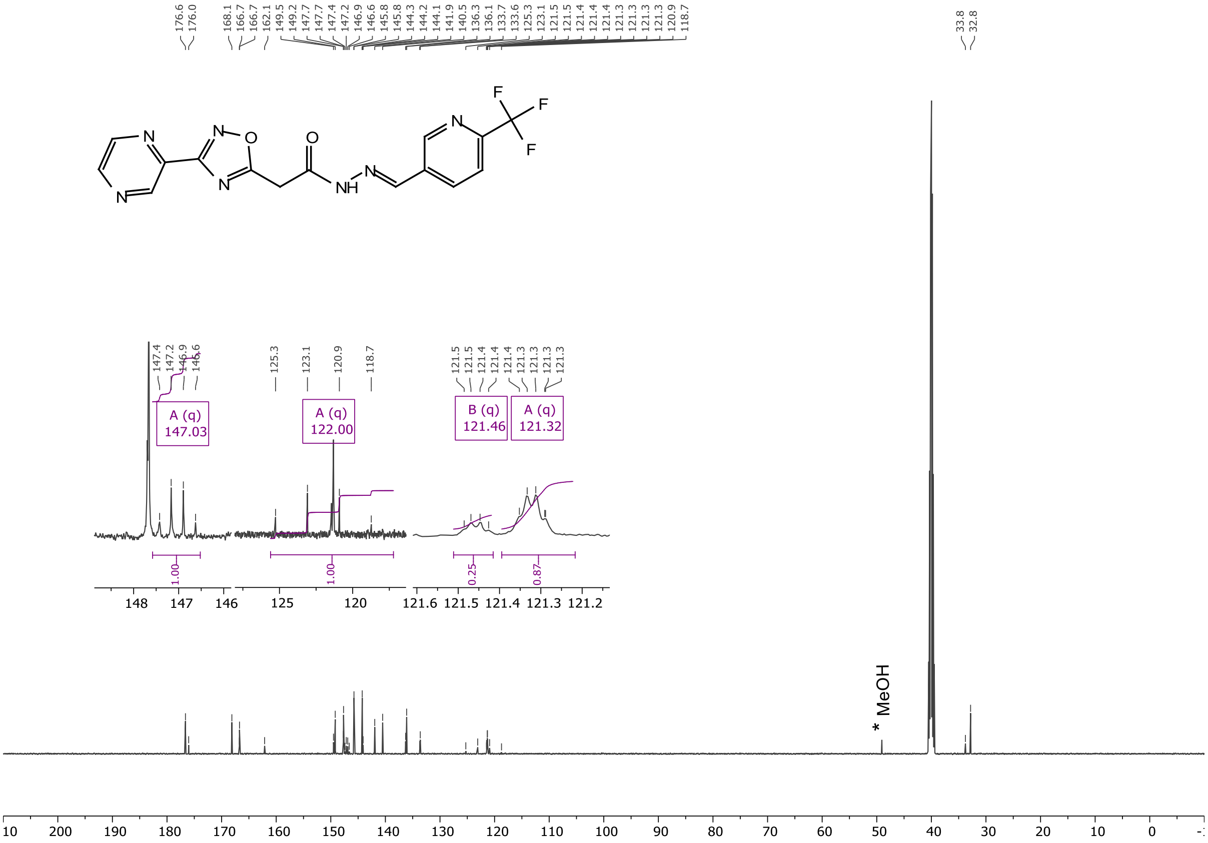

Supplementary Figure 17 ^{13}C spectrum of **6**

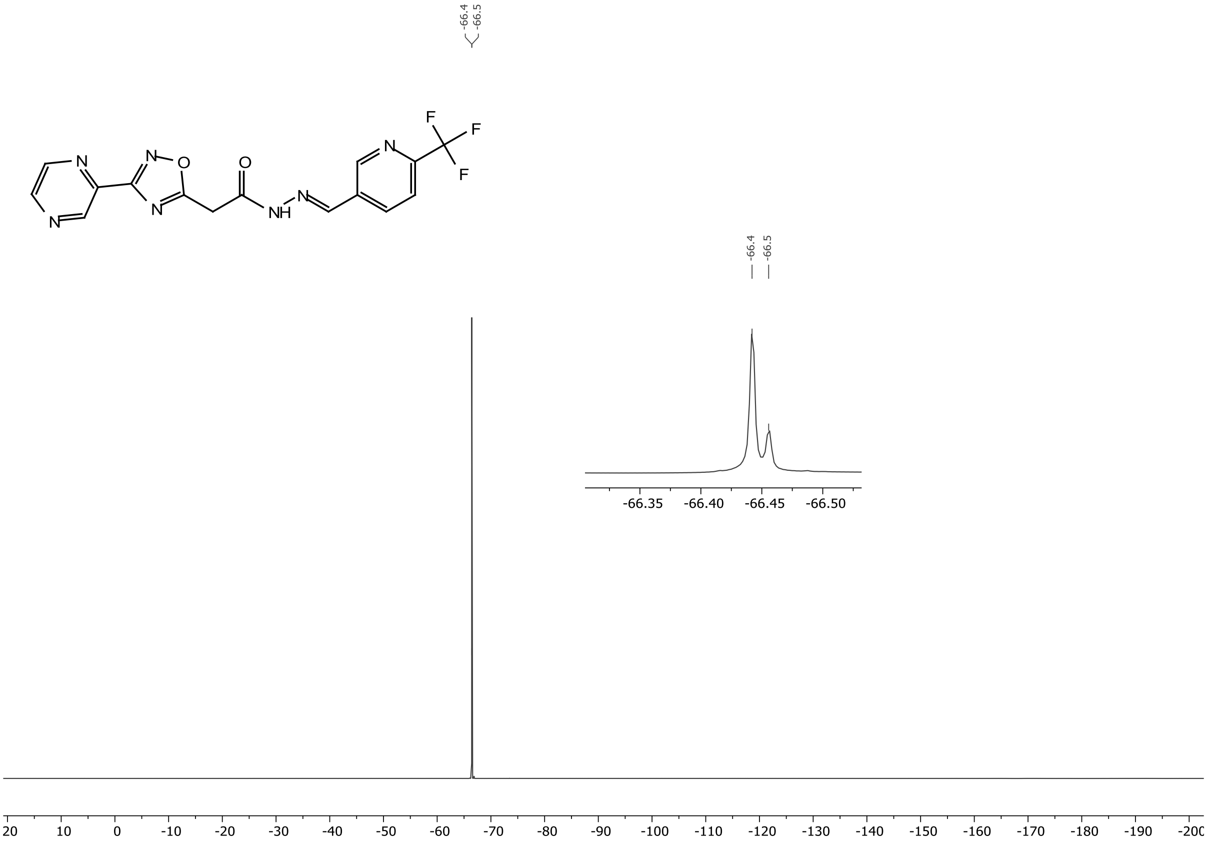

Supplementary Figure 18 ^{19}F spectrum of **6**

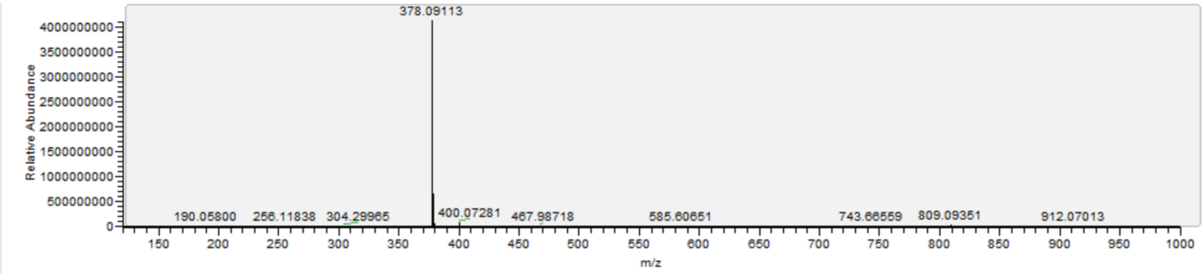

Supplementary Figure 19 HRMS of **6**

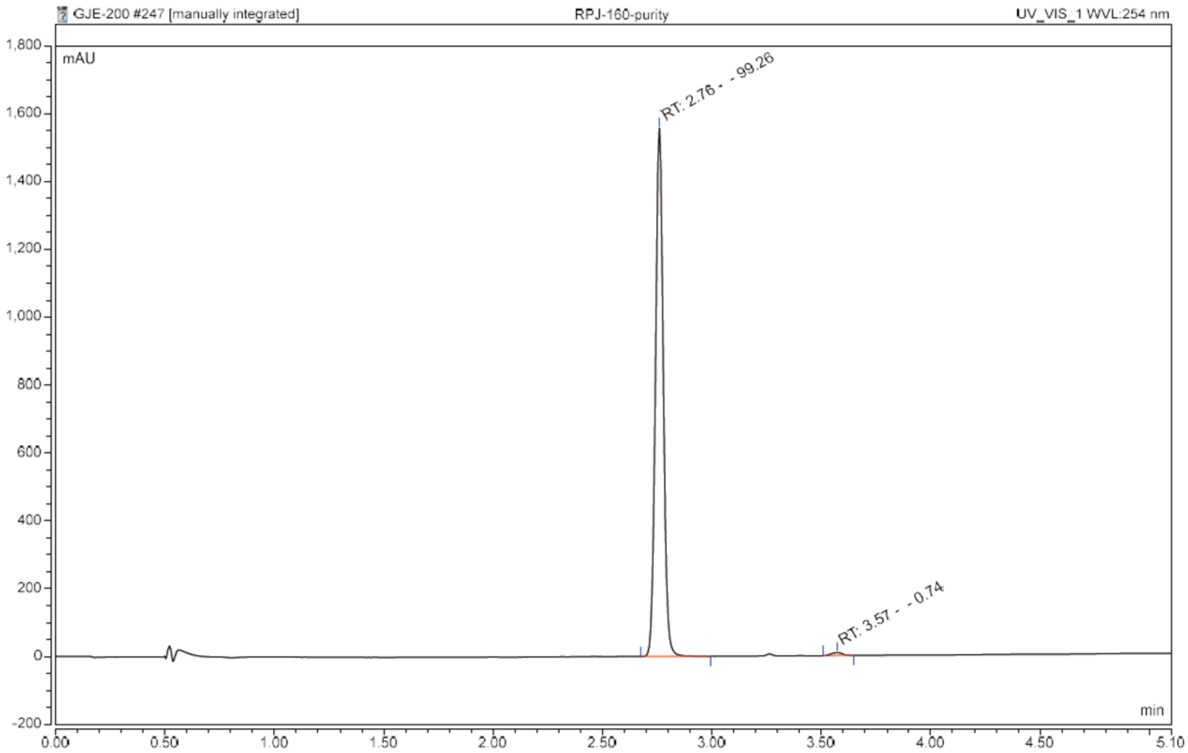

No.	Ret.Time min	Rel.Area %
1	2.760	99.26
2	3.573	0.74

Supplementary Figure 20 LCMS purity of **6**

Compound 9

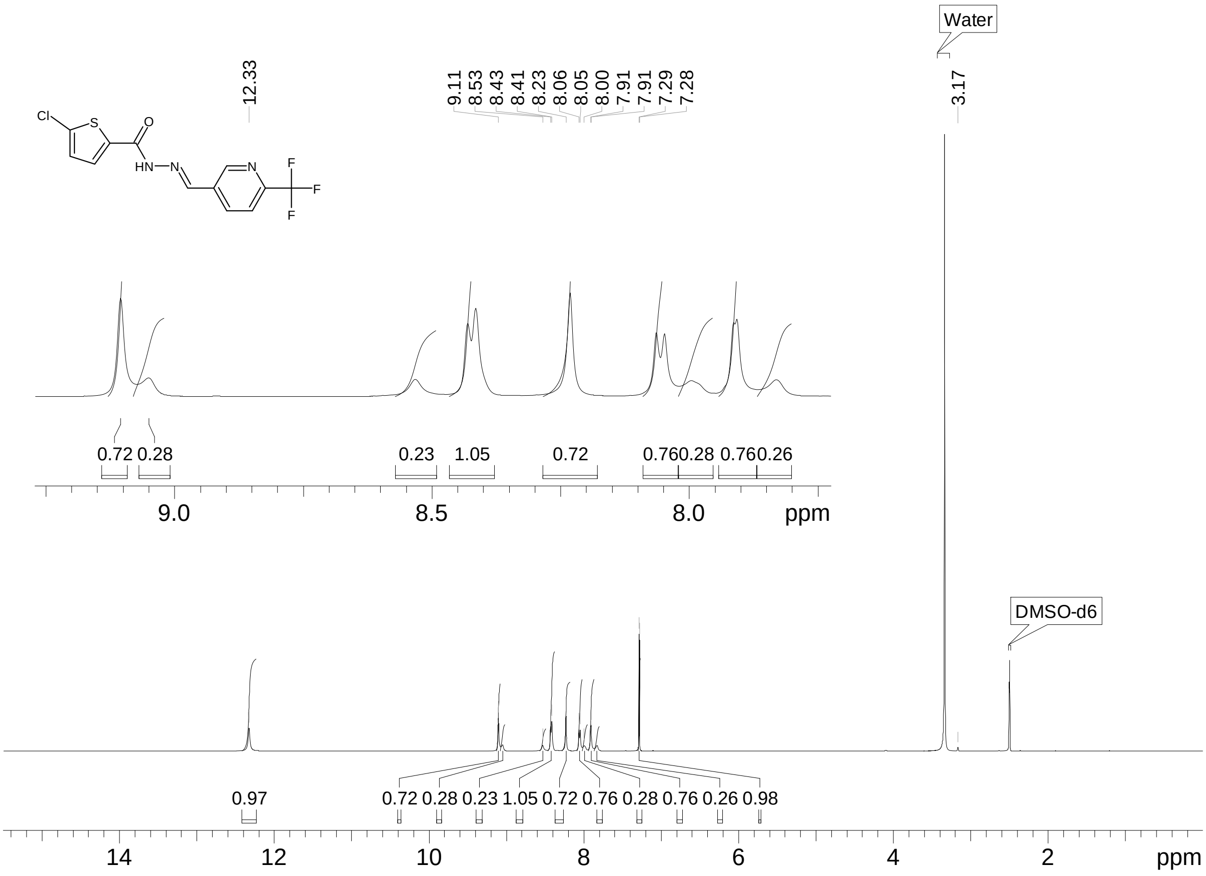

Supplementary Figure 21 ^{1}H spectrum of **9**

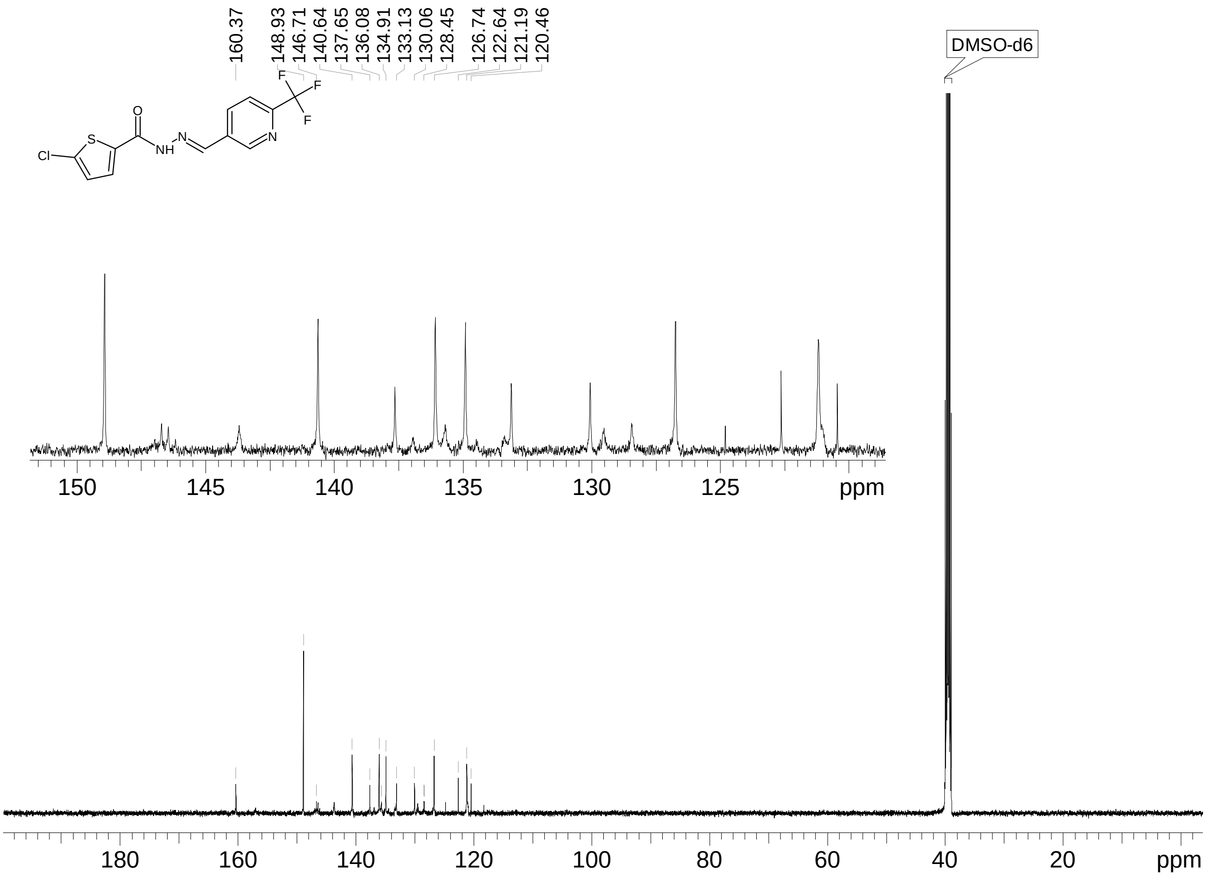

Supplementary Figure 22 ^{13}C spectrum of **9**

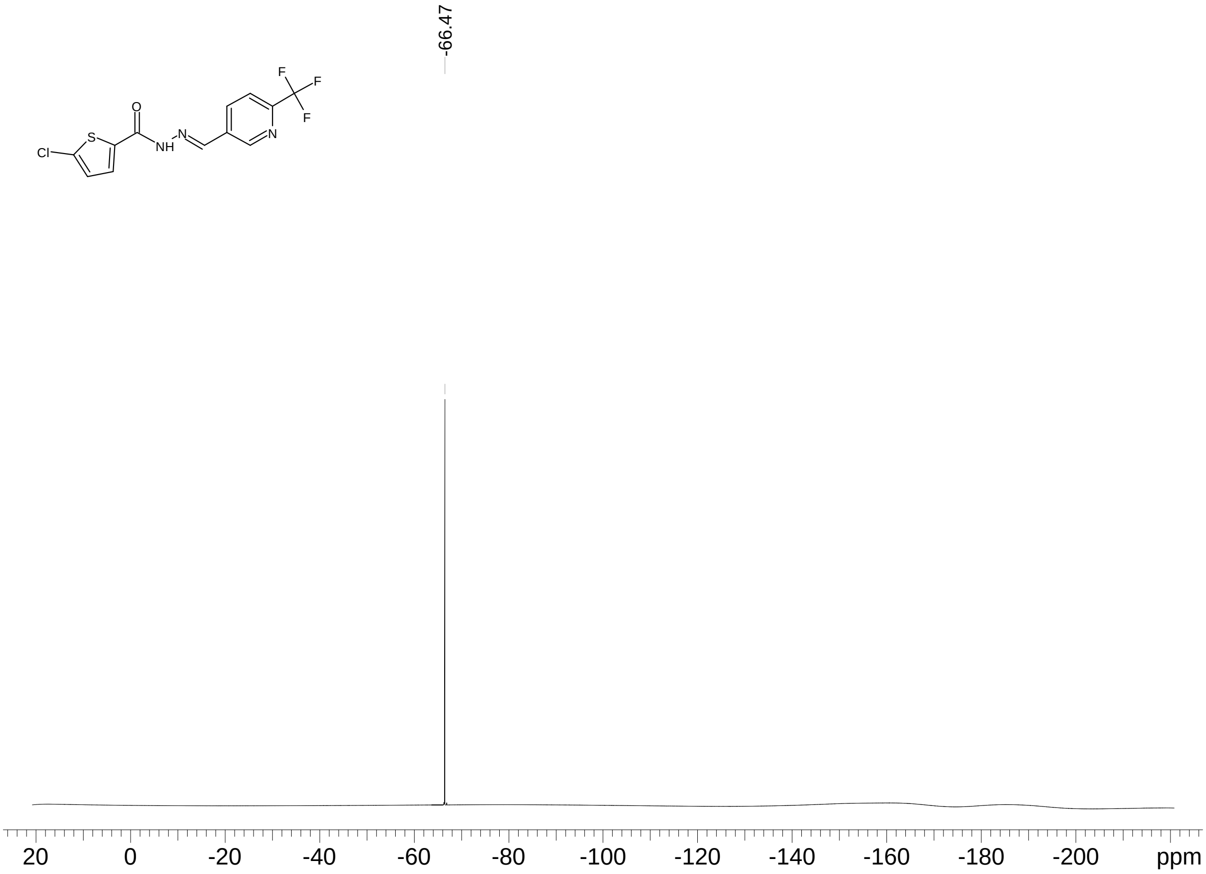

Supplementary Figure 23 ^{19}F spectrum of **9**

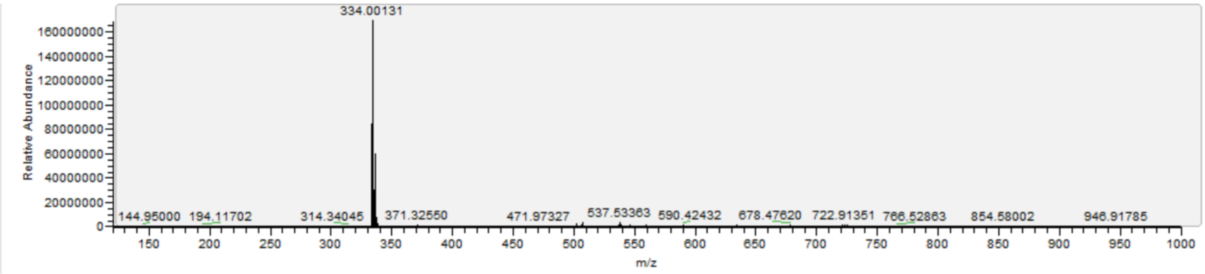

Supplementary Figure 24 HRMS of **9**

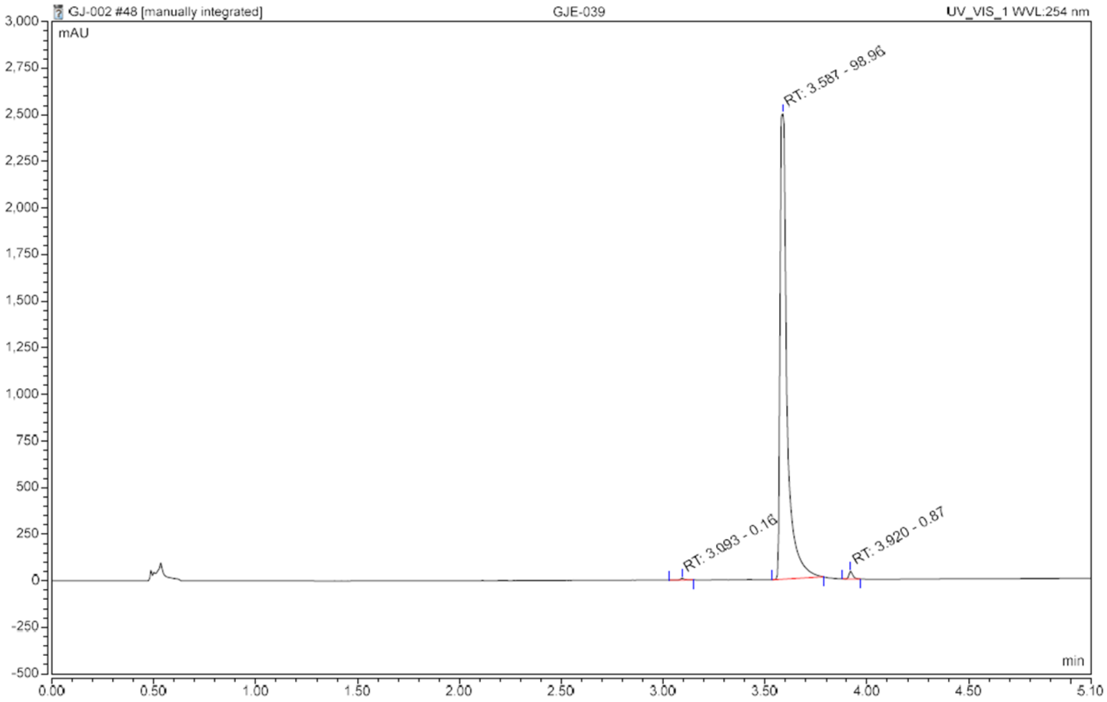

No.	Ret.Time min	Rel.Area %
1	3.093	0.16
2	3.587	98.96
3	3.920	0.87

Supplementary Figure 25 LCMS purity of **9**

Compound 10

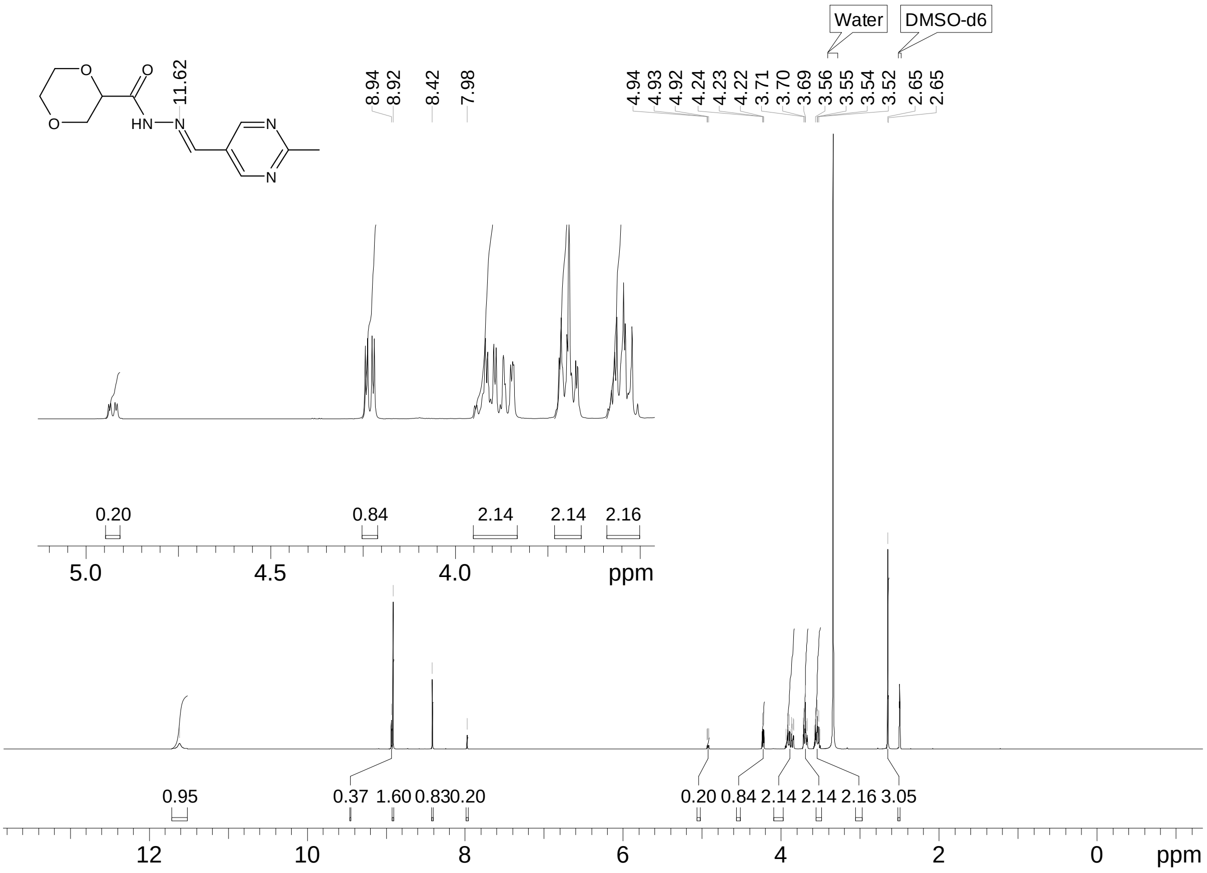

Supplementary Figure 26 ^{1}H spectrum of **10**

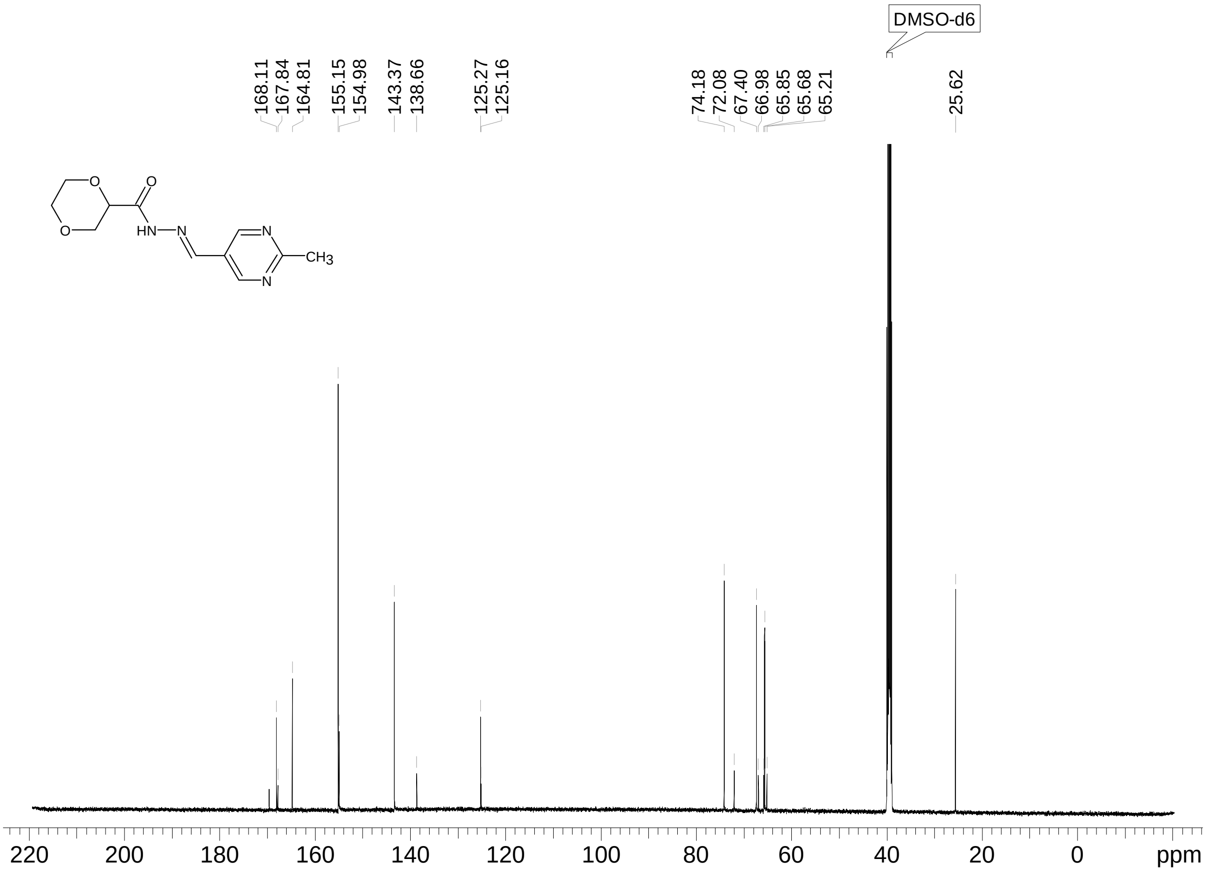

Supplementary Figure 27 ^{13}C spectrum of **10**

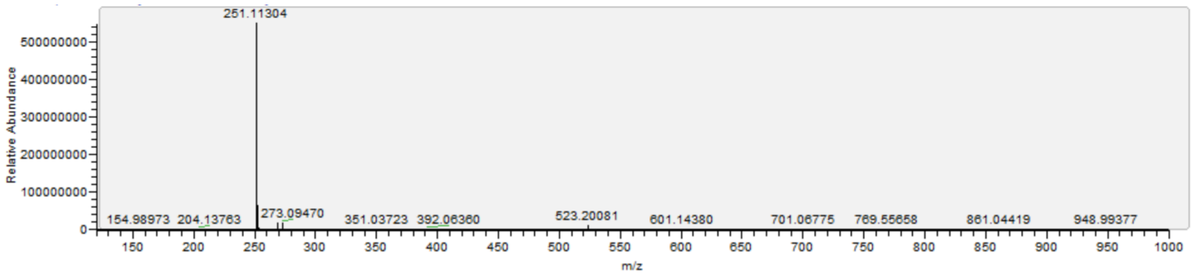

Supplementary Figure 28 HRMS of **10**

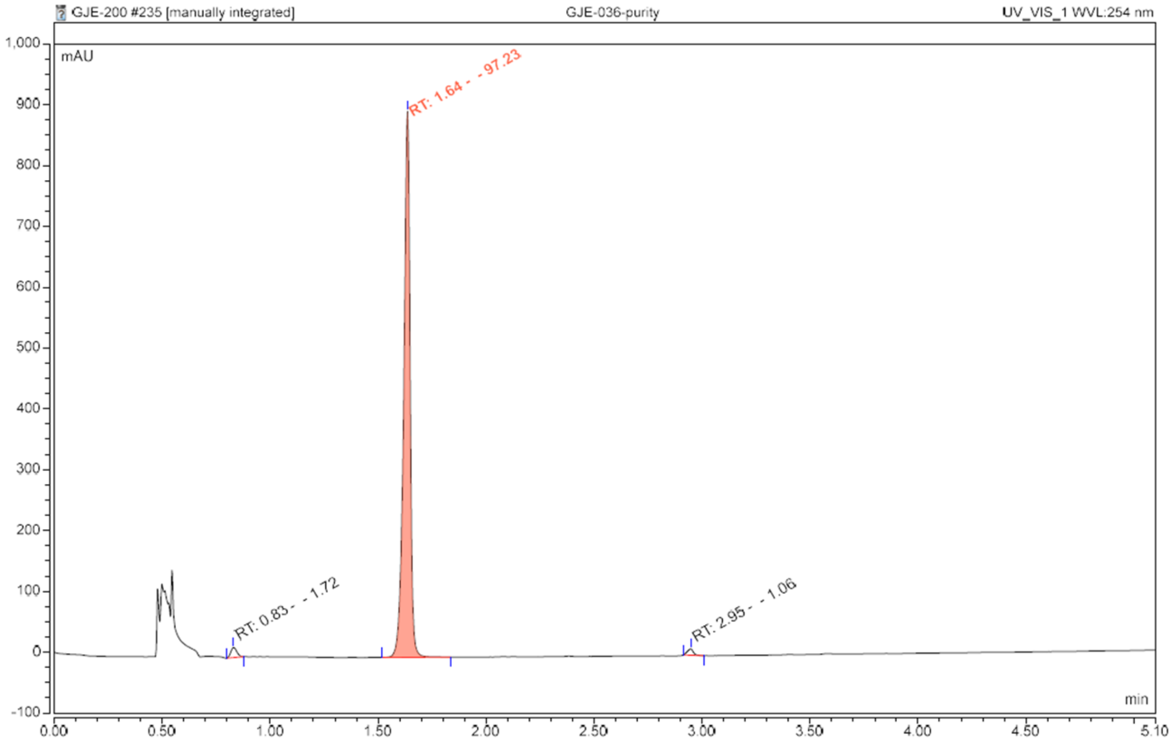

No.	Ret.Time min	Rel.Area %
1	0.830	1.72
2	1.636	97.23
3	2.947	1.06

Supplementary Figure 29 LCMS purity of **10**

Compound 11

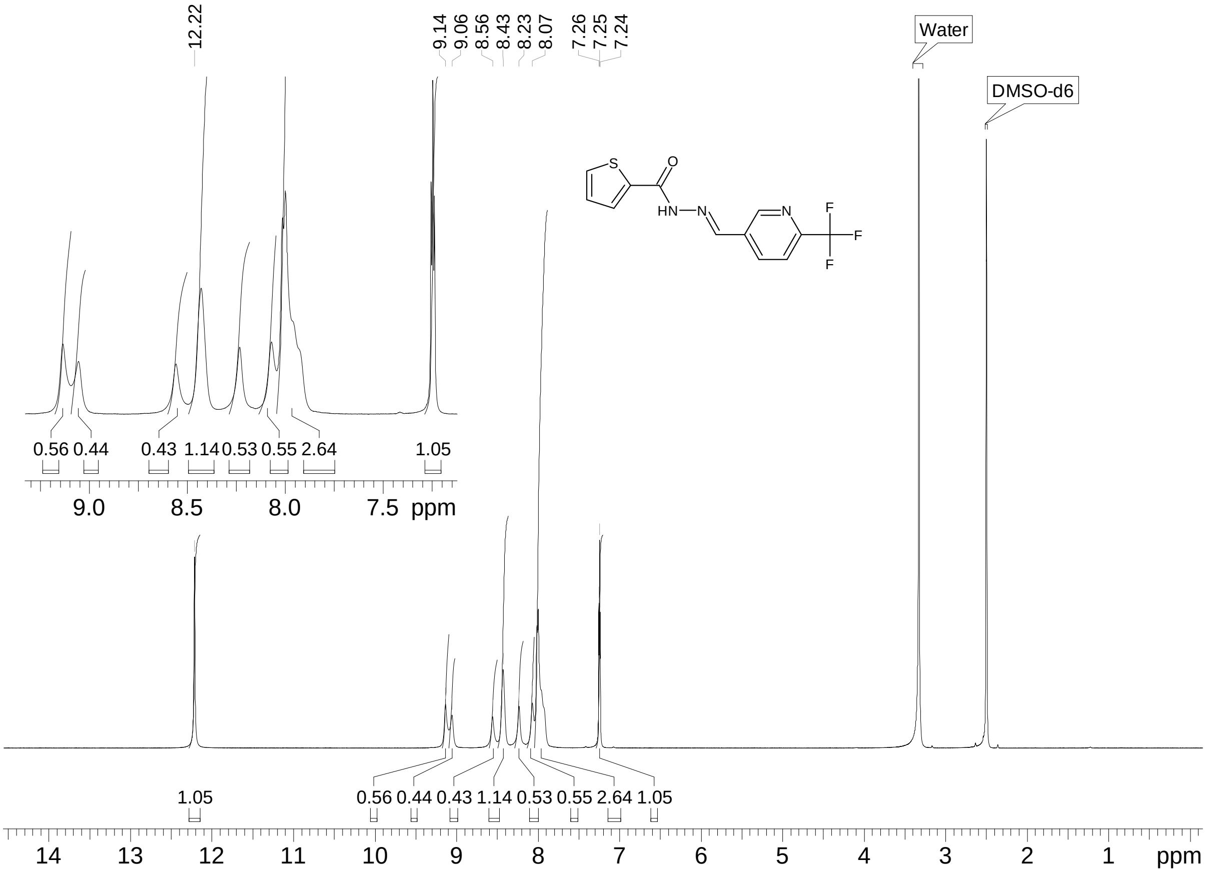

Supplementary Figure 30 ^{1}H spectrum of **11**

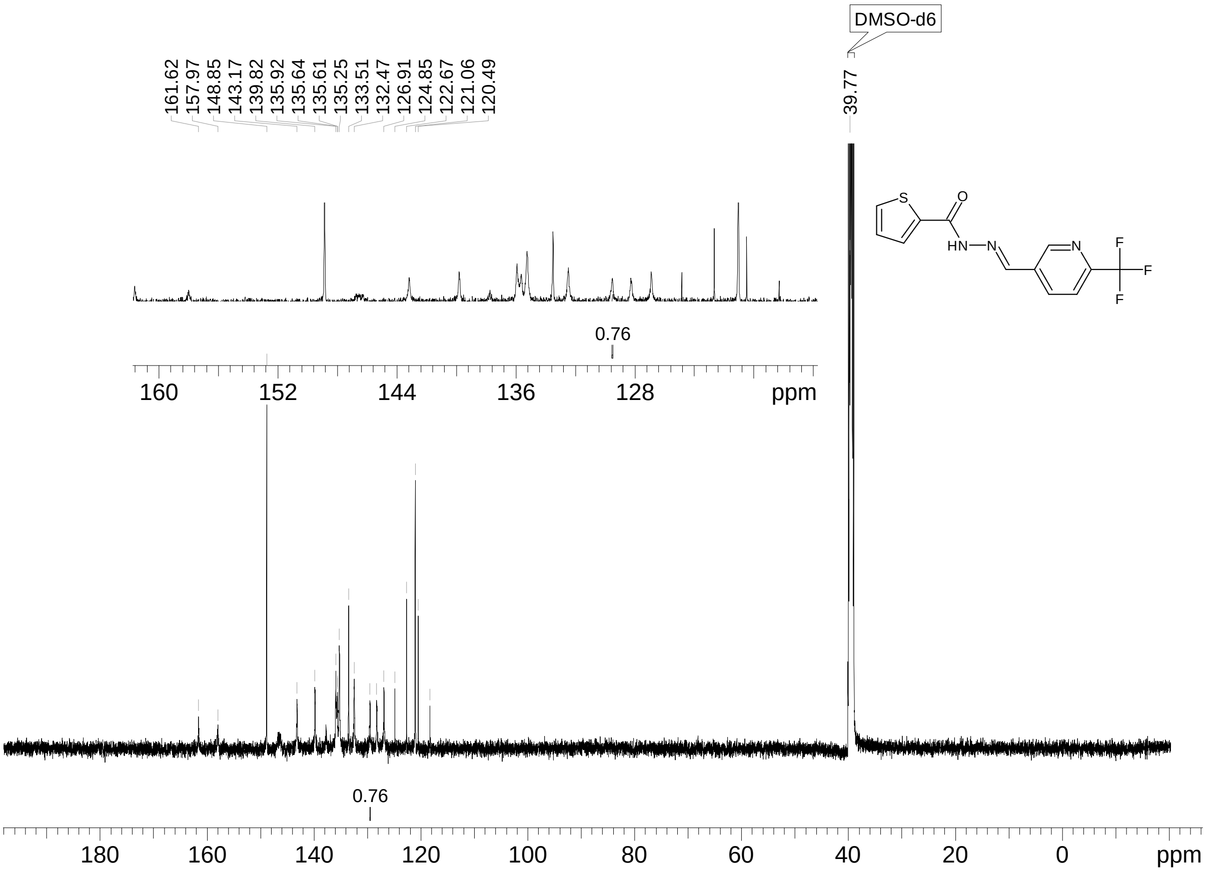

Supplementary Figure 31 ^{13}C spectrum of **11**

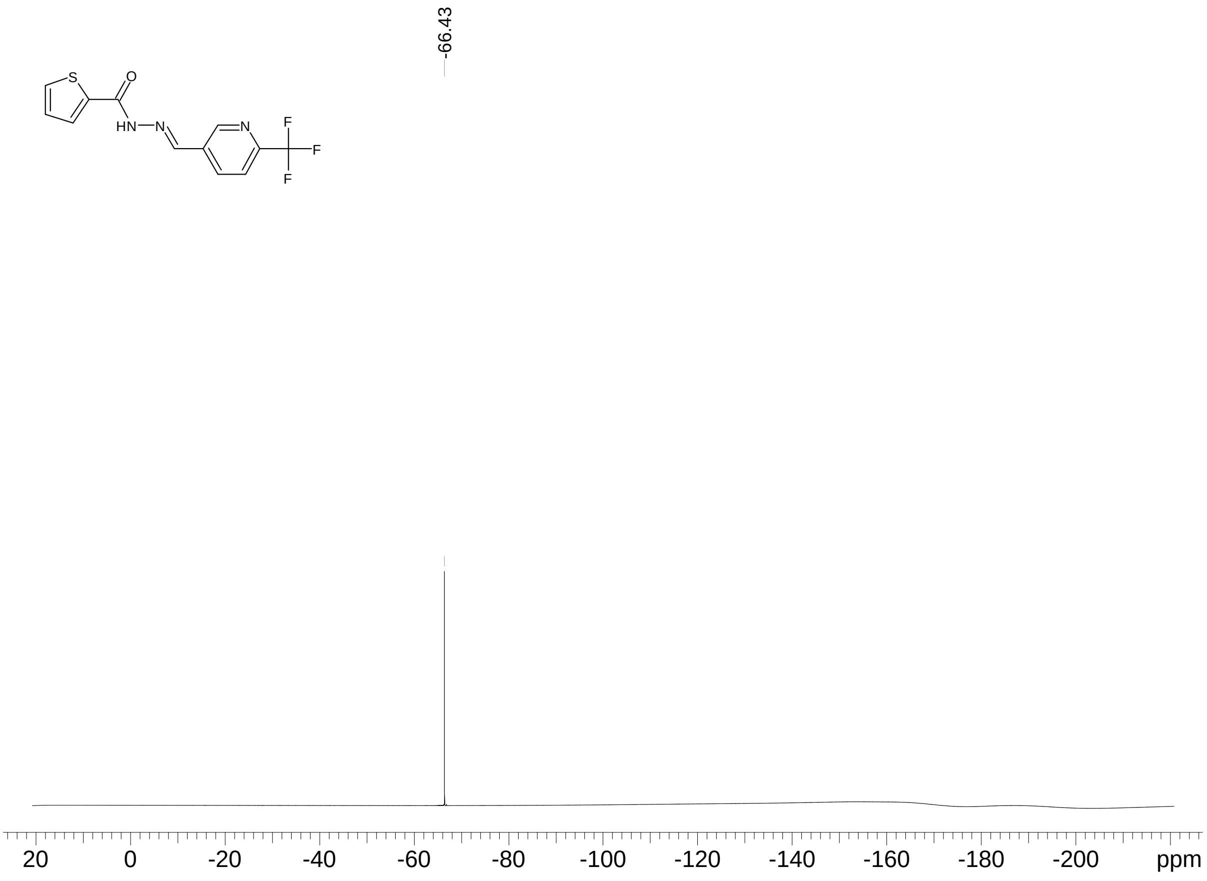

Supplementary Figure 32 ^{19}F spectrum of **11**

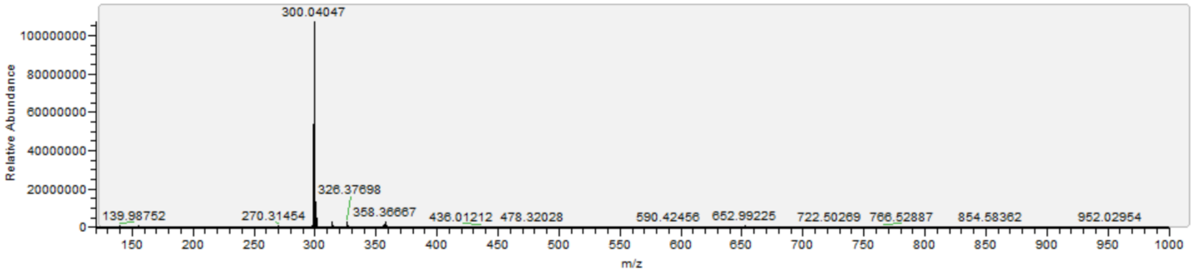

Supplementary Figure 33 HRMS of **11**

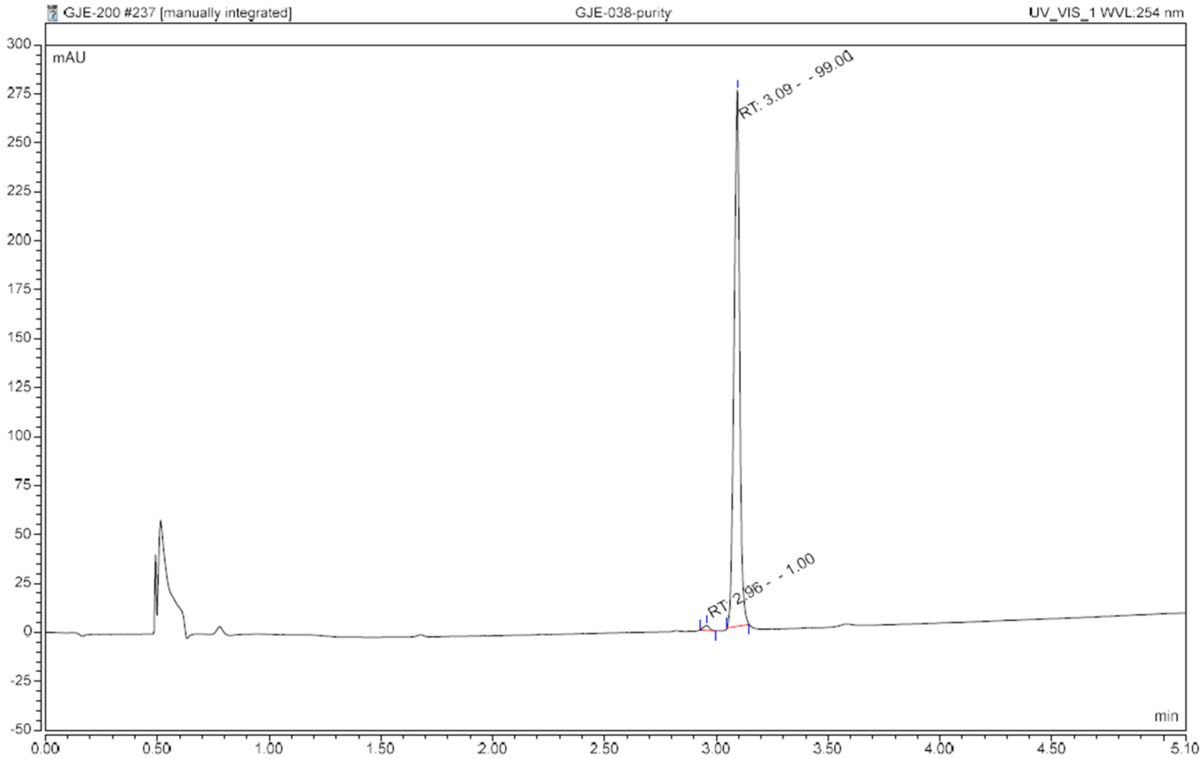

No.	Ret.Time min	Rel.Area %
1	2.957	1.00
2	3.094	99.00

Supplementary Figure 34 LCMS purity of **11**

Compound 12

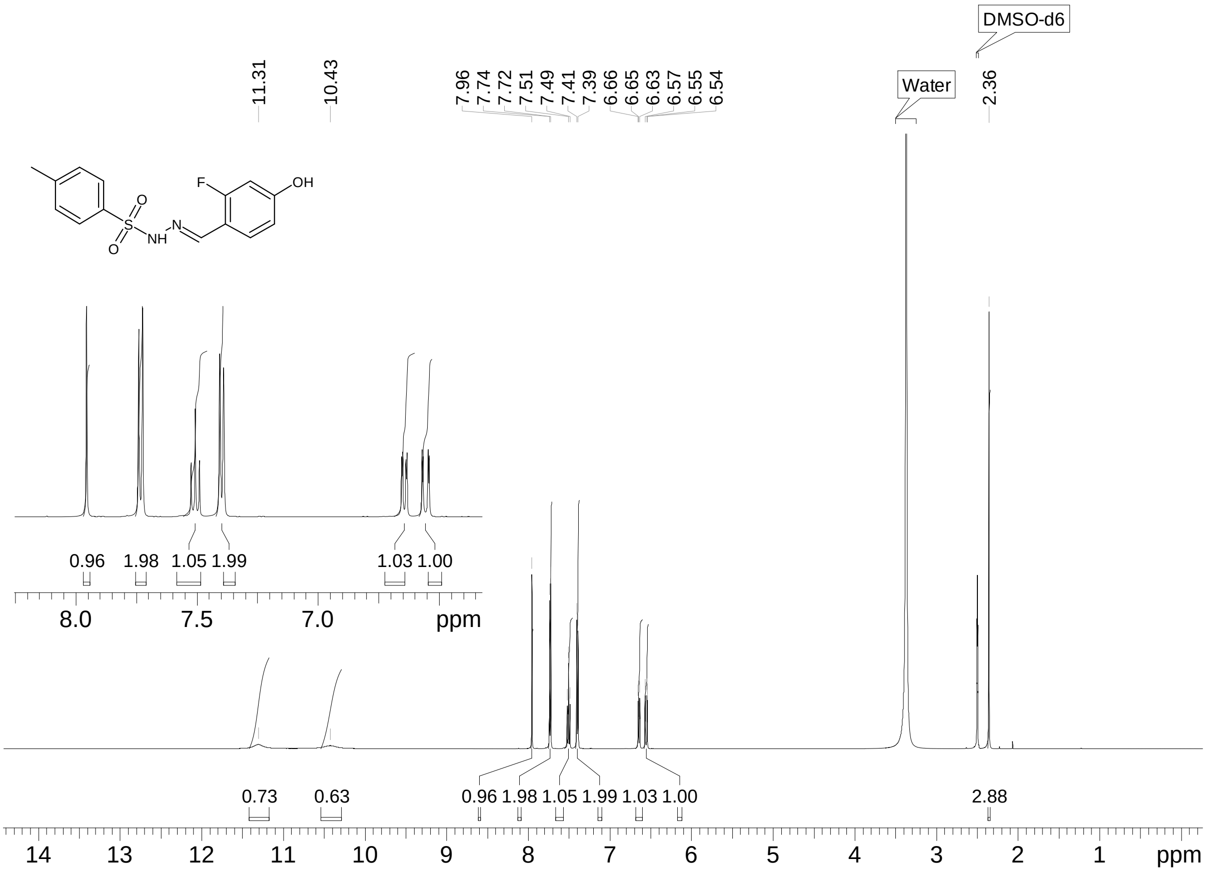

Supplementary Figure 35 ^{1}H spectrum of **12**

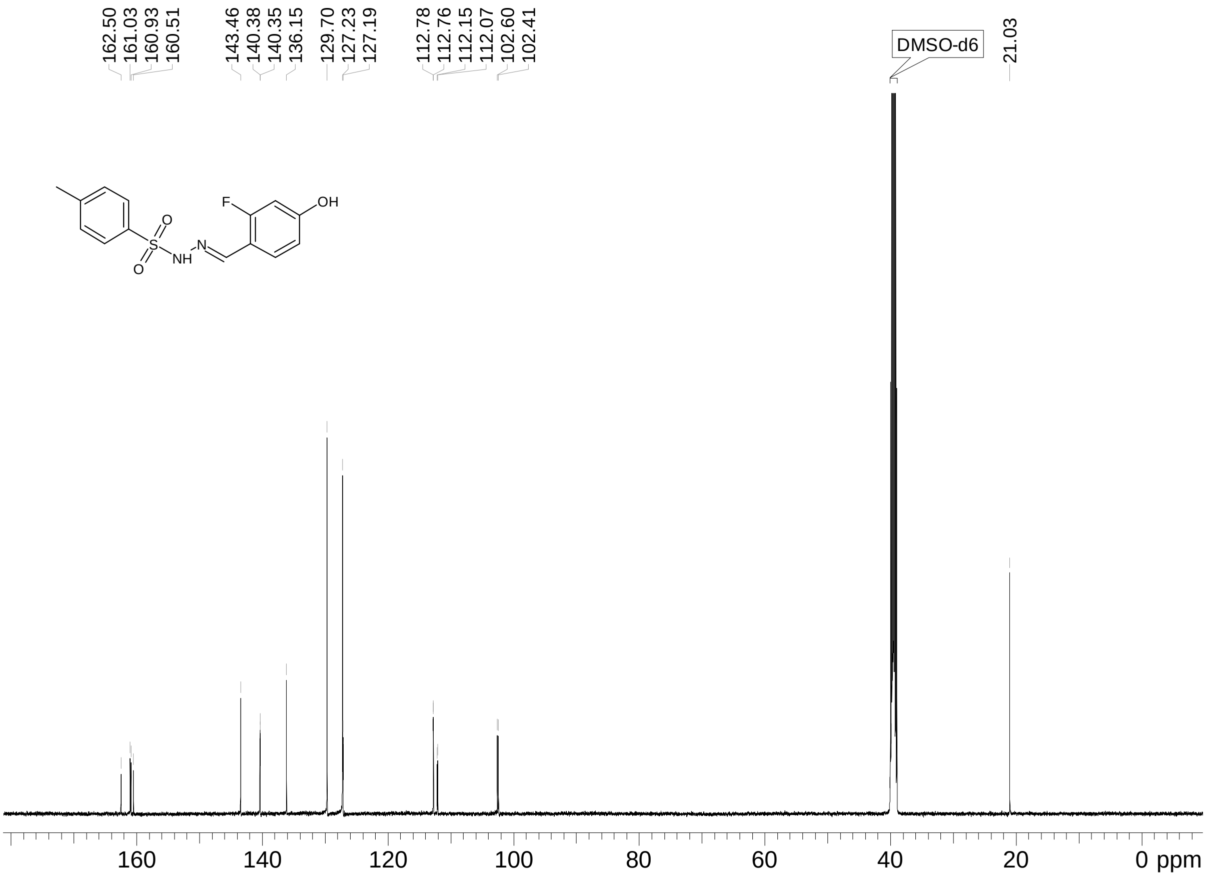

Supplementary Figure 36 ^{13}C spectrum of **12**

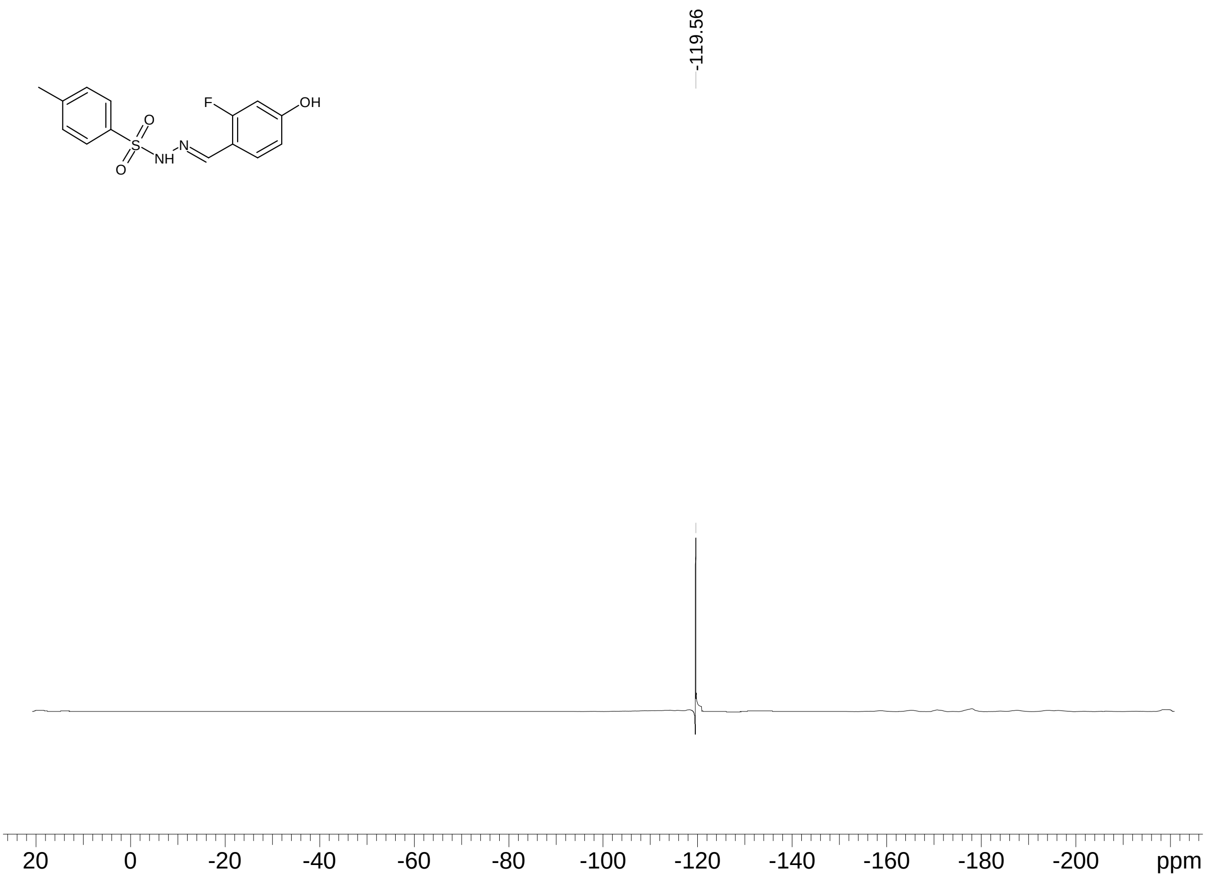

Supplementary Figure 37 ^{19}F spectrum of **12**

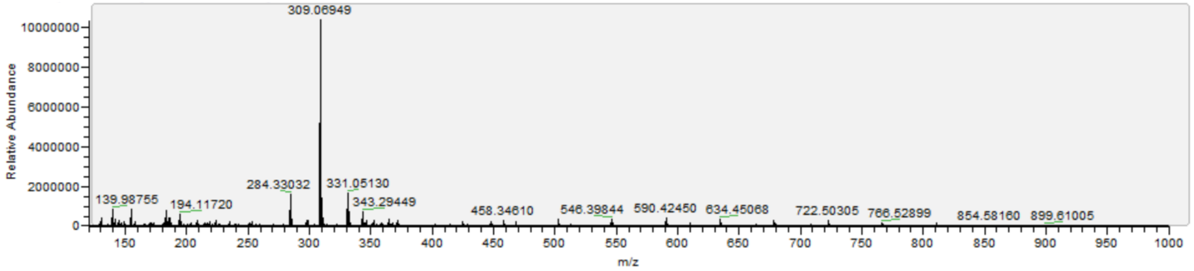

Supplementary Figure 38 HRMS of **12**

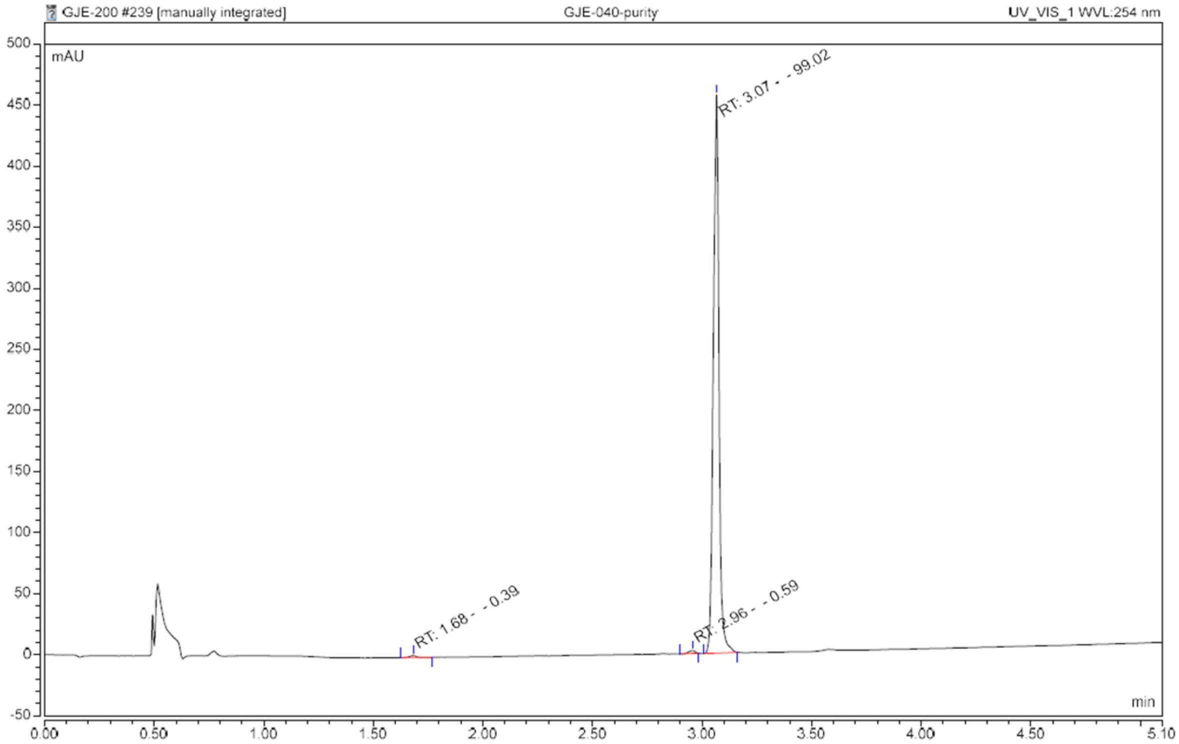

No.	Ret.Time min	Rel.Area %
1	1.682	0.39
2	2.958	0.59
3	3.067	99.02

Supplementary Figure 39 LCMS purity of **12**

Compound 13

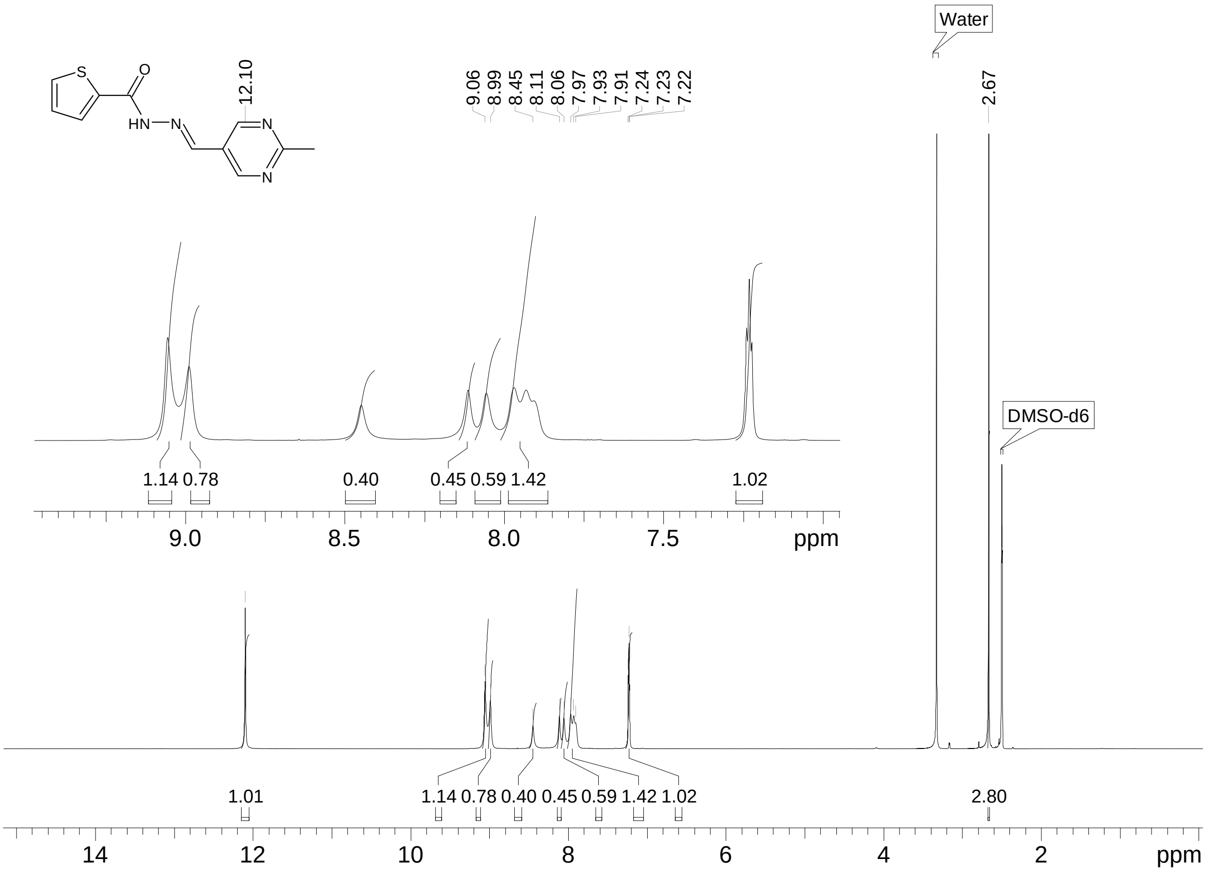

Supplementary Figure 40 ^{1}H spectrum of **13**

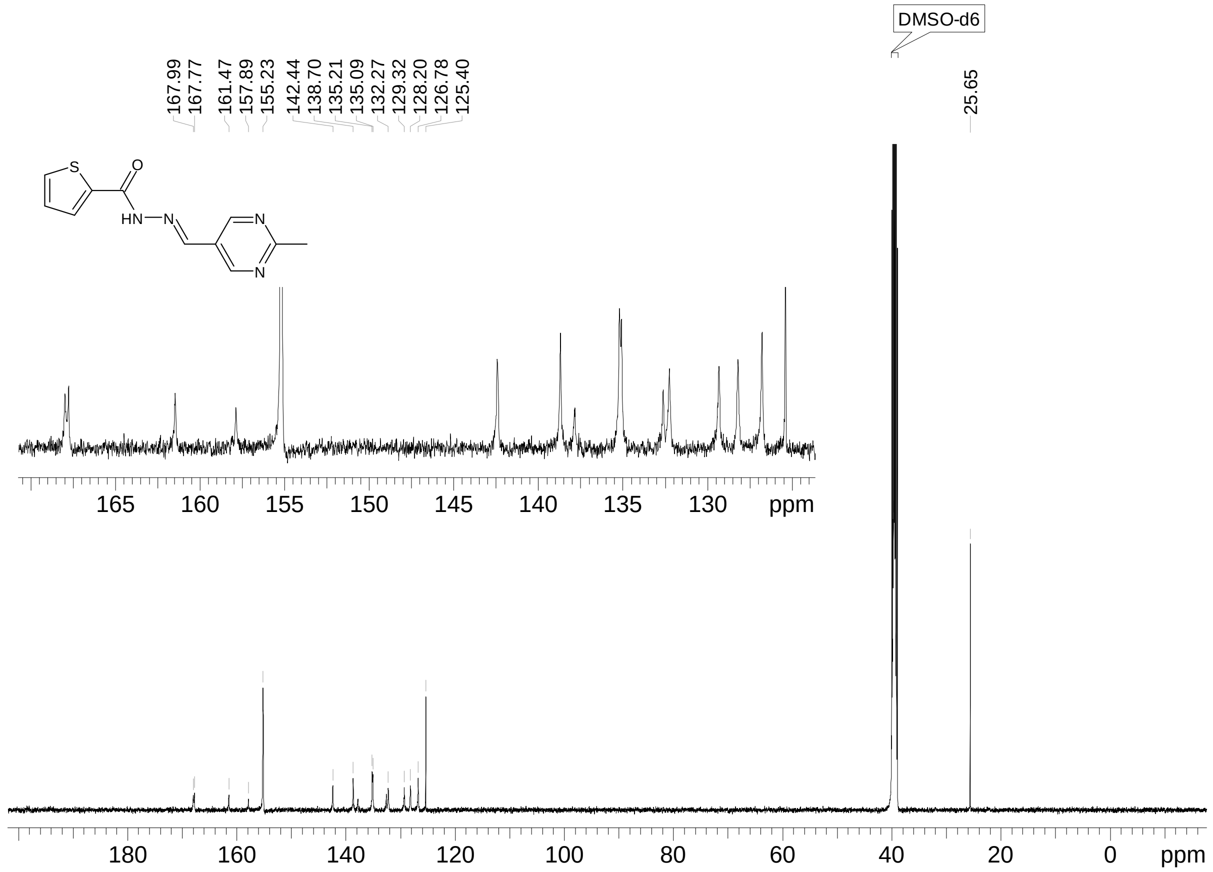

Supplementary Figure 41 ^{13}C spectrum of **13**

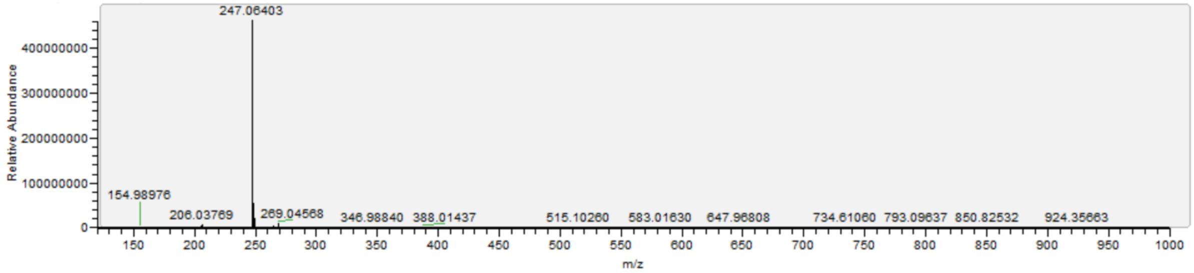

Supplementary Figure 42 HRMS of **13**

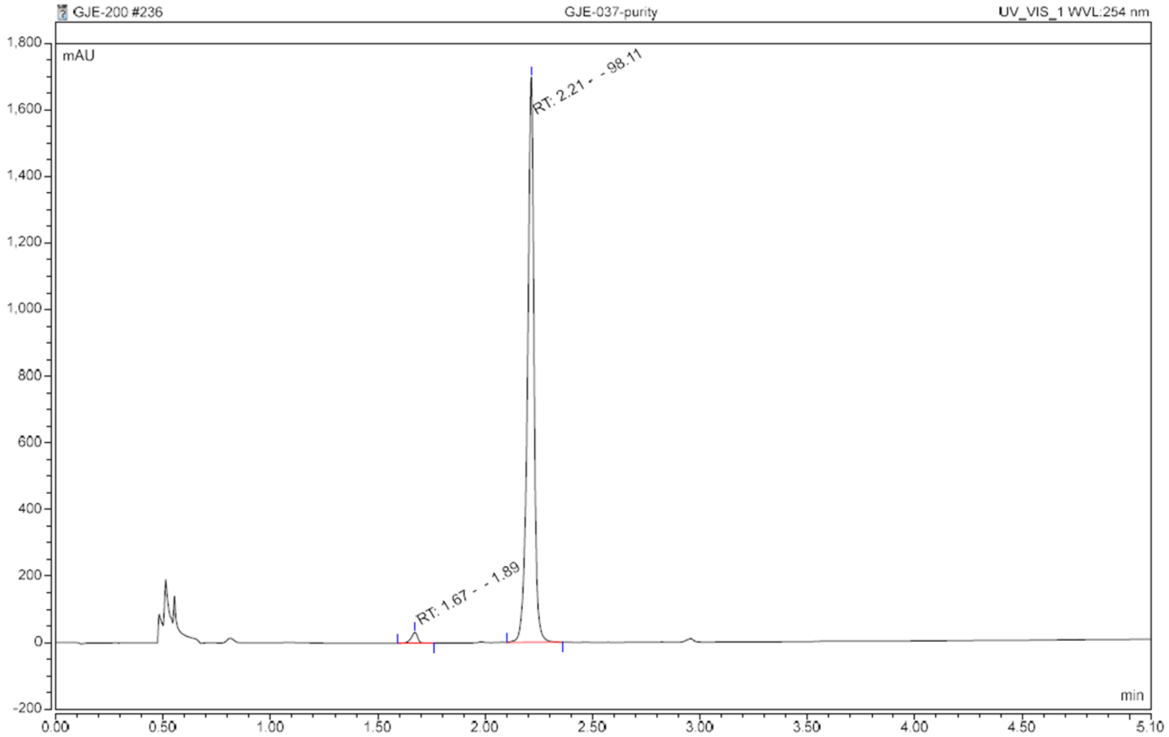

No.	Ret.Time min	Rel.Area %
1	1.673	1.89
2	2.215	98.11

Supplementary Figure 43 LCMS purity of **13**

Compound 14

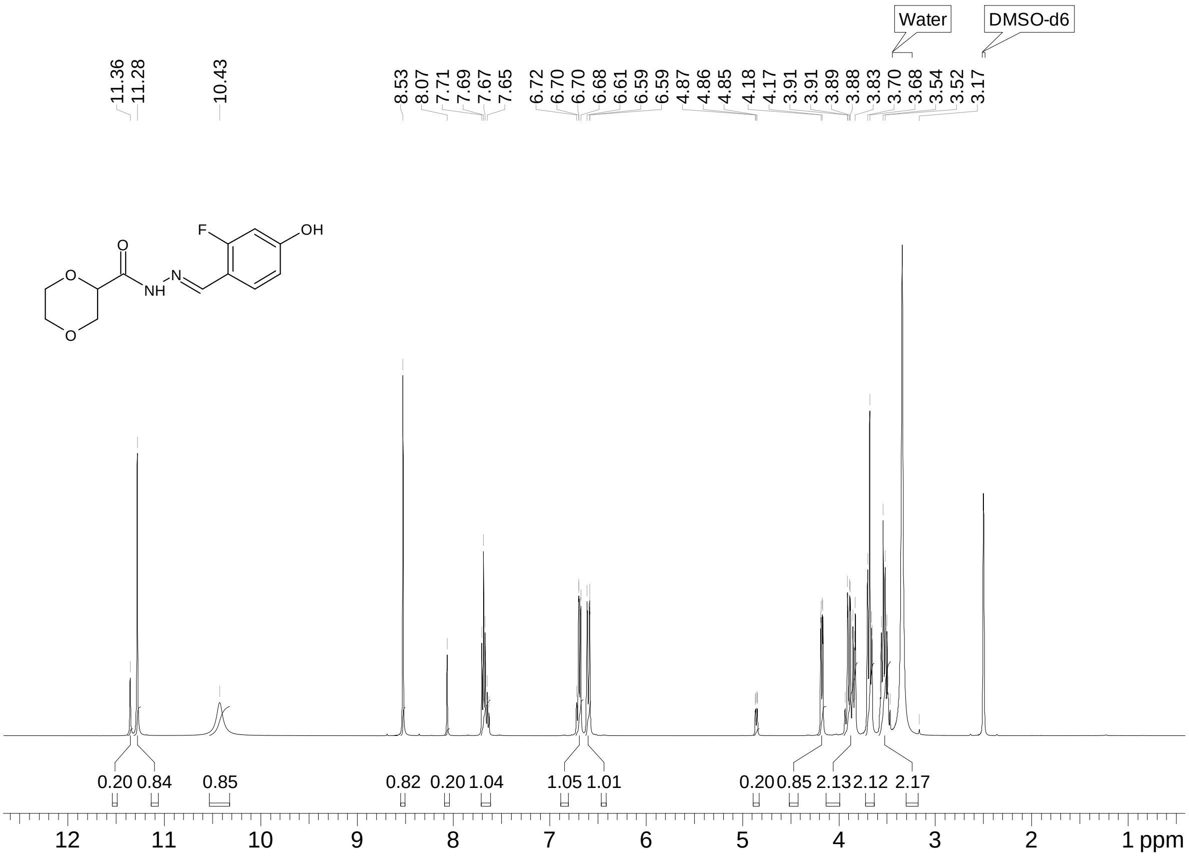

Supplementary Figure 44 ^{1}H spectrum of **14**

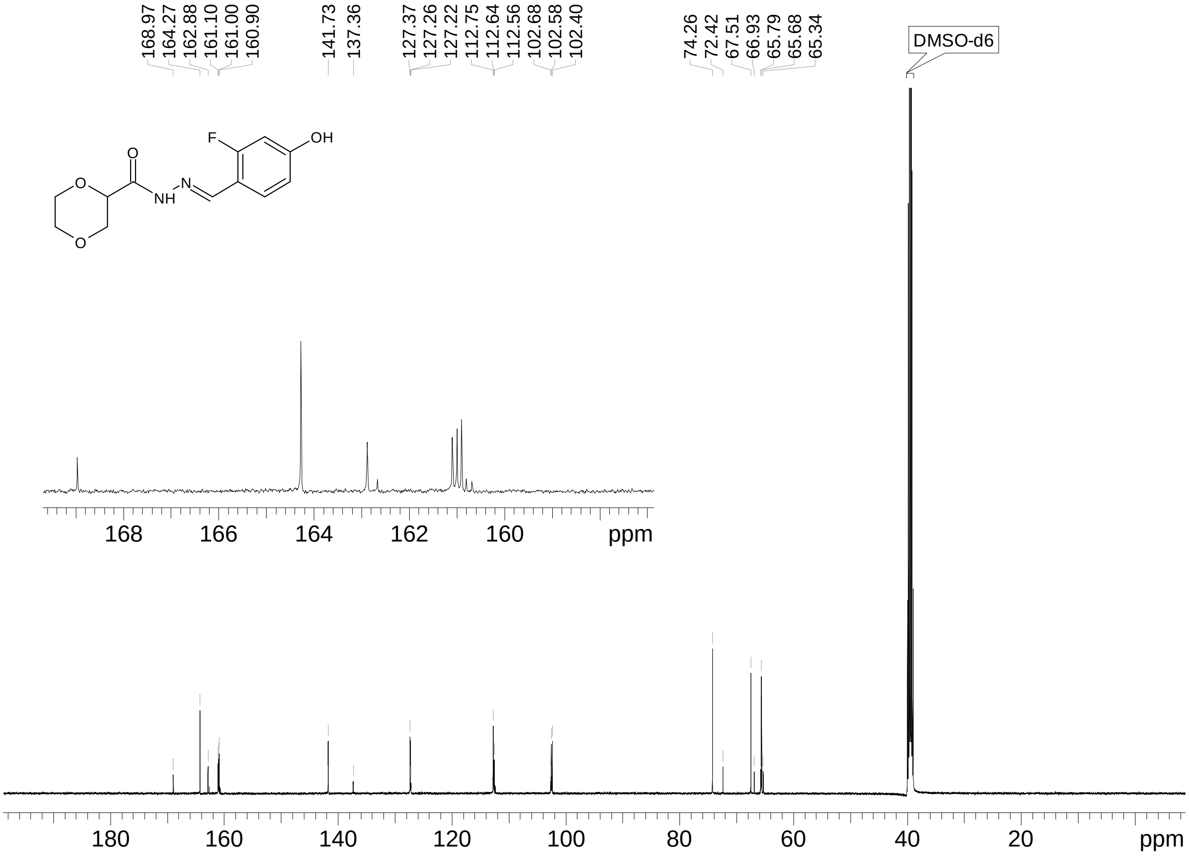

Supplementary Figure 45 ^{13}C spectrum of **14**

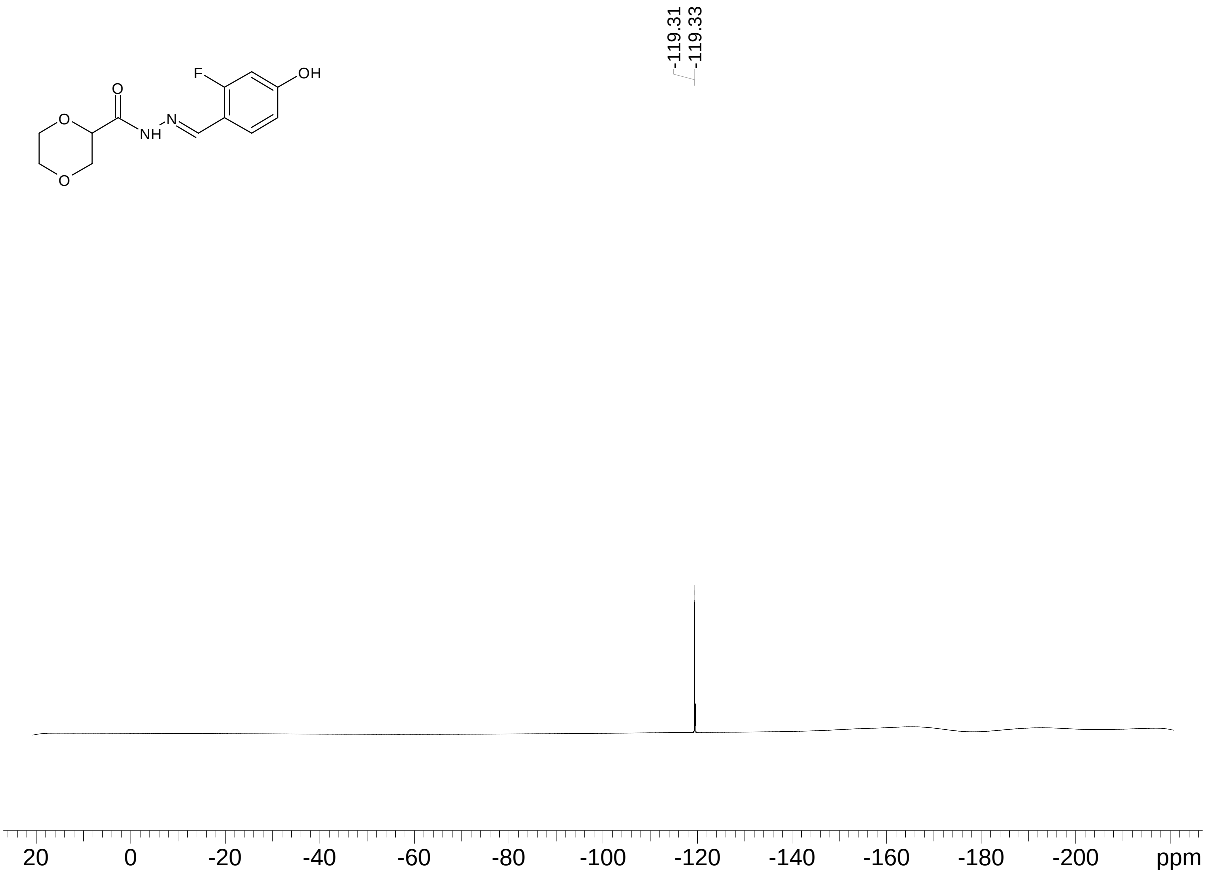

Supplementary Figure 46 ^{19}F spectrum of **14**

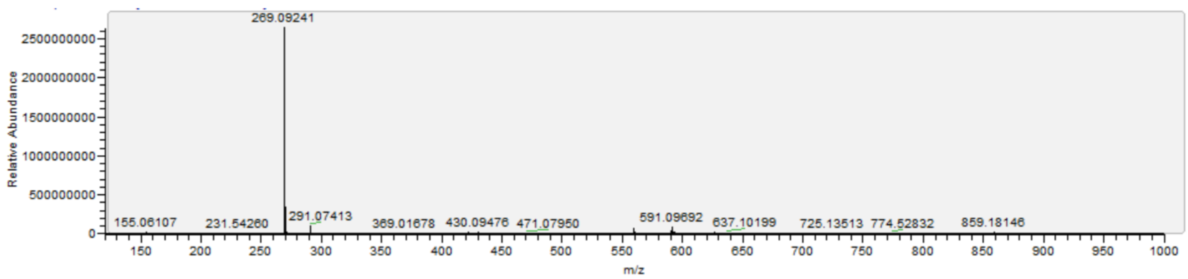

Supplementary Figure 47 HRMS of **14**

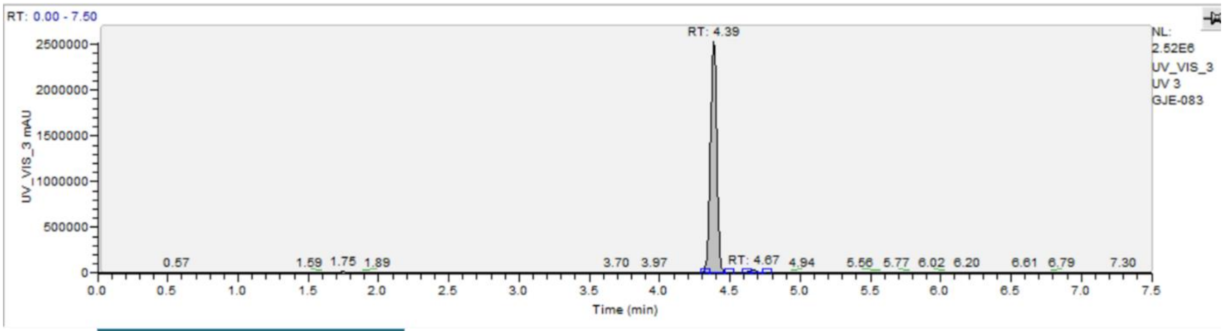

No	Ret. Time min	Rel. Area %
1	4.39	98.39
2	4.39	1.61

Supplementary Figure 48 LCMS purity of **14**

Compound 15

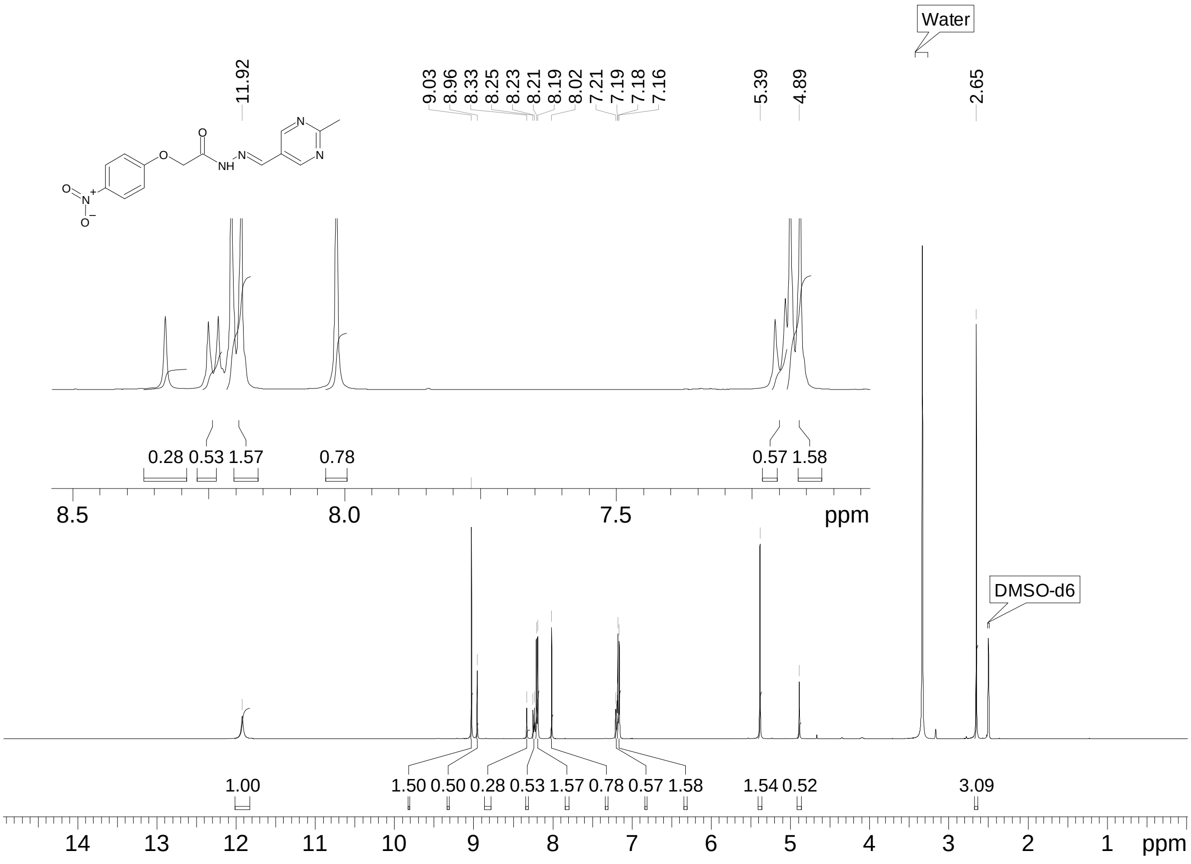

Supplementary Figure 49 ^{1}H spectrum of **15**

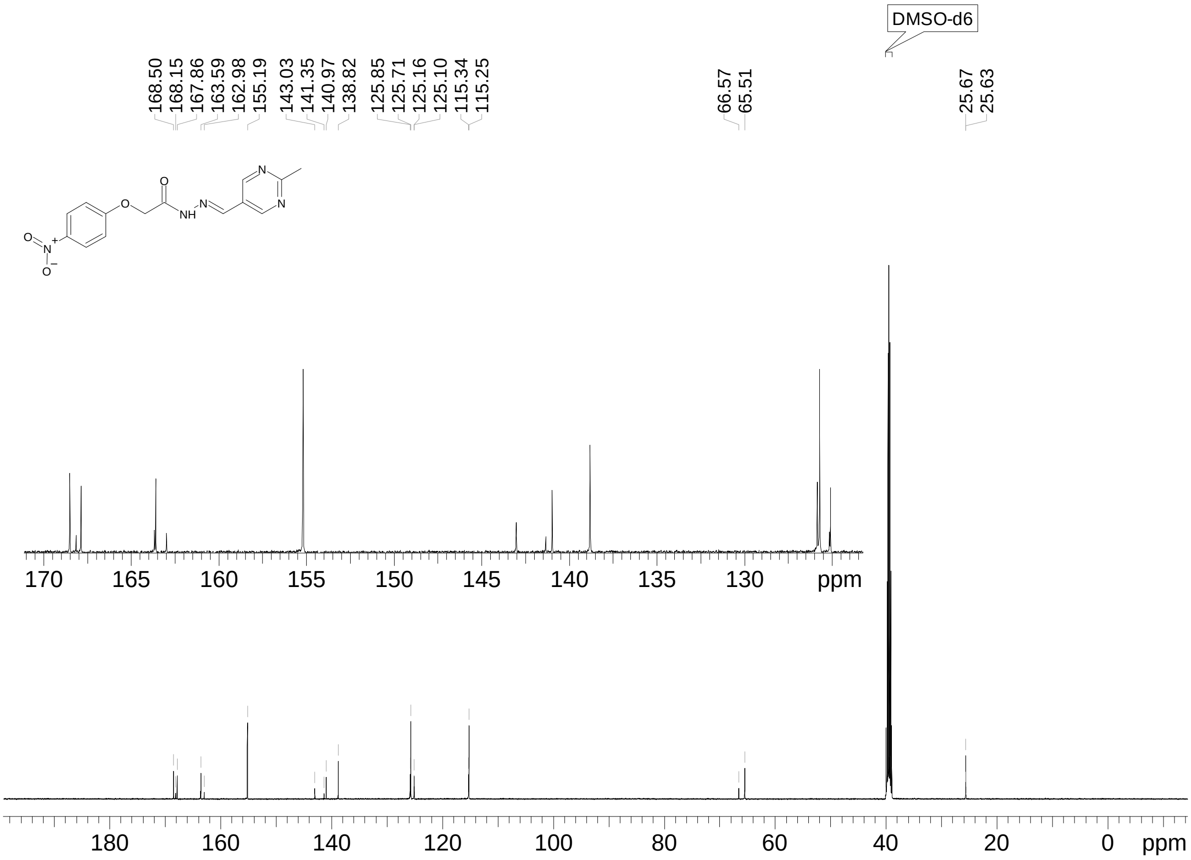

Supplementary Figure 50 ^{13}C spectrum of **15**

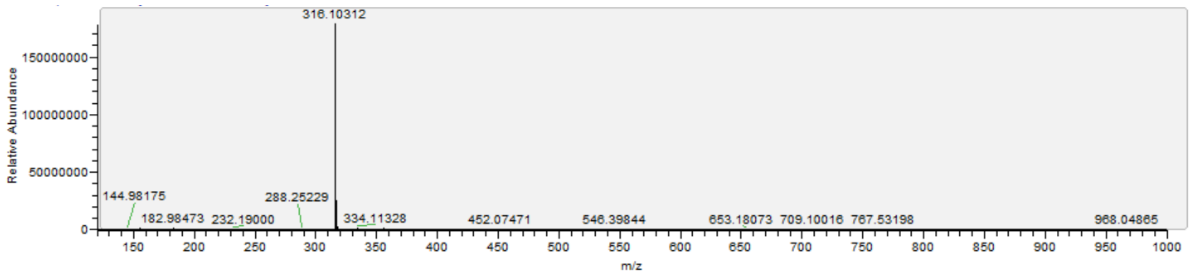

Supplementary Figure 51 HRMS of **15**

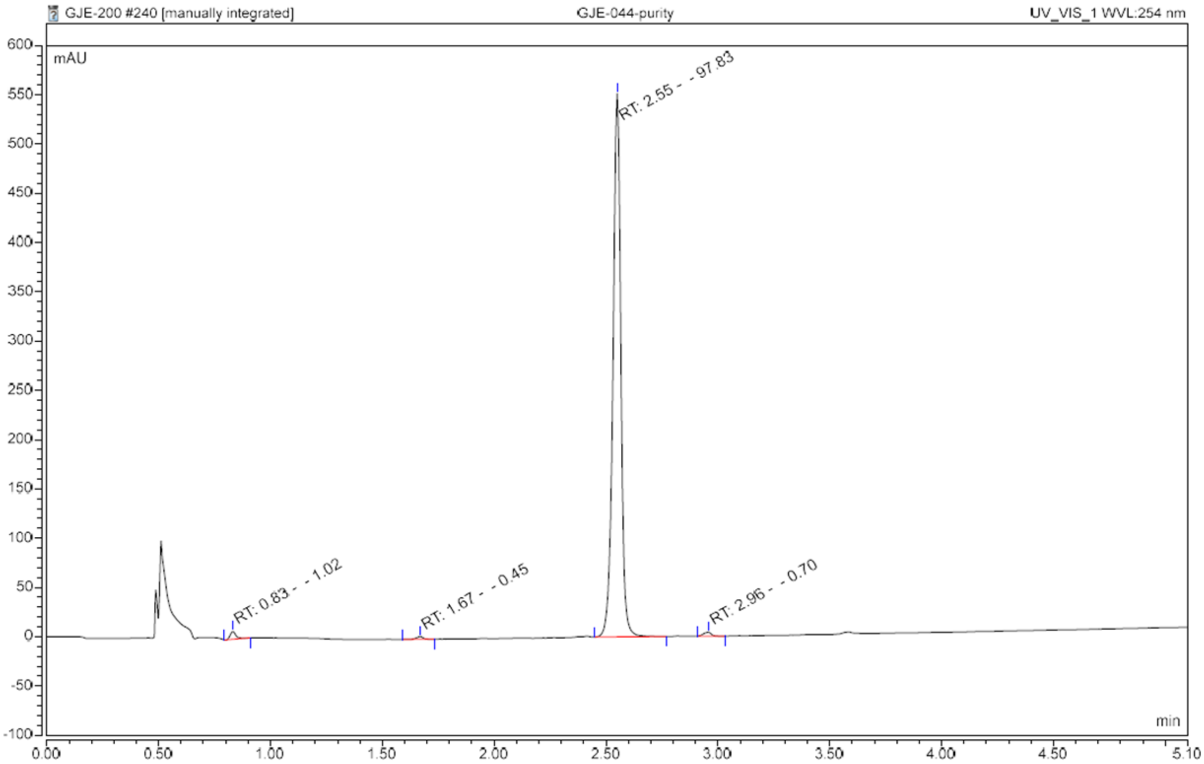

No.	Ret.Time min	Rel.Area %
1	0.831	1.02
2	1.668	0.45
3	2.550	97.83
4	2.958	0.70

Supplementary Figure 52 LCMS purity of **15**

Compound 16

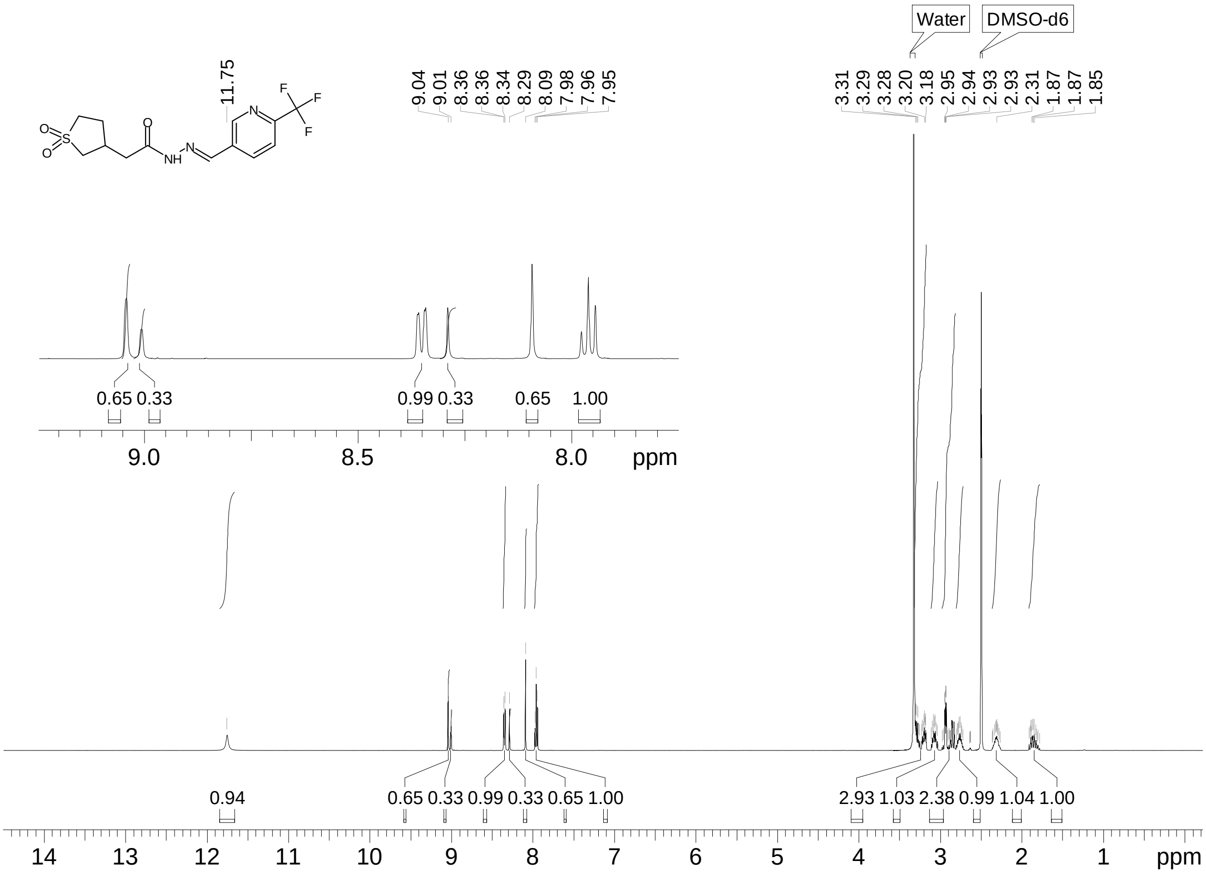

Supplementary Figure 53 ^{1}H spectrum of **16**

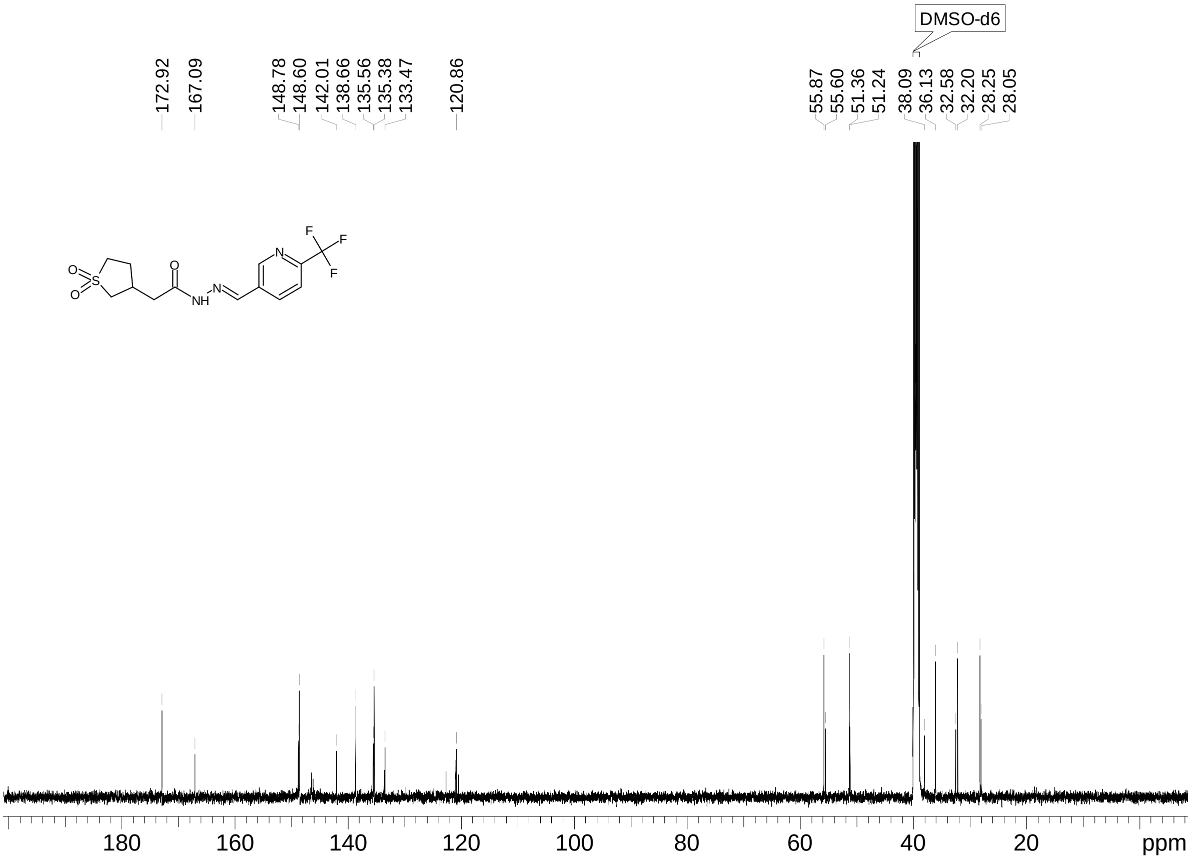

Supplementary Figure 54 ^{13}C spectrum of **16**

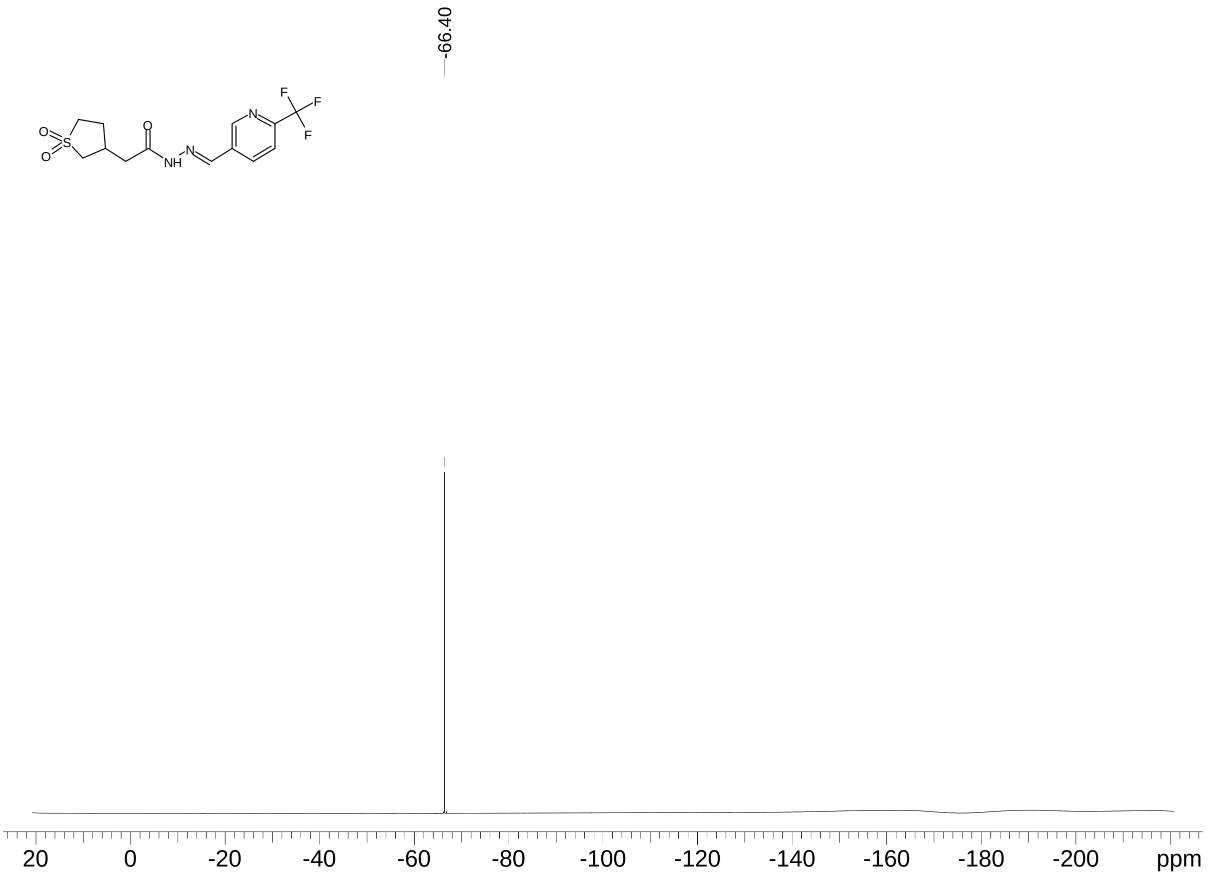

Supplementary Figure 55 ^{19}F spectrum of **16**

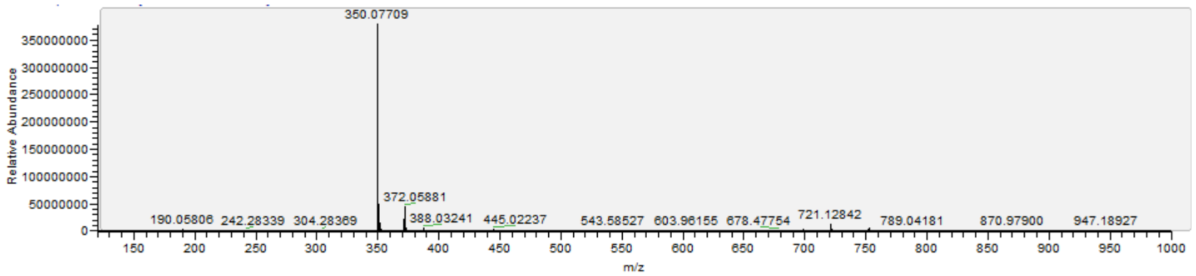

Supplementary Figure 56 HRMS of **16**

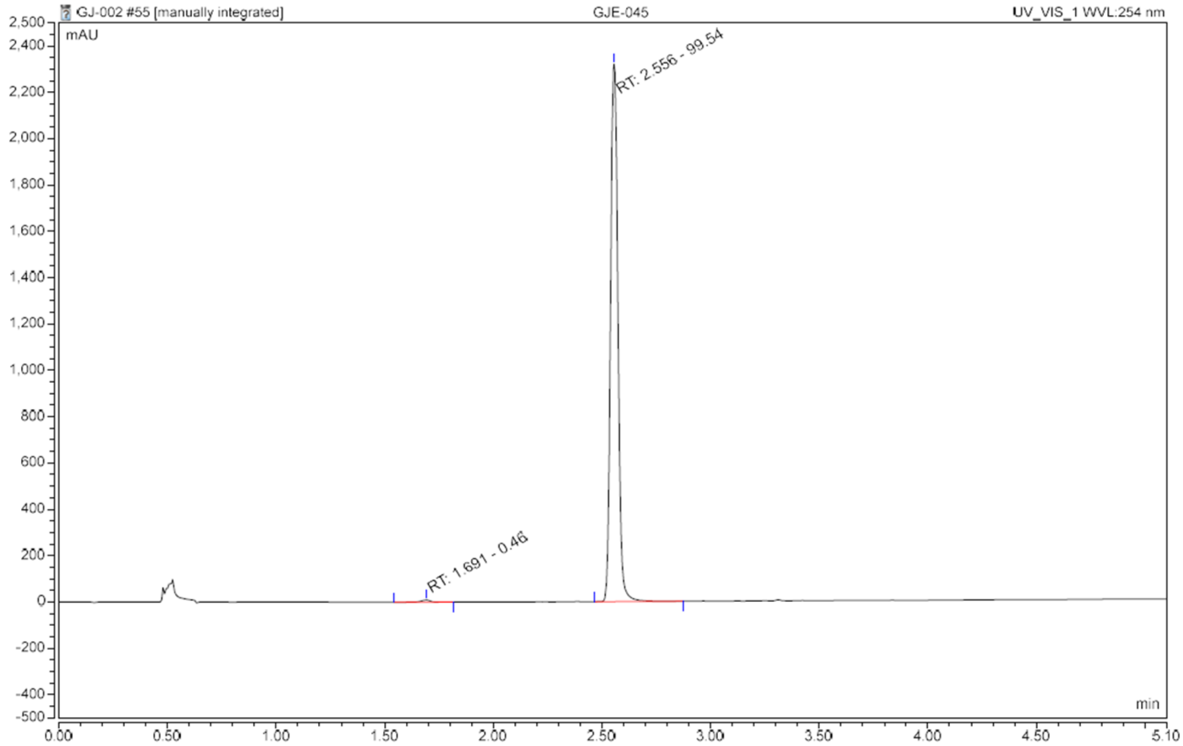

No.	Ret.Time min	Rel.Area %
1	1.691	0.46
2	2.556	99.54

Supplementary Figure 57 LCMS purity of **16**

Native MS spectra

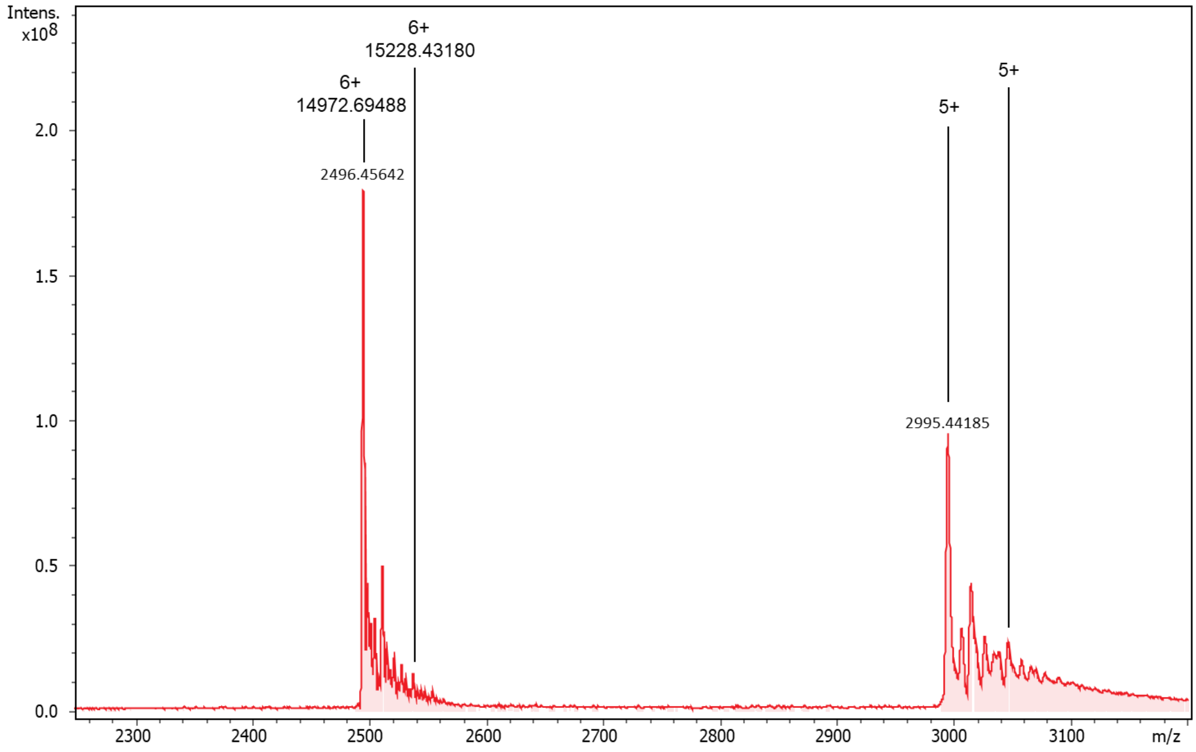

Supplementary Figure 58 Native MS spectrum of Nsp10 with **1**

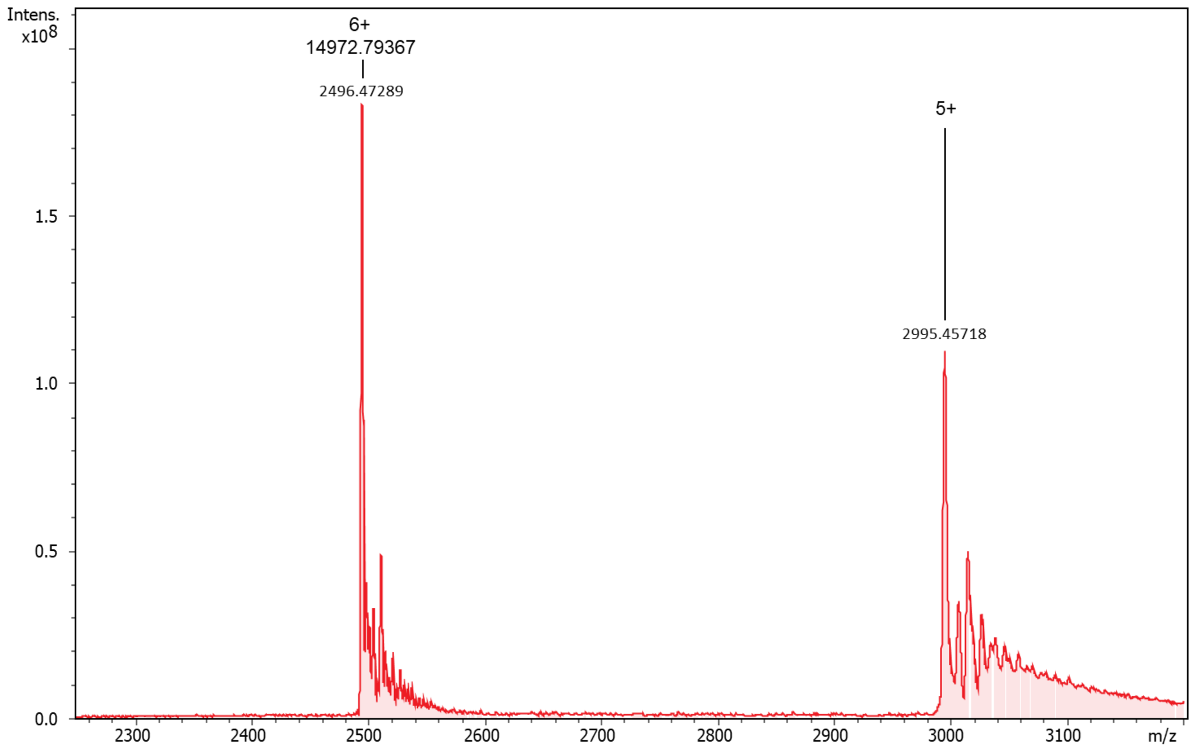

Supplementary Figure 59 Native MS spectrum of Nsp10 with **2**

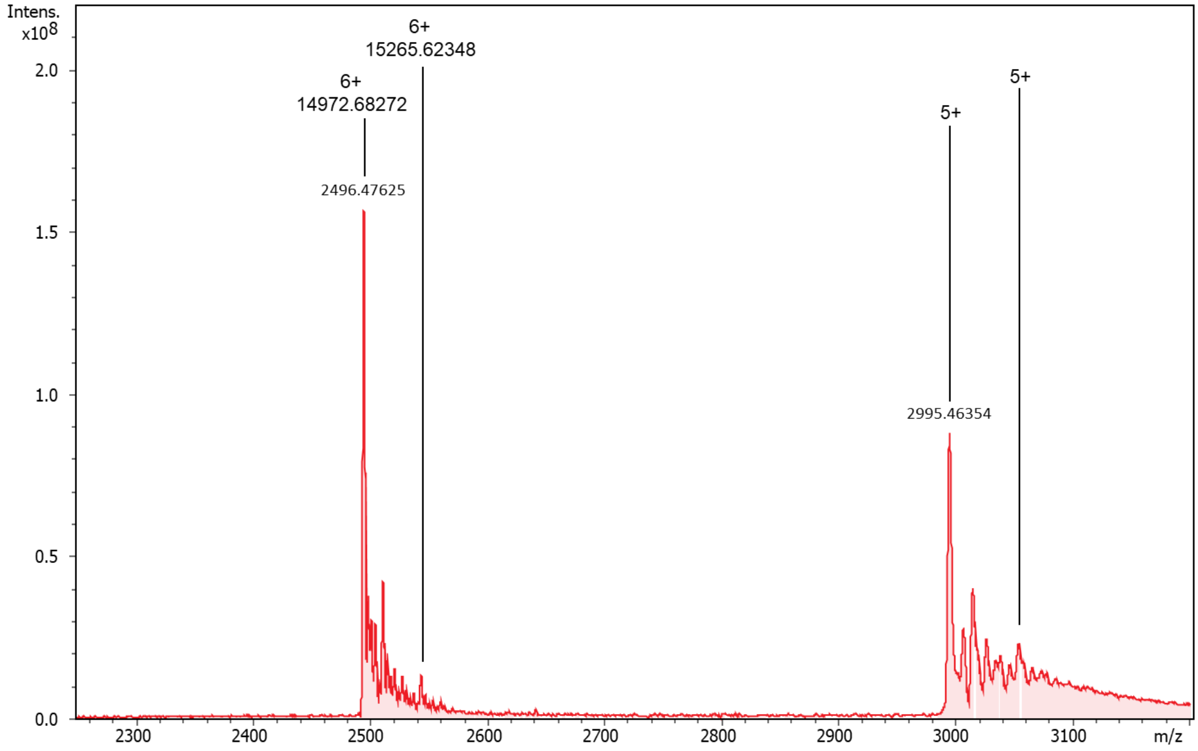

Supplementary Figure 60 Native MS spectrum of Nsp10 with **3**

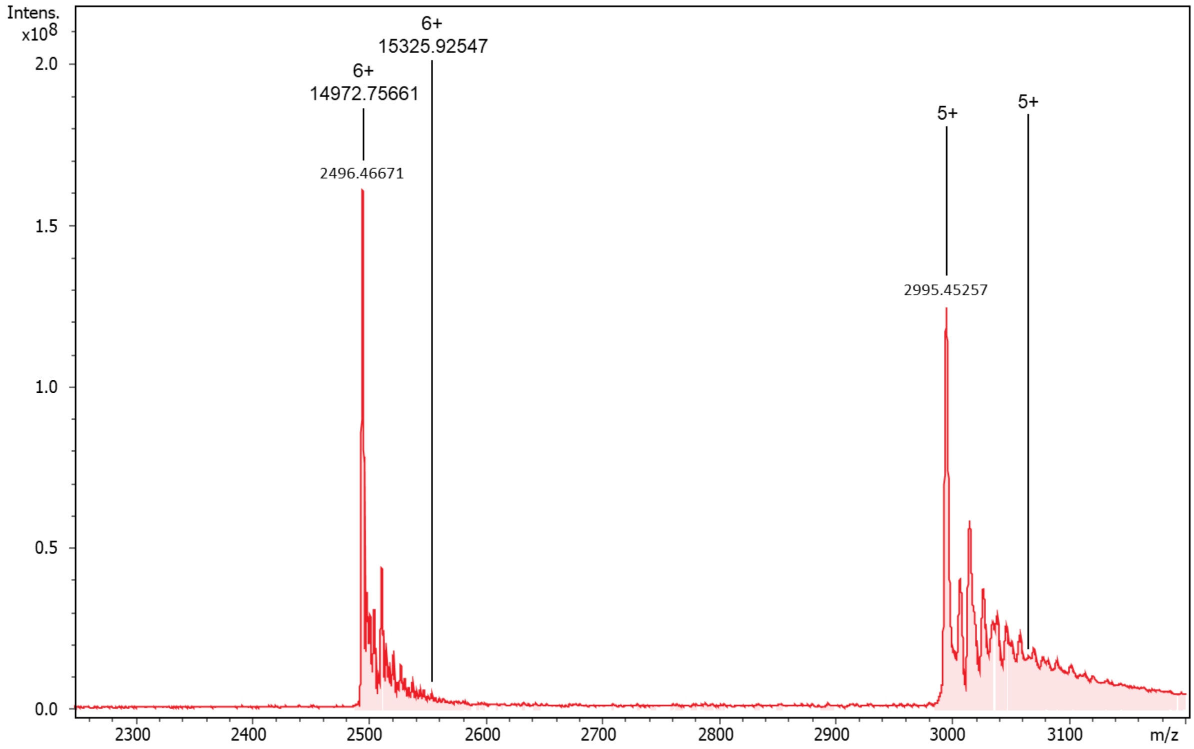

Supplementary Figure 61 Native MS spectrum of Nsp10 with **4**

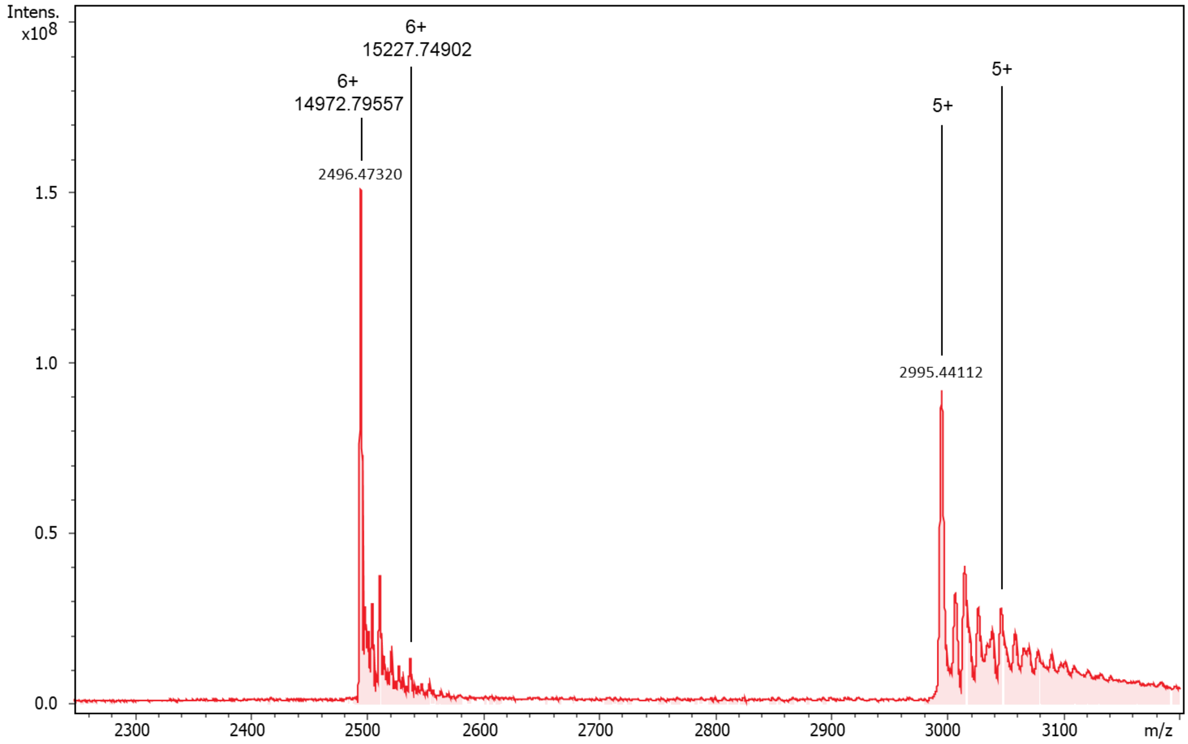

Supplementary Figure 62 Native MS spectrum of Nsp10 with **5**

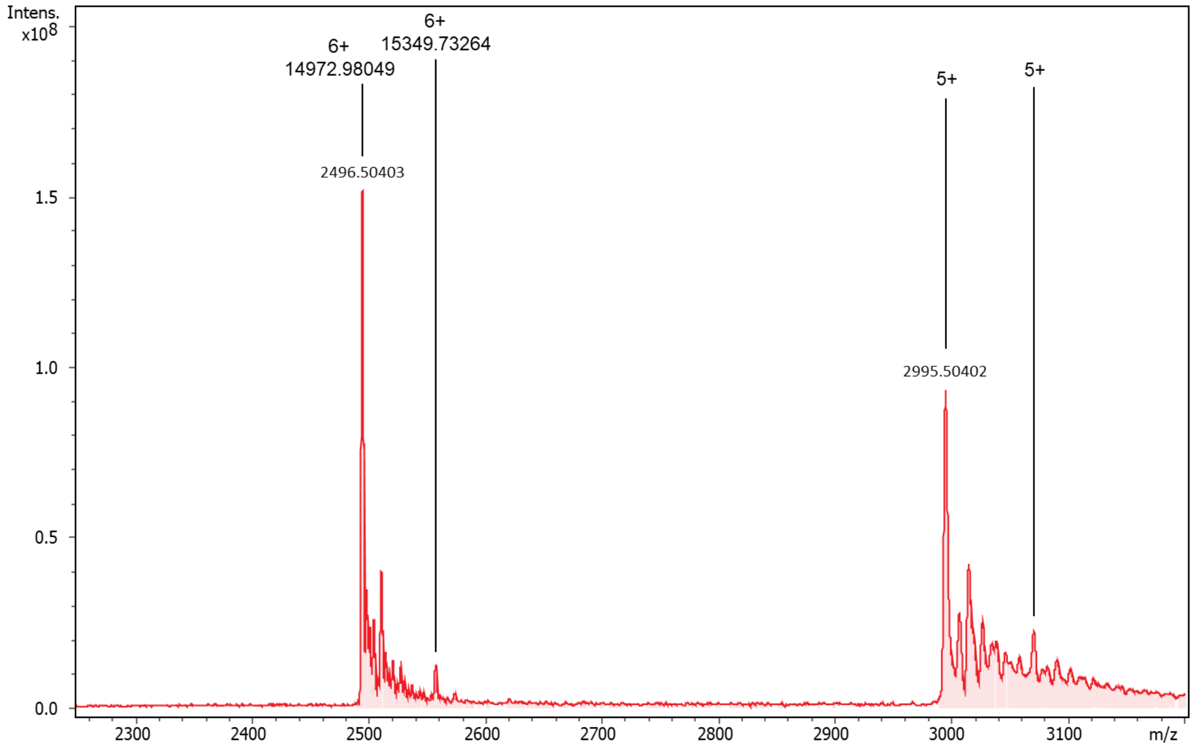

Supplementary Figure 63 Native MS spectrum of Nsp10 with **6**

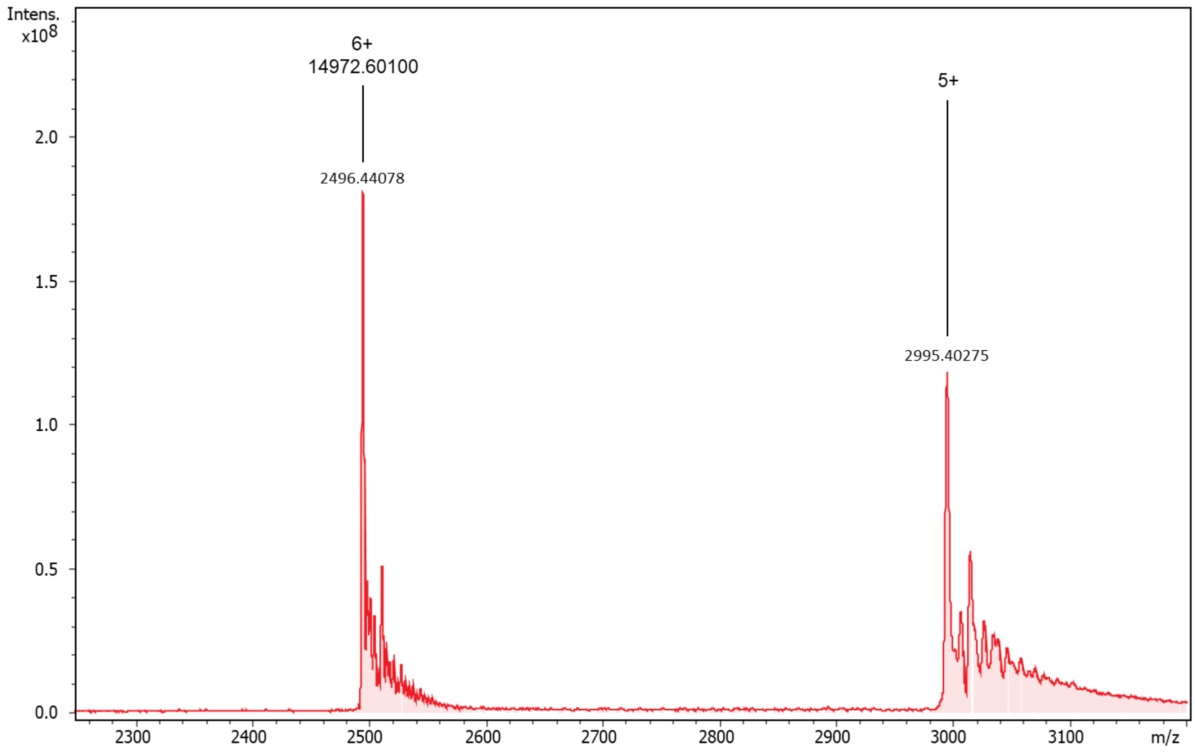

Supplementary Figure 64 Native MS spectrum of Nsp10 with **7**

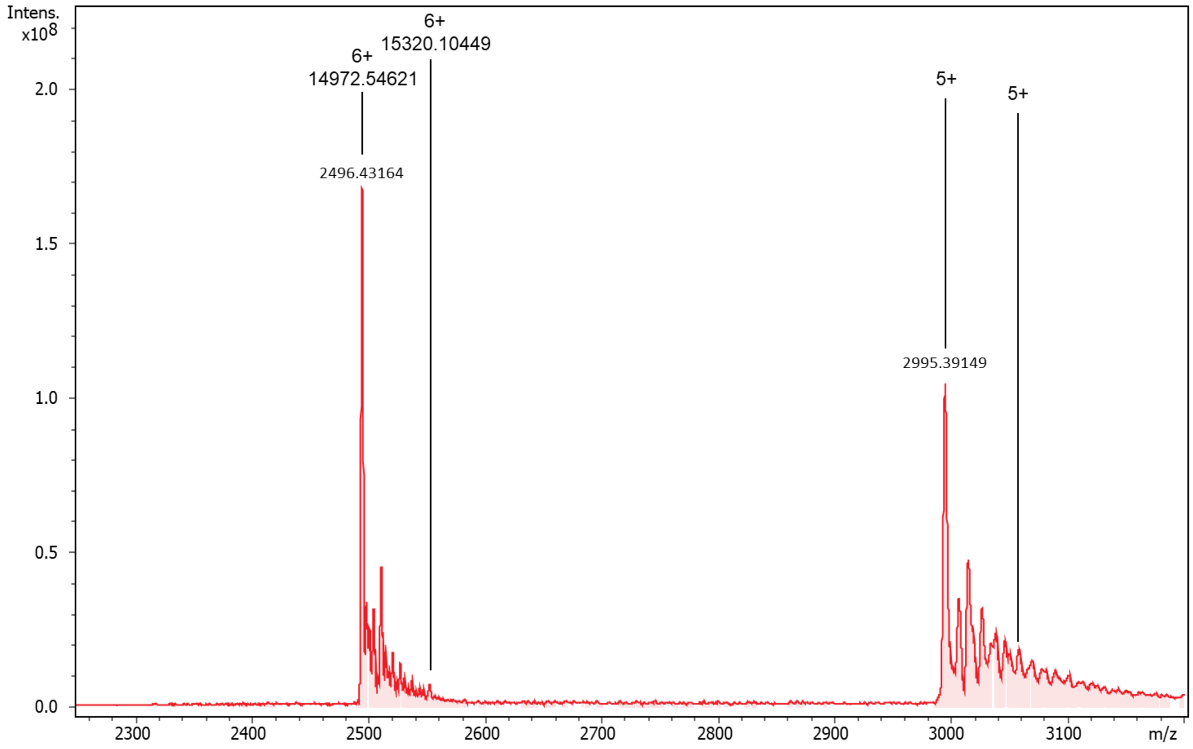

Supplementary Figure 65 Native MS spectrum of Nsp10 with **8**

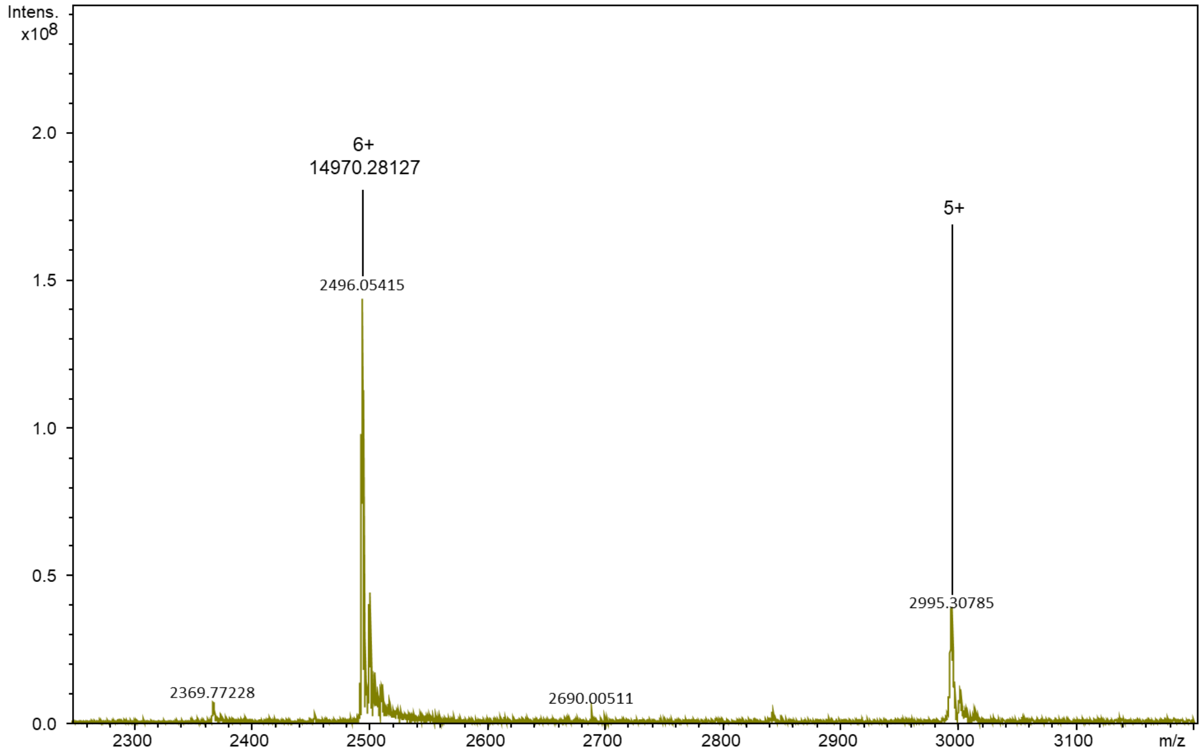

Supplementary Figure 66 Native MS spectrum of Nsp10 with **9**

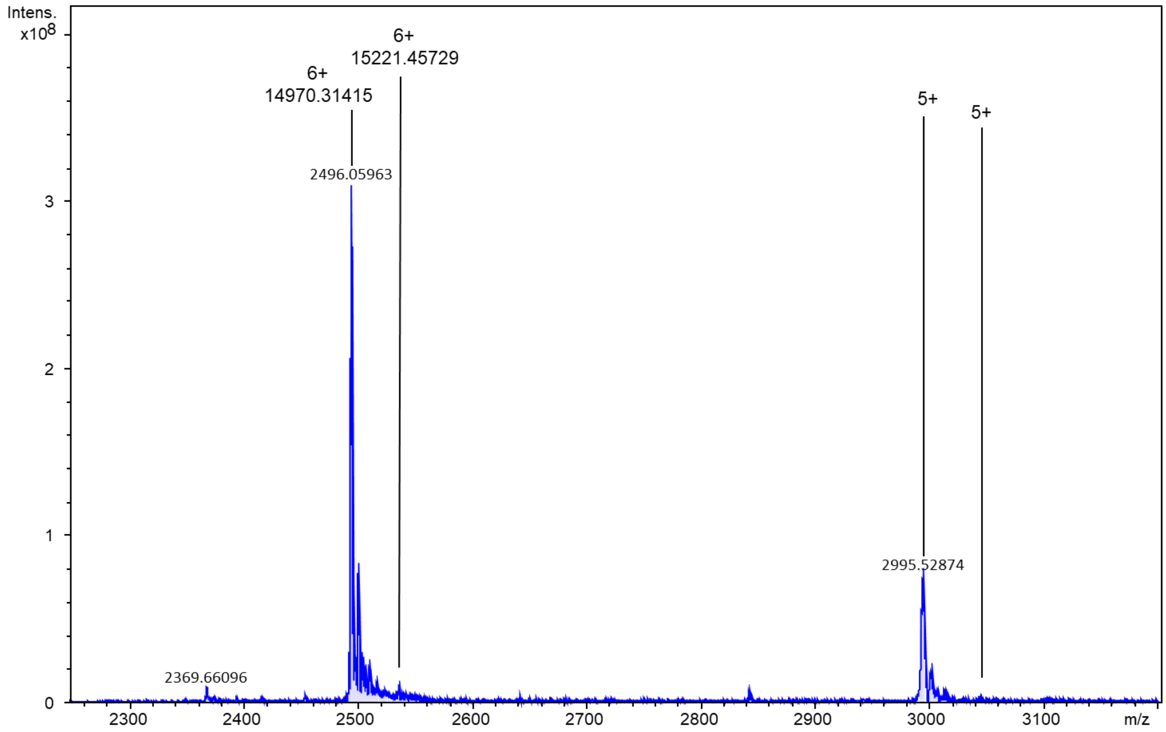

Supplementary Figure 67 Native MS spectrum of Nsp10 with **10**

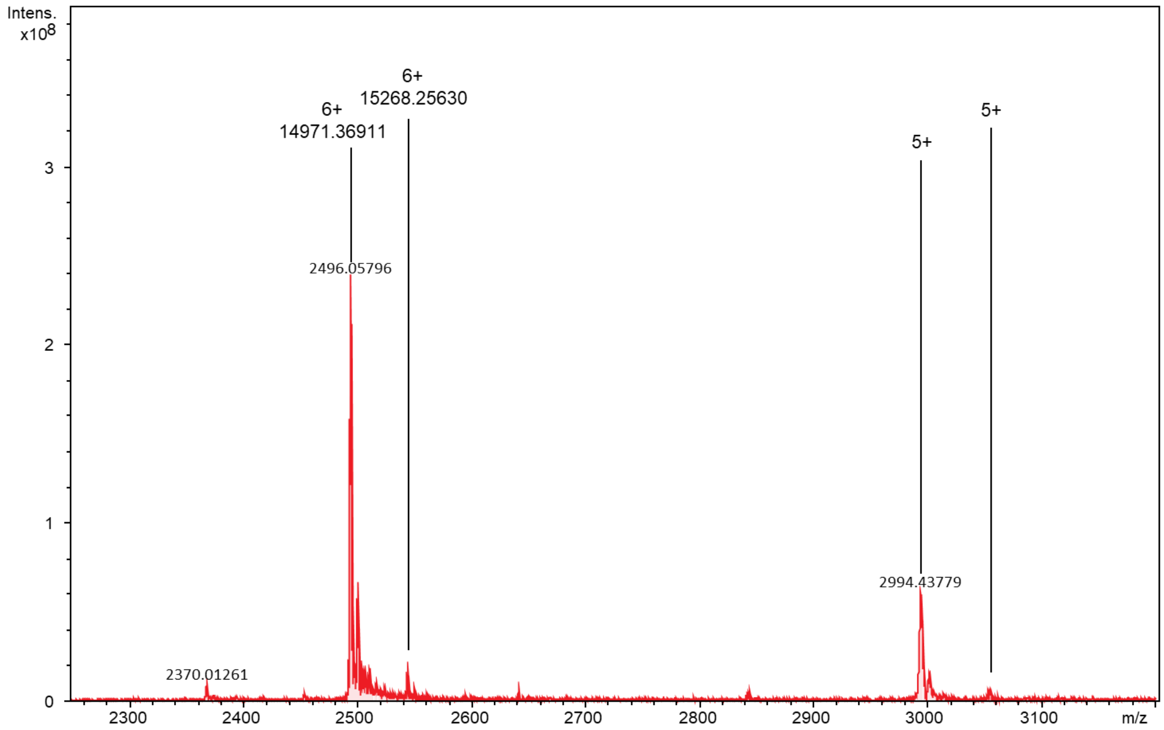

Supplementary Figure 68 Native MS spectrum of Nsp10 with **11**

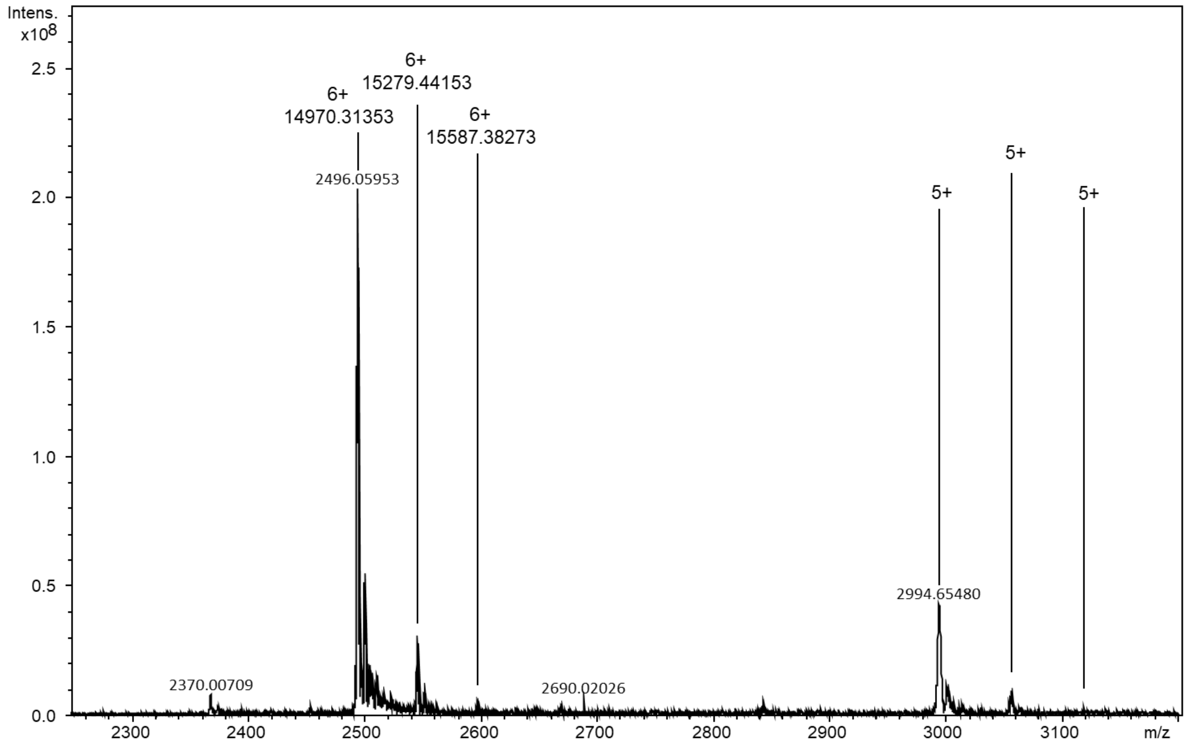

Supplementary Figure 69 Native MS spectrum of Nsp10 with **12**

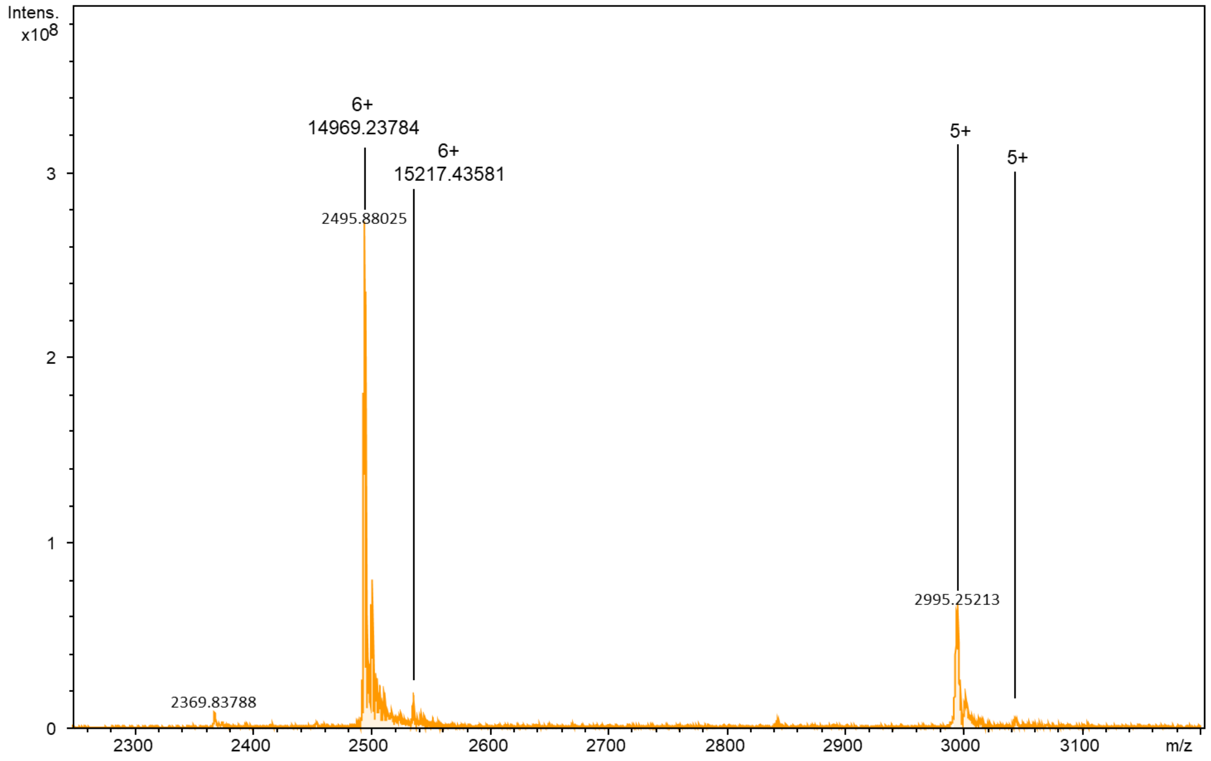

Supplementary Figure 70 Native MS spectrum of Nsp10 with **13**

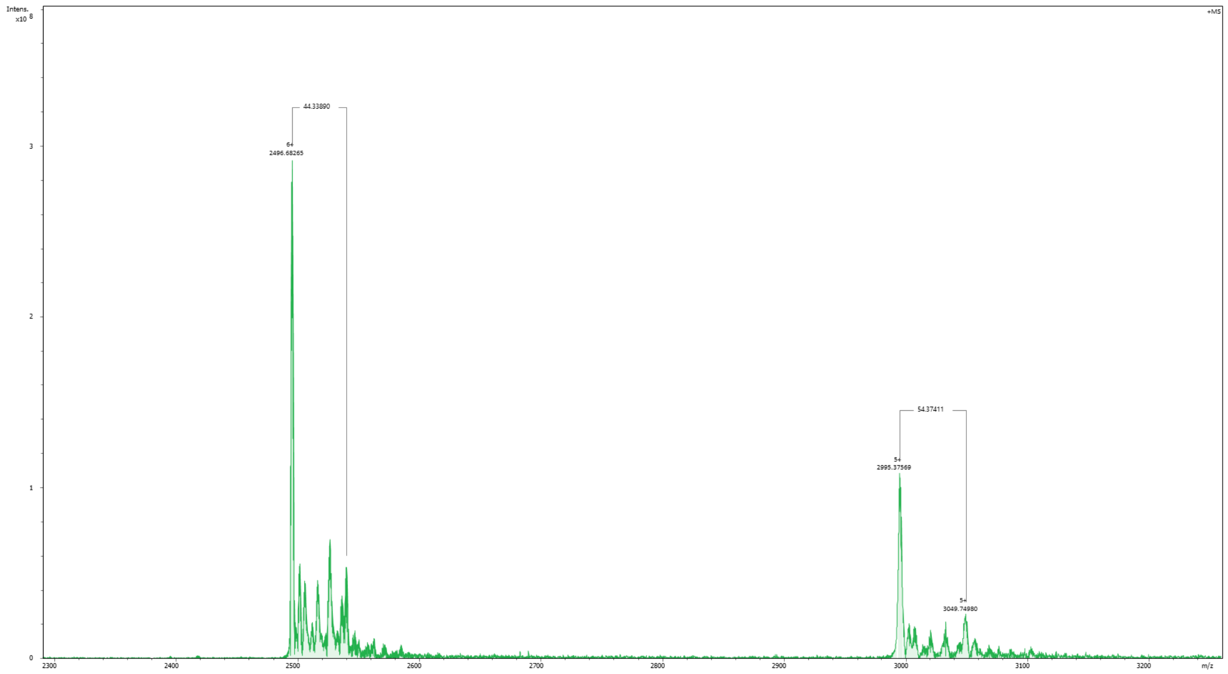

Supplementary Figure 71 Native MS spectrum of Nsp10 with **14**

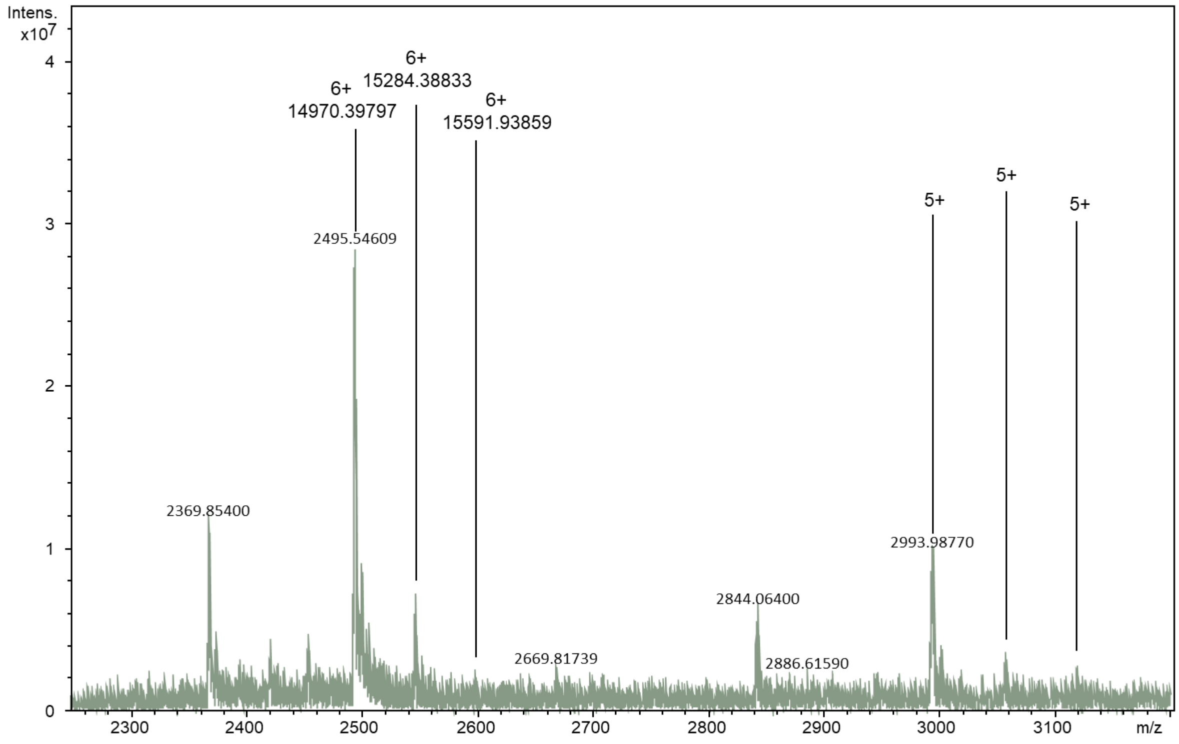

Supplementary Figure 72 Native MS spectrum of Nsp10 with **15**

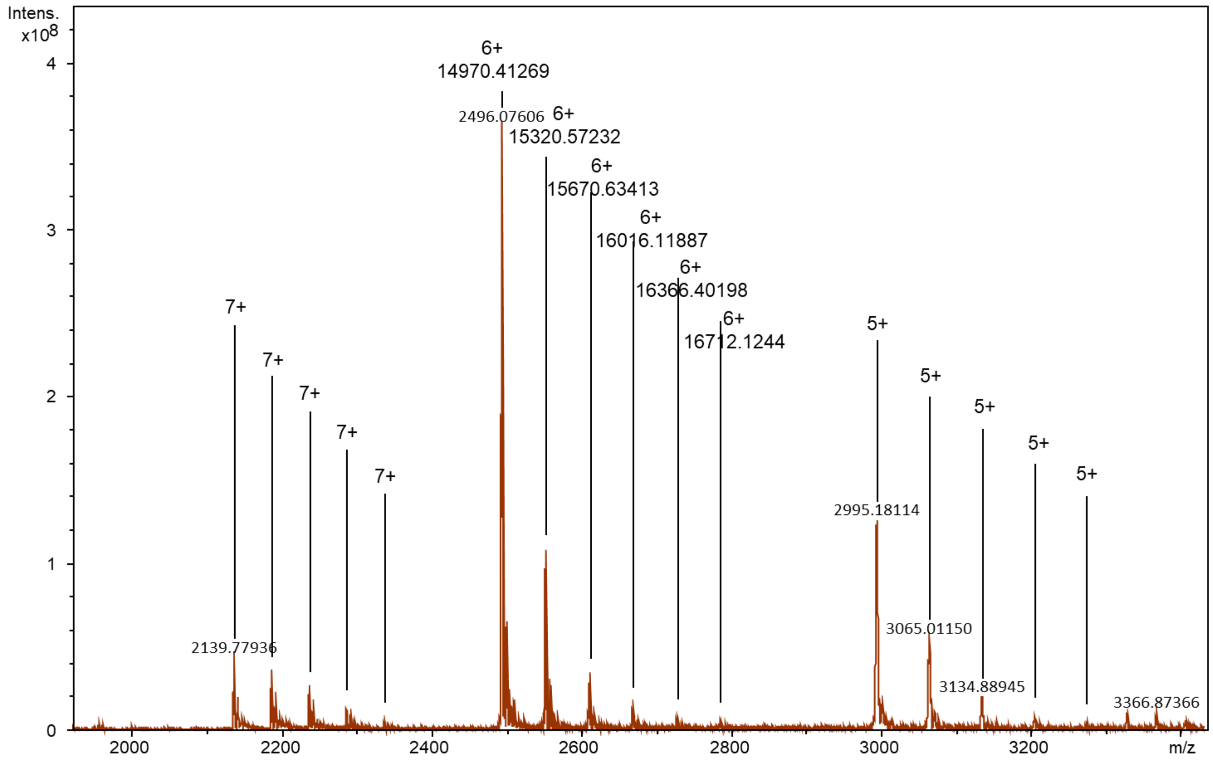

Supplementary Figure 73 Native MS spectrum of Nsp10 with **16**

SPR sensorgrams

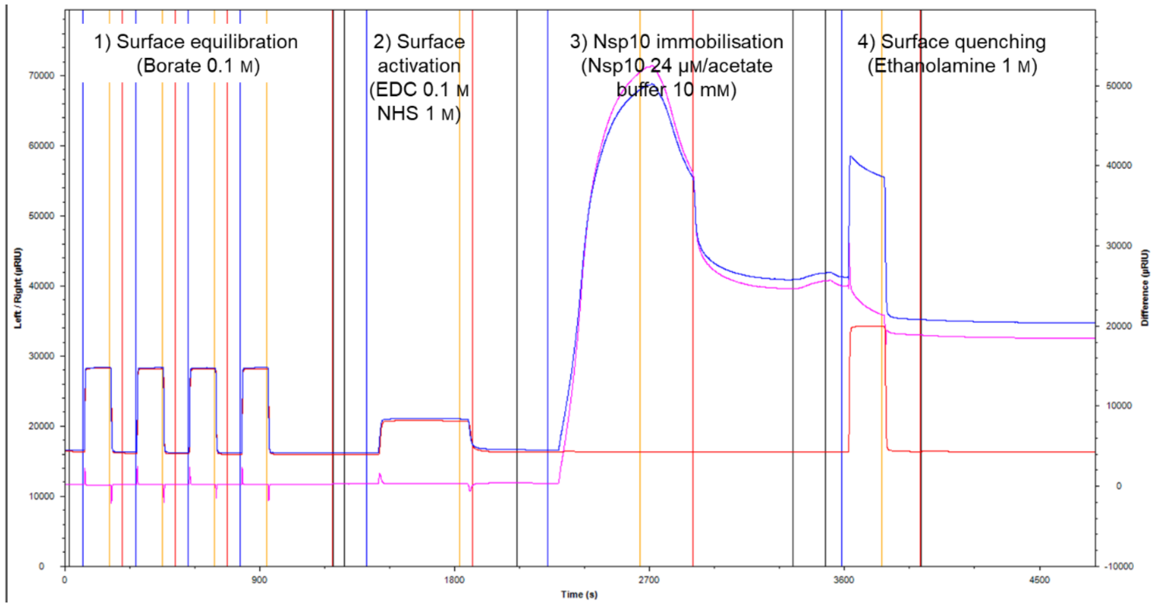

Supplementary Figure 74 Sensorgram of the immobilization procedure for Nsp10 on CMD500M sensor chip: (1) Four injections of cleaning solution, (2) activation solution, (3) Nsp10, and (4) quenching solution. The blue, red, and magenta curves represent the left (active) channel, right (reference) channel, and the difference, respectively.

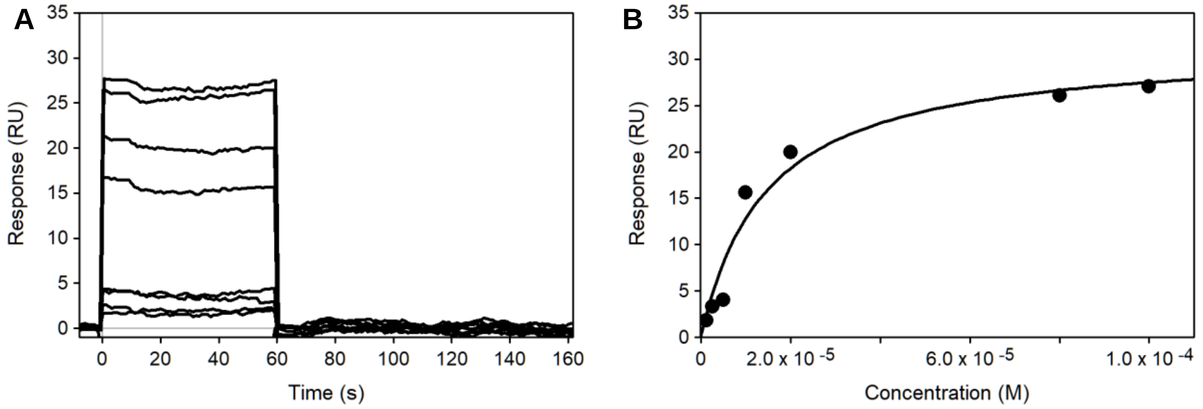

Supplementary Figure 75 (A) Overlay of sensorgrams of **1** injected at concentrations 1.25−100 μM over an immobilised Nsp10; (B) Fitting of responses at equilibrium according to 1:1 Langmuir binding model (K_D: 15 ± 3 μM).

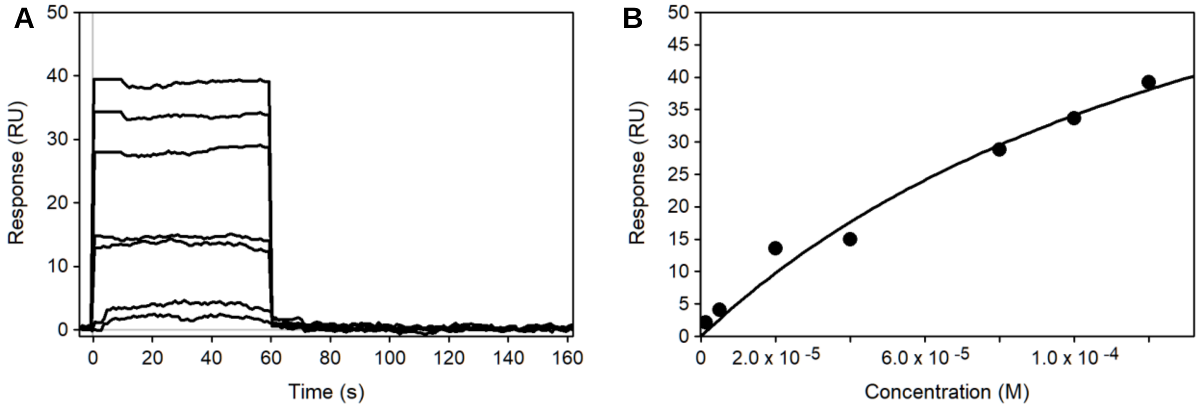

Supplementary Figure 76 (A) Overlay of sensorgrams of **2** injected at concentrations 1.25−120 μM over an immobilised Nsp10; (B) Fitting of responses at equilibrium according to 1:1 Langmuir binding model (K_D: 160 ± 40 μM)

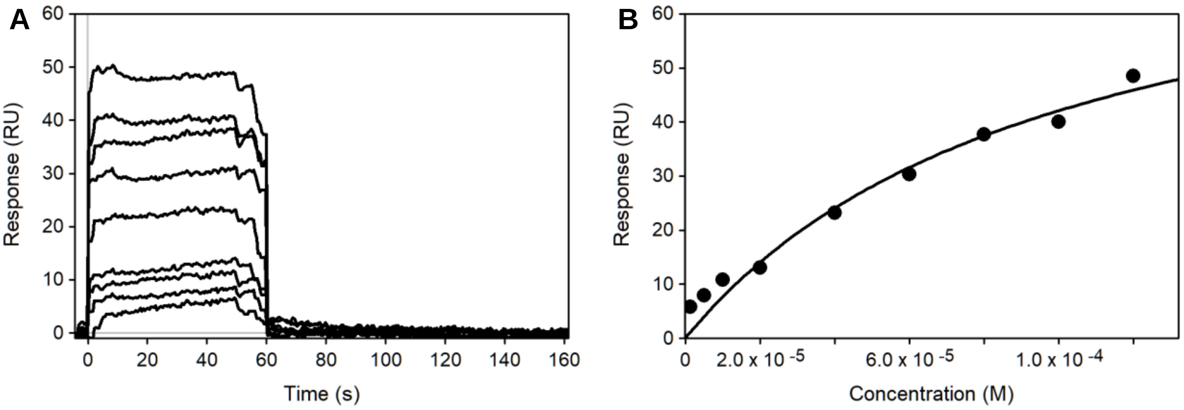

Supplementary Figure 77 (A) Overlay of sensorgrams of **3** injected at concentrations 1.25−120 μM over an immobilised Nsp10; (B) Fitting of responses at equilibrium according to 1:1 Langmuir binding model (K_D: 100 ± 10 μM)

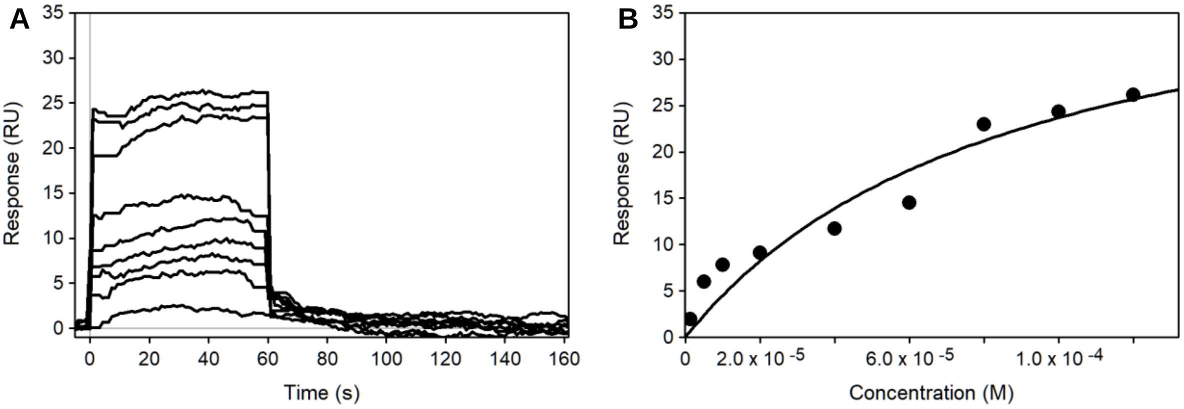

Supplementary Figure 78 (A) Overlay of sensorgrams of **4** injected at concentrations 1.25−120 μM over an immobilised Nsp10; (B) Fitting of responses at equilibrium according to 1:1 Langmuir binding model (K_D: 90 ± 20 μM).

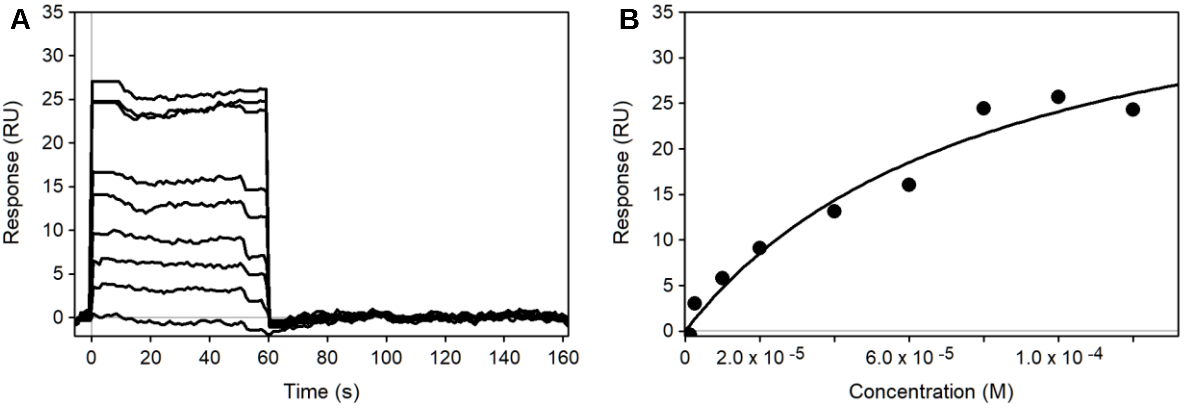

Supplementary Figure 79 (A) Overlay of sensorgrams of **5** injected at concentrations 1.25−120 μM over an immobilised Nsp10; (B) Fitting of responses at equilibrium according to 1:1 Langmuir binding model (K_D: 80 ± 10 μM).

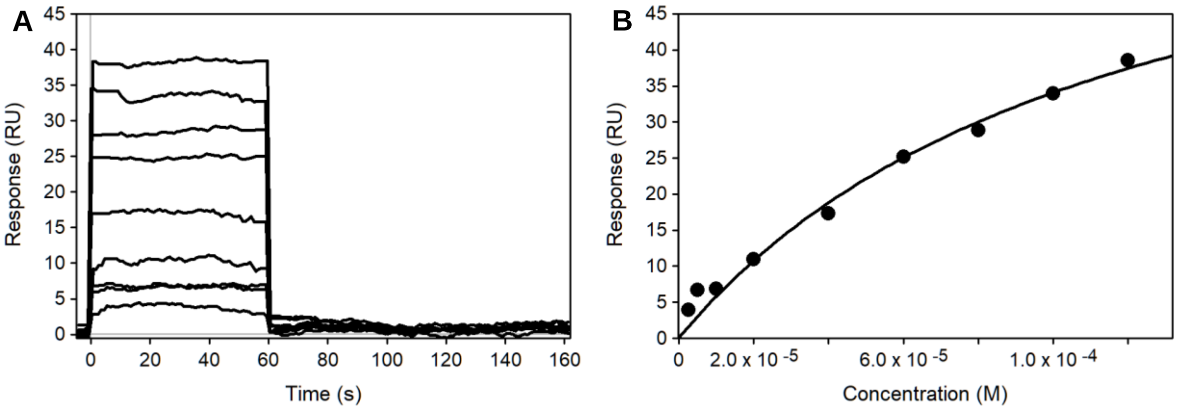

Supplementary Figure 80 (A) Overlay of sensorgrams of **6** injected at concentrations 1.25−120 μM over an immobilised Nsp10; (B) Fitting of responses at equilibrium according to 1:1 Langmuir binding model (K_D: 120 ± 10 μM).

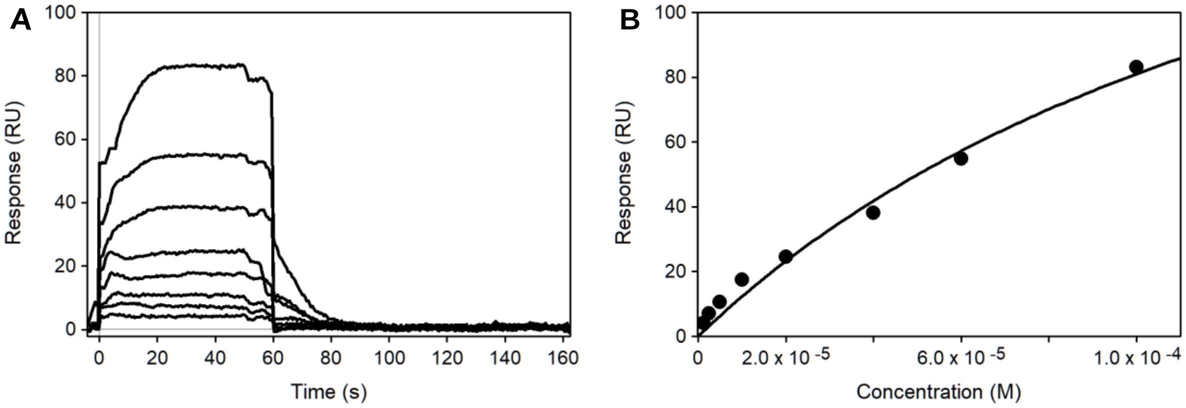

Supplementary Figure 81 (A) Overlay of sensorgrams of **7** injected at concentrations 1.25−100 μM over an immobilised Nsp10; (B) Fitting of responses at equilibrium according to 1:1 Langmuir binding model (K_D: 170 ± 20 μM).

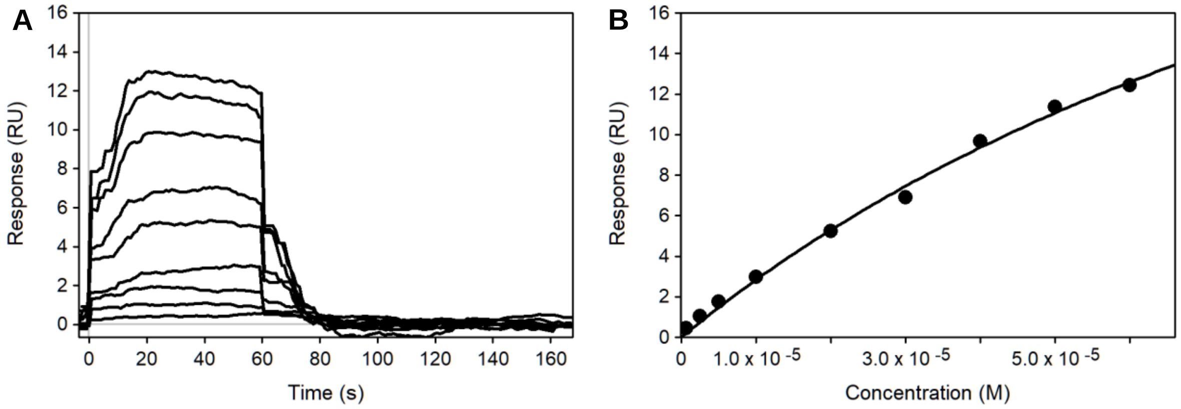

Supplementary Figure 82 (A) Overlay of sensorgrams of **8** injected at concentrations 0.625−60 μM over an immobilised Nsp10; (B) Fitting of responses at equilibrium according to 1:1 Langmuir binding model (K_D: 130 ± 10 μM).

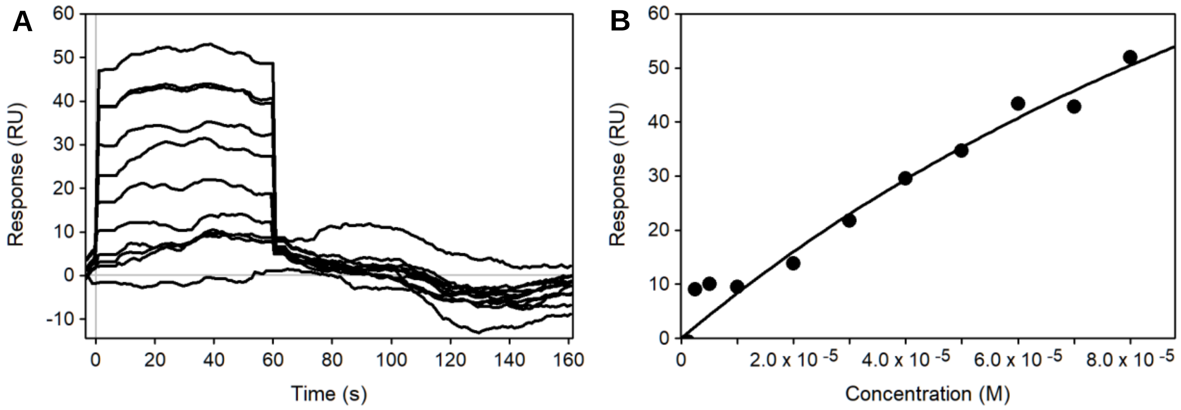

Supplementary Figure 83 (A) Overlay of sensorgrams of **12** injected at concentrations 1.25−80 μM over an immobilised Nsp10; (B) Fitting of responses at equilibrium according to 1:1 Langmuir binding model (K_D: 200 ± 60 μM).
